# Supplementary material for: Hybrid calculation of hadronic vacuum polarization in muon g − 2 to 0.48%
Source: Nature. 2026 Apr 22;653(8114):373–7. doi: 10.1038/s41586-026-10449-z (PMC13171618; doi:10.1038/s41586-026-10449-z)
Supplement: Supplementary file 1 — Supplementary Methods with Sections 1–10, including Supplementary Figs. 1–23, Supplementary Tables 1–24 and Supplementary References. [file 41586_2026_10449_MOESM1_ESM.pdf]

nature portfolio

https://doi.org/10.1038/s41586-026-10449-z

Supplementary information

Hybrid calculation of hadronic vacuum polarization in muon $g-2$ to 0.48%

In the format provided by the authors and unedited

Supplementary Information

Hybrid calculation of hadronic vacuum polarization in muon g-2 to 0.48%

A. Boccaletti[1,2], Sz. Borsanyi[1], A. Cotellucci[2], M. Davier[3], Z. Fodor[4,5,1,2,6,7,*], F. Frech[1], A. Gérardin[8], D. Giusti[2,9], A.Yu. Kotov[2], L. Lellouch[8], Th. Lippert[2], A. Lupo[8], B. Malaescu[10], S. Mutzel[8,11], A. Portelli[12,13], A. Risch[1], M. Sjö[8], F. Stokes[2,14], K.K. Szabo[1,2], B.C. Toth[1,2], G. Wang[8], Z. Zhang[3]

[1] Department of Physics, University of Wuppertal, D-42119 Wuppertal, Germany
[2] Jülich Supercomputing Centre, Forschungszentrum Jülich, D-52428 Jülich, Germany
[3] IJCLab, Université Paris-Saclay et CNRS/IN2P3, Orsay, 91405, France
[4] Physics Department, Pennsylvania State University, University Park, PA 16802, USA
[5] Institute for Computational and Data Sciences, Pennsylvania State University, University Park, PA 16802, USA
[6] Institute for Theoretical Physics, Eötvös University, H-1117 Budapest, Hungary
[7] University of California, San Diego, 9500 Gilman Drive, La Jolla, CA 92093, USA
[8] Aix Marseille Univ, Université de Toulon, CNRS, CPT, IPhU, Marseille, France
[9] Fakultät für Physik, Universität Regensburg, 93040, Regensburg, Germany
[10] LPNHE, Sorbonne Université, Université Paris Cité, CNRS/IN2P3, Paris, 75252, France
[11] Laboratoire de Physique de l'Ecole Normale Supérieure, Mines Paris - PSL, CNRS, Inria, PSL Research University, Paris, France
[12] School of Physics and Astronomy, University of Edinburgh, Edinburgh EH9 3JZ, United Kingdom
[13] RIKEN Center for Computational Science, Kobe 650-0047, Japan
[14] Special Research Centre for the Subatomic Structure of Matter, Department of Physics, University of Adelaide, South Australia 5005, Australia

Contents

S1 Configurations and measurements **3**
S1.1 Action and ensembles . . . 3
S1.2 Taste violation . . . 6

S2 Scale setting with the omega baryon mass **7**
S2.1 Omega propagator measurements . . . 7
S2.2 Omega propagator fits . . . 8
S2.3 Determination of w_0 using the Omega mass . . . 10

S3 Scale setting with the pion decay rate **14**
S3.1 Pion propagator measurements and fits . . . 14
S3.2 Finite-size effects . . . 15
S3.3 Electromagnetic effects . . . 16
S3.4 Determination of w_0 using the pion decay rate - formulae . . . 17
S3.5 Determination of w_0 using the pion decay rate - results . . . 20

S4 Physical point and isospin decomposition **23**
S4.1 Physical point . . . 23
S4.2 Isospin decomposition . . . 25
S4.3 Kaon mass decomposition in different schemes . . . 25

S5 Analysis procedure **26**
S5.1 Fit functions . . . 26
S5.2 Distribution of observables . . . 27
S5.3 Combining distributions via random sampling . . . 28

S6 Window observables **29**
S6.1 Blinding . . . 30
S6.2 Short-distance window . . . 31
S6.3 Intermediate-distance window . . . 34
S6.4 Long-distance window $15 - 19$ . . . 37
S6.5 Long-distance window $10 - 28$ . . . 37
S6.6 All-distance window $00 - 28$ . . . 41
S6.7 Long-distance window $10 - \infty$ and comparison with 2020 result . . . 45
S6.8 Strange and charm contributions . . . 48
S6.9 Other contributions, total and comparison with 2020 result . . . 49

S7 Finite-size effects **52**

S8 Data-driven checks of finite-volume corrections **54**

S9 Verification of isospin-breaking contributions **57**
S9.1 Isospin breaking contributions to a_μ^{light} . . . 57
S9.2 Strong-isospin-breaking contribution to a_μ^{disc} . . . 59
S9.3 Corrections . . . 59

S10 Long-distance contributions **61**

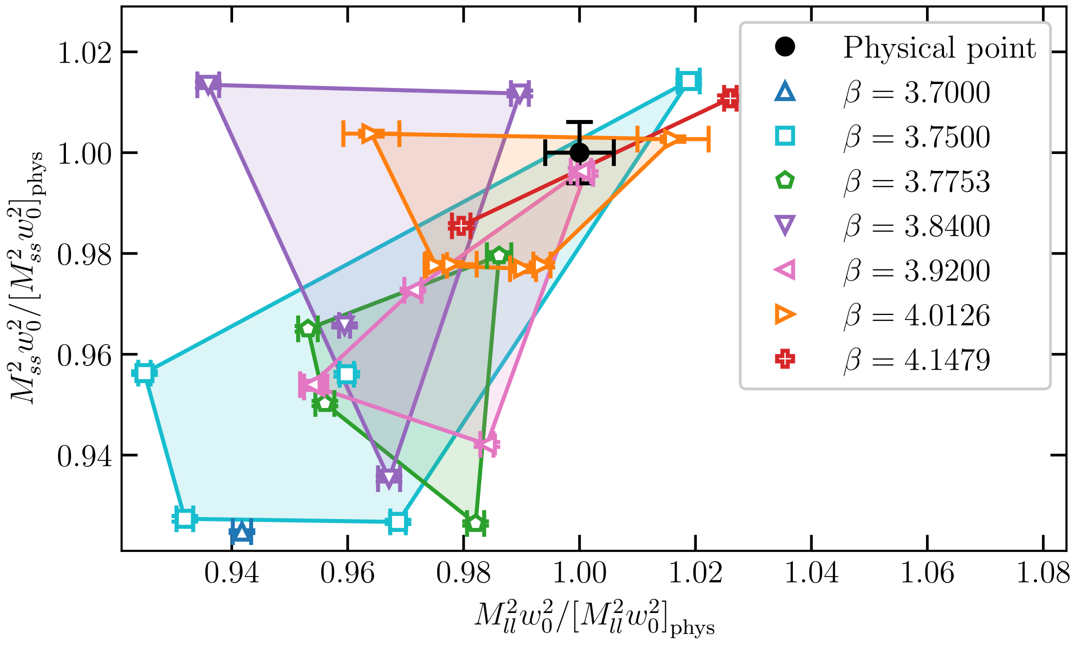

Figure S1: Landscape of our ensembles. The horizontal and vertical axes are the squared pseudo-scalar masses, M_{ll}^2 and M_{ss}^2, in units of the w_0-scale, both normalized to the central value of their respective physical point. Different colours denote different lattice spacings. The black point denotes the (isospin-symmetric) physical point, with error bars corresponding to the uncertainties from our determination of the M_{ll}, M_{ss} and w_0 parameters in physical units. (Figures in this work were produced with the aid of Matplotlib [58].)

S1 Configurations and measurements

S1.1 Action and ensembles

We perform simulations using a `4stout` lattice action, given by the tree-level Symanzik gauge action [53] and a one-link staggered fermion action. Where the gauge link appears in the fermion action, we apply four steps of stout smearing [54] with a smearing parameter of $\rho = 0.125$. Some of the measurements are performed on supercomputers with multi-GPU compute nodes, here we use the highly-optimized QUDA [55, 56] and Qlattice [57] software suites.

We use $2+1+1$ dynamical flavours with equal up and down quark masses $m_u = m_d = m_l$. The mass parameters for the light, m_l, and the strange quark, m_s, are chosen to scatter around the physical point. This can be seen in Figure S1, where we show the landscape of our ensembles in the plane of the light and strange quark masses. The charm mass parameter is set by the ratio $m_c/m_s = 11.85$, which is taken from the $c\bar{c}$ current analysis in [59]. This value is within one per-cent of the most recent lattice average from FLAG [60–64]. We use seven different values for the gauge coupling parameter β. The corresponding lattice spacings and the set of ensembles at each lattice spacing, together with the number of configurations are listed in Table S1. Most of the ensembles in this study were also used in our earlier work on the magnetic moment of the muon [1]. Since then we added a finer lattice spacing, corresponding to $\beta = 4.1479$, with two different ensembles, which bracket the physical point in both the light and the strange-quark mass.

To set the scale and the physical point we use the Wilson-flow-based observable w_0 and the masses of $q\bar{q}$ connected pseudo-scalar mesons with $q = u, d, s$. The physical values of these observables were computed in our earlier work [1], where we used the experimental values of the hadron masses π^0, K^+, K^0 and Ω^- as inputs. A more detailed discussion about our setting of the physical point is given in Section S4.

β	a [fm]	$L/a \times T/a$	tag	am_s	m_s/m_l	#confs
3.7000	0.1315	48×64	dir00	0.057291	27.899	904
3.7500	0.1191	56×96	dir00	0.049593	28.038	315
			dir01	0.049593	26.939	516
			dir02	0.051617	29.183	504
			dir03	0.051617	28.038	522
			dir05	0.055666	28.083	215
3.7753	0.1116	56×84	dir00	0.047615	27.843	510
			dir01	0.048567	28.400	505
			dir02	0.046186	26.469	507
			dir03	0.049520	27.852	385
3.8400	0.0952	64×96	dir00	0.043194	28.500	510
			dir02b	0.043194	30.205	436
			dir04	0.040750	28.007	1503
			dir05	0.039130	26.893	500
3.9200	0.0787	80×128	dir02	0.032440	27.679	506
			dir04	0.034240	27.502	512
			dir01b	0.032000	26.512	1001
			dir02b	0.032440	27.679	327
			dir03b	0.033286	27.738	1450
			dir04b	0.034240	27.502	500
4.0126	0.0640	96×144	phys1	0.026500	27.634	446
			phys2	0.026500	27.124	551
			phys1b	0.026500	27.634	2248
			phys2b	0.026500	27.124	1000
			phys3	0.027318	27.263	985
			phys4	0.027318	28.695	1750
4.1479	0.0483	128×192	phys1	0.019370	27.630	2792
			phys2	0.019951	27.104	2225

Table S1: List of the ensembles used in this work, with gauge coupling, lattice spacing, lattice size, ensemble tag, strange-quark mass, mass ratio of strange and light quarks and number of configurations. The numbers are rounded to the accuracy provided by the number of displayed digits.

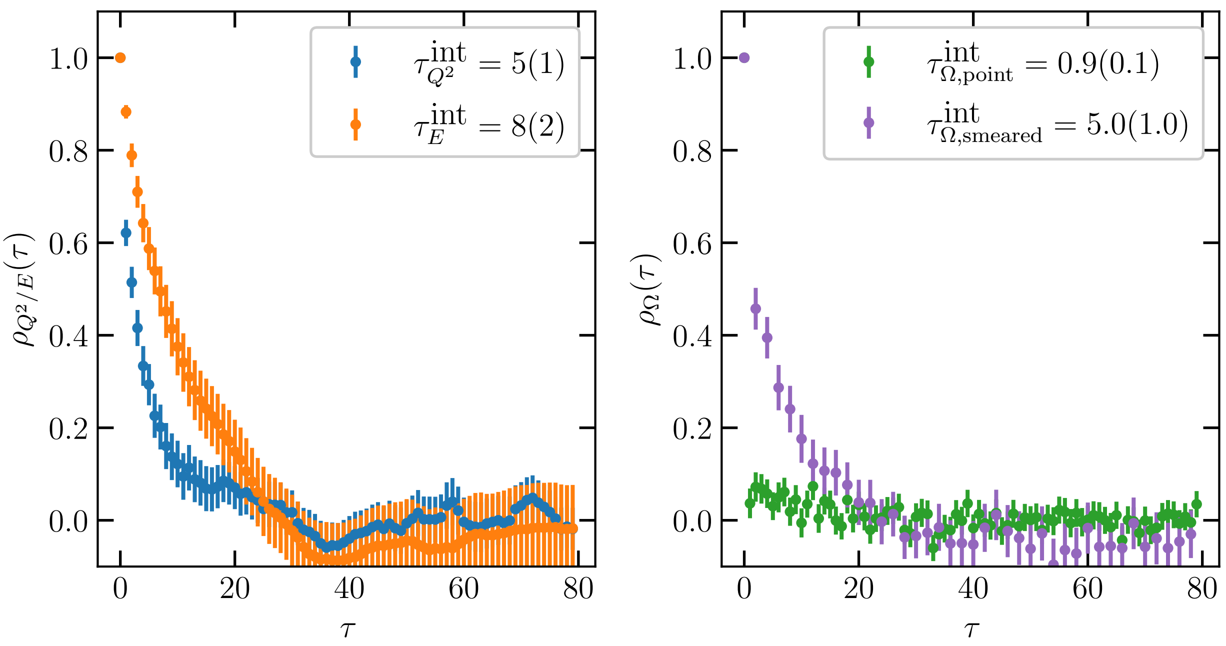

Figure S2: Left panel: normalized autocorrelation function of the energy density E and topological charge squared Q^2, both computed at a gradient-flow time of w_0^2 on one of the ensembles at $\beta = 4.1479$. Right panel: normalized autocorrelation function of the $\Omega_{\rm VI}$ hadron correlator with point and smeared sources. The correlator is taken at $t = 1.5$ fm time separation, which is located in the middle of the fitting window. The plots also include the integrated autocorrelation times, given in units of configurations.

The configurations are generated with a Rational Hybrid Monte Carlo algorithm [65] including force gradient [66], multiple timescales [67] and Hasenbusch preconditioning [68]. The configurations are separated by 10 unit length trajectories. In Figure S2 we show normalized autocorrelation functions, denoted here by $\rho(\tau)$, for the topological charge squared [69], smeared energy density and Omega propagator. The charge is computed using the standard clover discretization of the topological charge density at a gradient-flow time of w_0^2, which corresponds to a smearing radius of about $0.5\,\rm fm$. The same flow-time is used for the smeared energy density. The definition of $\Omega_{\rm VI}$ operator is given in Section S2. The autocorrelation function and its error are computed using the `pyerrors` package [70]. The integrated autocorrelation times are also given in Figure S2, the largest of which corresponds to the smeared energy density.

We use jackknife resampling to calculate the statistical errors. To suppress the auto-correlation between data from subsequent configurations we introduce a blocking procedure. It is very convenient to use an equal number of blocks for all ensembles. In this work we use 48 blocks. With this choice we have typically 10 configurations or more in a block, which is larger than the autocorrelation time of any of the quantities we consider even on our finest ensembles, as shown in Figure S2. For the blocks we apply the delete-one principle, resulting in 48 jackknife samples plus the full sample.

Topological properties of QCD were investigated with the `4stout` action in Ref. [71], where the topological susceptibility χ was computed for a wide range of lattice spacings. The continuum extrapolation of χ is notoriously difficult, because of the absence of exact zero modes of the staggered Dirac operator at finite lattice spacing. The behaviour towards the continuum can be much improved by rescaling χ with the square of the ratio of the Goldstone and taste singlet pion masses. On our finest lattice we find $\chi = 0.0358(29)$ fm^4 for the unimproved and $\chi = 0.0299(24)$ fm^4 for the improved susceptibility. These numbers nicely fit on the continuum extrapolation curves presented in Figure S1 of Ref. [71]. In that work it was found, that the continuum extrapolated value agrees well with the prediction of chiral perturbation theory.

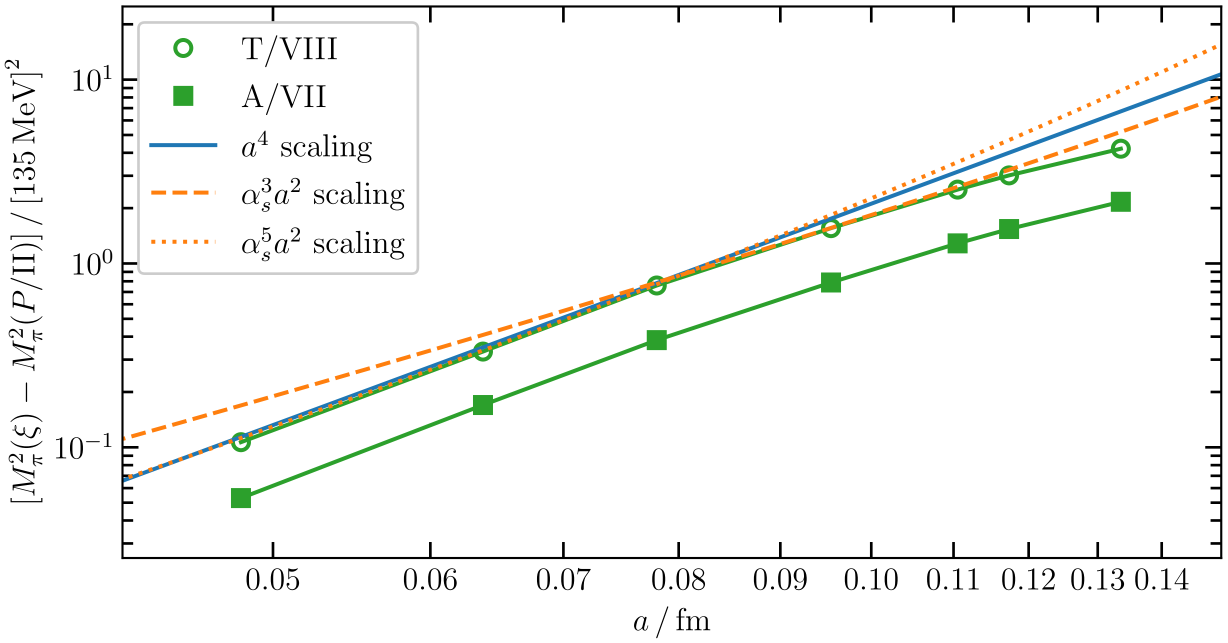

Figure S3: Taste violation as a function of lattice spacing, for axial-vector A and tensor T tastes. The Roman-number labelling of the tastes is taken from [72], e.g. the pseudo-scalar taste P corresponds to II.

S1.2 Taste violation

An important aspect of these computations is the lattice artefact related to the taste symmetry violation of staggered fermions. This makes pseudo-scalar mesons heavier than in the continuum, depending on their taste quantum number. In particular the masses of pions and eta mesons built from light quark flavours are given as:

$$M_\pi^2(\xi) = M_{ll}^2 + \Delta_{KS}(\xi) \quad \text{and} \quad M_\eta^2(\xi) = M_{ll}^2 + \Delta_{KS}(\xi) + \tfrac{1}{2}\delta_{KS}(\xi) \tag{S1}$$

where M_{ll}^2 is the squared mass of the usual pseudo-Goldstone pion. The pions have only connected contractions, whereas the etas also contain disconnected contributions. The ξ stands for one of the sixteen meson tastes of the taste $SU(4)$ group. In practical lattice simulations these group into multiplets of the $SO(4)$ group to a very good approximation, so the meson tastes are conventionally labelled by P, A, T, V, I. The P taste corresponds to the pseudo-Goldstone pion, for which $\Delta_{KS}(P)$ and $\delta_{KS}(P)$ are zero.

We compute the taste-symmetry breaking terms $\Delta_{KS}(\xi)$ by comparing the masses of different meson tastes on our ensembles. For this purpose we use an average "flavour-symmetric" valence quark mass of $\frac{1}{3}(2m_l + m_s)$. In Figure S3 we show this comparison for the A and T tastes as a function of the lattice spacing. We observe a decrease with approximately the fourth power of the lattice spacing towards our finer lattices. This is much faster than the $\alpha_s a^2$ expected from naive scaling, where $\alpha_s(a)$ is the strong coupling constant at the lattice cutoff scale. The falloff is actually consistent with an $\alpha_s^n(a)a^2$ type behaviour with $n = 3$ on the coarser and with $n = 5$ towards the finer lattices.

The taste-symmetry breaking terms $\delta_{KS}(\xi)$ are related to the eta mesons. $\delta_{KS}(T) = 0$ in the $SO(4)$ approximation, $\delta_{KS}(I)$ arises from the chiral anomaly, and $\delta_{KS}(A)$ and $\delta_{KS}(V)$ are called hairpin parameters. These have never been computed directly, since the disconnected contributions are computationally demanding. Here we assume that these artefacts decrease with the same rate as $\Delta_{KS}(\xi)$; the same assumption was made in Ref. [73].

β	$N_{\rm Wptl}$	$N_{\rm 3d}$	t_p	t_a	t_b	range #1	range #2	# pt, sm sources
3.7000	24	32	1	4	7	7...15	8...15	28928, 229376
3.7500	30	40	1	4	7	8...18	9...18	66208, 530176
3.7553	34	46	1	4	7	9...19	10...19	61024, 488192
3.8400	46	62	2	4	9	10...20	11...20	125440, 2807552
3.9200	67	90	2	6	9	12...25	13...25	137472, 3038720
4.0126	101	135	3	6	9	15...30	16...30	223360, 4235520
4.1479	178	238	5	6	11	19...40	21...40	160544, 2068736

Table S2: Parameters of our procedure to obtain Ω mass: number of Wuppertal and stout smearing steps; the parameters t_p, t_a and t_b of the GEVP procedure and two different fit ranges, given by start and end points $t_{\rm min}$ and $t_{\rm max}$. Definitions are given in the text. In the last column the total number of measurements with point and smeared sources are given.

S2 Scale setting with the omega baryon mass

S2.1 Omega propagator measurements

To extract the mass of the positive-parity, ground-state Ω baryon, we use a similar strategy as in our previous work [1]. In particular we consider three different operators [72, 74, 75]:

$$\begin{aligned}
\Omega_{\rm VI}(t) &= \sum_{x_k even} \epsilon_{abc} \left[S_1\chi_a S_{12}\chi_b S_{13}\chi_c - S_2\chi_a S_{21}\chi_b S_{23}\chi_c + S_3\chi_a S_{31}\chi_b S_{32}\chi_c\right](x) \\
\Omega_{\rm XI}(t) &= \sum_{x_k even} \epsilon_{abc} \left[S_1\chi_a S_2\chi_b S_3\chi_c\right](x) \\
\Omega_{\rm Ba}(t) &= \left[2\delta_{\alpha1}\delta_{\beta2}\delta_{\gamma3} - \delta_{\alpha3}\delta_{\beta1}\delta_{\gamma2} - \delta_{\alpha2}\delta_{\beta3}\delta_{\gamma1} + (\cdots\beta\leftrightarrow\gamma\cdots)\right] \\
&\quad \sum_{x_k even} \epsilon_{abc} \left[S_1\chi_{a\alpha} S_{12}\chi_{b\beta} S_{13}\chi_{c\gamma} - S_2\chi_{a\alpha} S_{21}\chi_{b\beta} S_{23}\chi_{c\gamma} + S_3\chi_{a\alpha} S_{31}\chi_{b\beta} S_{32}\chi_{c\gamma}\right](x)
\end{aligned} \tag{S2}$$

Here, $\chi_a(x)$ is the strange-quark field with colour index a and $\chi_{a\alpha}(x)$ with $\alpha = 1, 2, 3$ the additional "flavour" index introduced by Bailey [75]. The operator S_μ performs a symmetric, gauge-covariant shift in direction μ, while $S_{\mu\nu} \equiv S_\mu S_\nu$. $\Omega_{\rm VI}$ and $\Omega_{\rm XI}$ couple to two different tastes of the Ω baryon and $\Omega_{\rm Ba}$ only couples to a single taste. In the continuum limit the masses of these states become degenerate. In our analyses we include all three in order to assign a systematic to the choice of the operator.

Beside point sources we also use smeared ones to construct the propagator. Smearing changes the excited state contamination and including smeared propagators in the analysis makes a more reliable extraction of the ground state possible. These propagators are constructed by applying spatial Wuppertal smearing [76] on a source ψ vector

$$[\hat{W}\psi]_x = (1-\sigma)\psi_x + \frac{\sigma}{6} \sum_{\mu=1,2,3} \left(U^{\rm 3d}_{\mu,x} U^{\rm 3d}_{\mu,x+\mu} \psi_{x+2\mu} + U^{\rm 3d,\dagger}_{\mu,x-\mu} U^{\rm 3d,\dagger}_{\mu,x-2\mu} \psi_{x-2\mu} \right) \tag{S3}$$

with smearing parameter $\sigma = 0.5$. The spatial derivatives involve two hops in order to preserve the staggered symmetries of the operators. They also include a smeared-gauge field $U^{\rm 3d}$ obtained by applying $N_{\rm 3d}$ spatial stout smearing steps with smearing parameters 0.125. The smeared source is then obtained by applying the Wuppertal-smearing procedure $N_{\rm Wptl}$ times on a point source. The number of smearing steps, see Table S2, depends on the β in such a way to keep the effective smearing radius of the procedure approximately constant in physical units.

To enhance the signal, we calculate the Ω propagators using 256 or 512 smeared and 32 point source fields per gauge configuration; the total number of measurements for each β are given in the last column of Table S2. For each source field we select a random time slice, which, in turn, is populated with eight

independent $\mathbb{Z}_3$ random point sources at $(0,0,0)$, $(L/2,0,0)$, $\cdots$ and $(L/2,L/2,L/2)$. The idea with eight sources was originally proposed in [77].

S2.2 Omega propagator fits

Our mass extraction procedure combines the Generalized Eigenvalue Problem (GEVP) approach, see Ref. [78] and references therein, with the Generalized Pencil-of-Function approach proposed in Ref. [79]. We first apply a folding transformation to the original hadron propagator H_t:

$$H_t \to \begin{cases} \frac{1}{2}\left[H_t + (-1)^{t+1}H_{T-t}\right] & 0 < t < \frac{T}{2} \\ H_t & t = 0 \text{ or } t = \frac{T}{2} \end{cases}, \tag{S4}$$

where the staggered phase factor $(-1)^{t+1}$ ensures the parity is consistent between the forward and backward-propagating states in the folding. Then for each time slice t we construct the following 6×6 matrix:

$$\mathbf{H}(t) = \left(\begin{array}{cc|cccc} H^{pp}_{t+2t_p+0} & H^{pp}_{t+2t_p+1} & H^{ps}_{t+t_p+0} & H^{ps}_{t+t_p+1} & H^{ps}_{t+t_p+2} & H^{ps}_{t+t_p+3} \\ H^{pp}_{t+2t_p+1} & H^{pp}_{t+2t_p+2} & H^{ps}_{t+t_p+1} & H^{ps}_{t+t_p+2} & H^{ps}_{t+t_p+3} & H^{ps}_{t+t_p+4} \\ \hline H^{sp}_{t+t_p+0} & H^{sp}_{t+t_p+1} & H^{ss}_{t+0} & H^{ss}_{t+1} & H^{ss}_{t+2} & H^{ss}_{t+3} \\ H^{sp}_{t+t_p+1} & H^{sp}_{t+t_p+2} & H^{ss}_{t+1} & H^{ss}_{t+2} & H^{ss}_{t+3} & H^{ss}_{t+4} \\ H^{sp}_{t+t_p+2} & H^{sp}_{t+t_p+3} & H^{ss}_{t+2} & H^{ss}_{t+3} & H^{ss}_{t+4} & H^{ss}_{t+5} \\ H^{sp}_{t+t_p+3} & H^{sp}_{t+t_p+4} & H^{ss}_{t+3} & H^{ss}_{t+4} & H^{ss}_{t+5} & H^{ss}_{t+6} \end{array}\right), \tag{S5}$$

where type in H_t^{type} denotes the different source-sink combinations, such as point-point, smear-point, point-smear and smear-smear, labelled with pp, sp, ps and ss. We introduce an additional time shift t_p in case of the point source operator to suppress its excited states. The time shifts between different rows and columns are needed to fully resolve the negative parity states, which have a negative amplitude.

For a given t_a and t_b, let $\lambda(t_a,t_b)$ be an eigenvalue and $v(t_a,t_b)$ an eigenvector of the following GEVP:

$$\mathbf{H}(t_a)v(t_a,t_b) = \lambda(t_a,t_b)\mathbf{H}(t_b)v(t_a,t_b)\ . \tag{S6}$$

The ground state corresponds to the largest eigenvalue. The propagator corresponding to the eigenvector v is given by vector-matrix-vector product:

$$P(t;t_a,t_b) = v^\dagger(t_a,t_b)\mathbf{H}(t)v(t_a,t_b)\ . \tag{S7}$$

This correlation function can be fitted to an exponential function $A\exp(-Mt)$ with amplitude A and mass M. Note, that backward propagating states have negligible contribution for the time-slices that we work with. From $P(t)$ one can also construct an effective mass in the standard way. The tuneable parameters of the procedure are t_a and t_b for specifying the GEVP, as well as the fit range $[t_{\min}, t_{\max}]$ for the exponential fitting.

In Figure S4 we show the effective masses obtained with the GEVP procedure for an ensemble at $\beta = 4.1479$. This plot shows the excited states. The phenomenological interpretation of these is non-trivial, since they are hadron resonances decaying into scattering states. As an illustration we show the excited, negative-parity Ω baryon from the Belle experiment [80] with a mass of $2012.4(7)(6)$ MeV. As shown in Figure S5, the ground state is well resolved with a per-mill level of precision.

We perform the exponential fit taking into account the correlations between different timeslices in the $P(t)$ propagator. For that we construct the covariance matrix with 200 jackknife samples, instead of our usual choice of 48 samples, to stabilize its inversion. The smaller block-size should not be problematic with the Ω, given its autocorrelation time provided in Section S1. When inverting the covariance matrix we apply a singular value decomposition and regulate the smallest eigenvalues of the correlation matrix

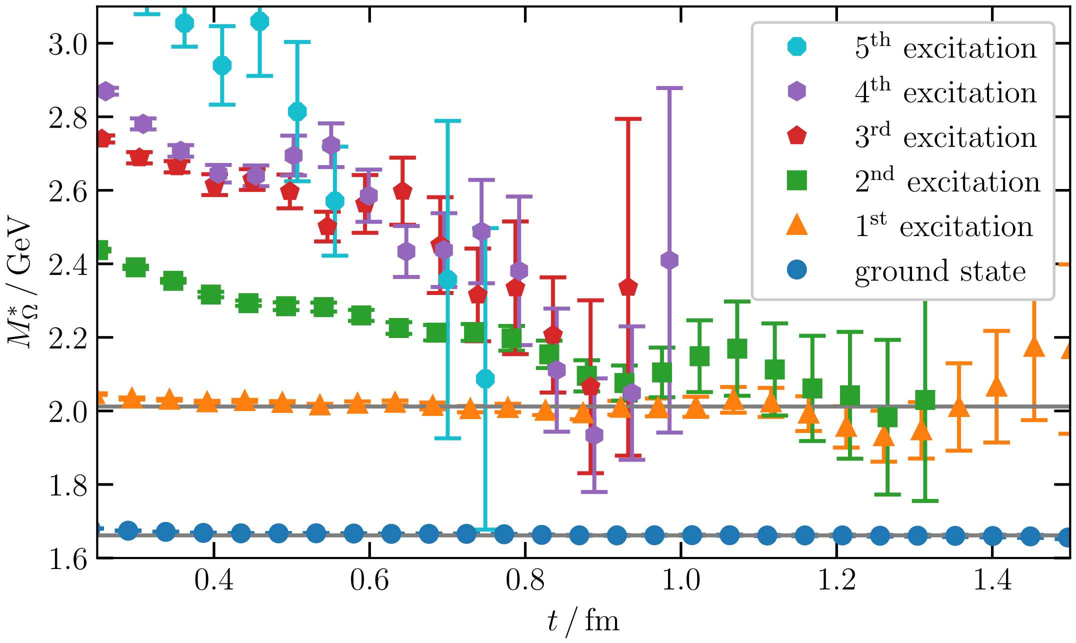

Figure S4: Ω baryon effective masses extracted from the GEVP for five excited states and the ground state on an ensemble at $\beta = 4.1479$. The blue band through the ground-state points is obtained from a single exponential fit to the ground state propagator. The grey horizontal line through the first excited state corresponds to the mass of the recently observed excited Ω baryon by the Belle experiment [80]. To convert the numbers into physical units we used $a = 0.0483\,\mathrm{fm}$.

as described in Appendix A.1 of Ref. [81]. In an uncorrelated fit or in a correlated fit with regulated eigenvalues the statistical interpretation of the fit quality becomes questionable. Recently an improved estimator for the fit quality was given in [82], which we call Q-value here. It can be employed both in the uncorrelated and correlated cases.

The time range in the exponential fit is going to be chosen by the Q-value of the fit. For each operator in Equation (S2) we compute the Q-values on all of our ensembles for several different fit ranges $[t_{\mathrm{min}}, t_{\mathrm{max}}]$. In the left panel of Figure S6 we show the cumulative distribution function of the Q-values over all of our ensembles for some selected fit-ranges and for the Ω_{VI} operator. For a given fit range the Q-values will follow a uniform distribution if the ground state correlator $P(t)$ can be described by a single exponential $A\exp(-Mt)$. To decide if the observed CDF is uniform, we use a one-sided Kolmogorov-Smirnov test with the uniform distribution of the Q-values as null-hypothesis. We vary the fit-ranges and compute the Kolmogorov-Smirnov significance. These are shown in a heat-map format in the right panel of Figure S6. We choose to work with the fit-range $[0.9\,\mathrm{fm}, 2.0\,\mathrm{fm}]$. To estimate the systematic error related to the fit-range we also use one with a later start, $[1.0\,\mathrm{fm}, 2.0\,\mathrm{fm}]$. Both ranges are plotted on the heatmap figure, with red crosses.

To investigate the excited state contamination of the ground state masses, we perform correlated fits to the ground state propagator with four exponentials instead of one. We introduce 100 MeV wide priors on the excited states, for the central values we use masses of Ω resonances taken from the Particle Data Book. This choice of priors assumes that the dominant contaminations come from states near the resonance energies. This is motivated by the local nature of our Omega operators, but ignores possible contributions from the non-resonant scattering states discussed at the end of this section. Results of these fits are shown in the effective mass plot of Figure S5, in case of our finest two ensembles. The ground state mass is in good agreement between the single-state and four-state fits.

The extracted ground state energies for all of our ensembles are given in Figure S7. We reach better than 0.1% precision on our finest lattice. We see an increase of the precision towards finer lattice spacings, which can be partly explained by the increase in the number of measurements with β. To confirm the

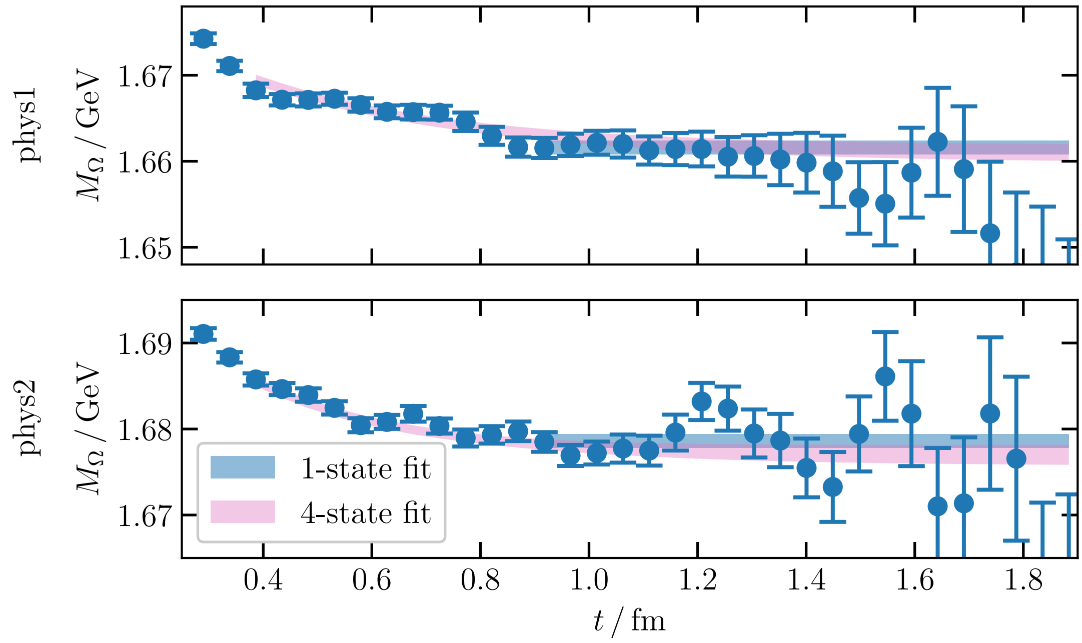

Figure S5: Ω baryon ground state effective masses extracted from the GEVP for the two ensembles at our finest lattice spacing, $\beta = 4.1479$. The blue horizontal band shows the ground state mass obtained from a single exponential fit; the pink, curved band corresponds to the effective mass of a fit with four exponentials.

robustness of our procedure, we performed fits also using the previously discussed four-state fits. The results of all these fits are shown in Figure S7, and we observe a good agreement across the different determination procedures.

The determination of the ground state baryon mass, as detailed in this Section, has a caveat[2]. It does not account for the presence of non-resonant, scattering states in the excited state spectrum, like (Ξ, K), (Ω, π, π) and other multi-hadron combinations. Though our single-hadron operators are expected to couple weakly to these states, we cannot guarantee that the scattering state contamination remains within our quoted precision. A similar problem occurs in the case of nucleon propagators, where the (Δ, π) states distort the ground state determination. This problem has been investigated in chiral perturbation theory [83, 84].

S2.3 Determination of w_0 using the Omega mass

In order to compute the physical value of the gradient-flow scale w_0 in full QCD plus QED, we use the analysis strategy from our 2020 work. There we parameterize the quark mass and electromagnetic coupling dependence of the $w_0 M_\Omega$ product as

$$w_0 M_\Omega = A + B\ M_\Omega^{-2} M_{ud}^2 + C\ M_\Omega^{-2}(M_{us}^2 + M_{ds}^2 - M_{ud}^2)/2 + E\ e_v^2 + F\ e_v e_s + G\ e_s^2 \qquad \text{(S8)}$$

where e_v and e_s are the electromagnetic couplings of the valence and sea quarks. We called these fits "Type-I" in our work. The procedure determines the parameters $A, \ldots, F$ by fitting measurements over several ensembles. The physical value of $[w_0 M_\Omega]_{\text{qcd+qed}}$ is obtained by substituting the physical values of the M_{ud}, M_{us}, M_{ds} and M_Ω in the above formula. Then dividing by the value $M_\Omega = 1672.45$ MeV [85] yields the physical value of the gradient flow scale $[w_0]_{\text{qcd+qed}}$.

The parameters A, B and C can be determined from isospin-symmetric measurements. This has already been done in our 2020 work, here we redo this determination, where we add the 0.048 fm lattice

[2] We thank our referee for bringing this problem to our attention.

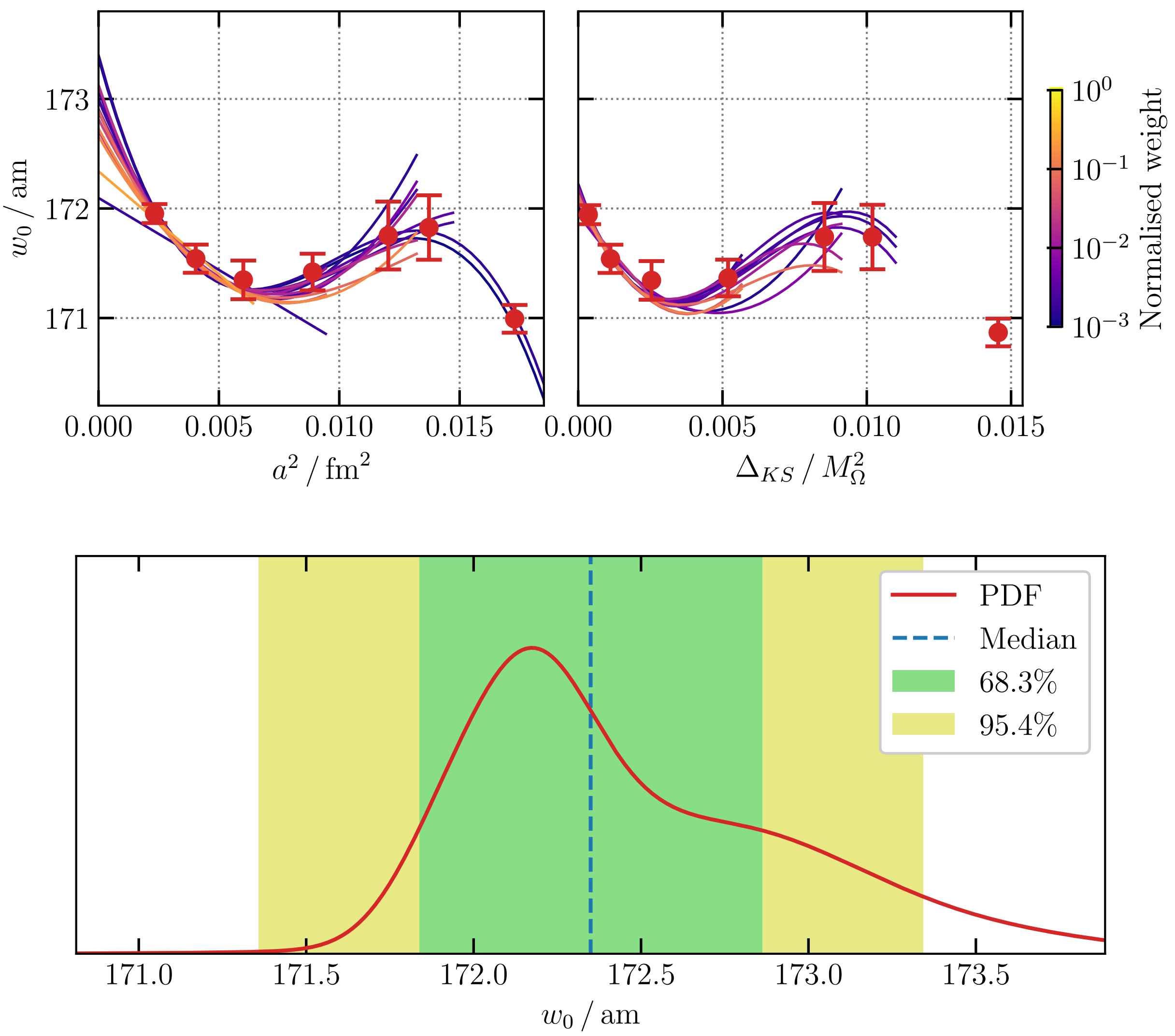

Median	172.35 am	
Total error	0.51 am	0.30 %
Statistical error	0.22 am	0.13 %
Systematic error	0.46 am	0.27 %
Pseudoscalar fits	0.01 am	$<$ 0.01 %
Omega baryon fits	0.24 am	0.14 %
Physical value of M_Ω	0.06 am	0.03 %
Lattice spacing cuts	0.09 am	0.05 %
Order of fit polynomials	0.17 am	0.10 %
Continuum parameter (Δ_{KS} or a^2)	0.30 am	0.17 %

Table S3: Gradient-flow scale w_0 in attometers using Omega baryon mass as input. Continuum extrapolations as a function of a^2 and of Δ_{KS} are shown in the first row. The highest-weighted fit is a function of a^2, and at a nominal lattice spacing of $0.1\,\mathrm{fm}$, the a^2 term contributes 4.7% of the total value, the a^4 term 5.5%, and the a^3 term 1.9%. The rest of the table shows the probability distribution function (PDF), and our error budget. The plot conventions, including small offsets of the points, are described in more detail in the first part of Section S6.

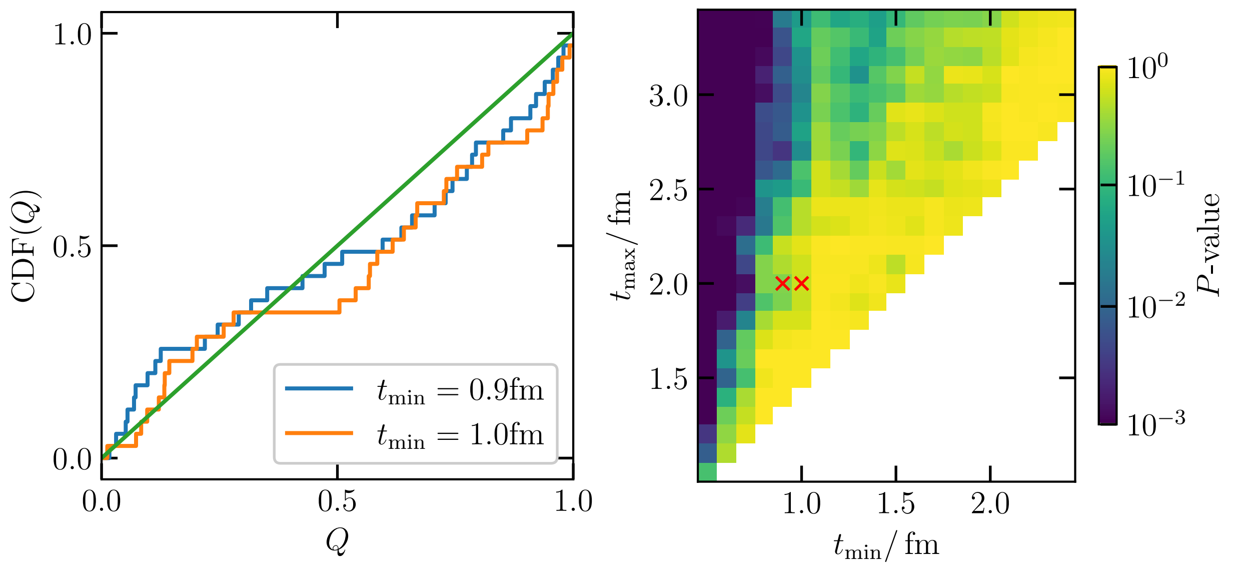

Figure S6: Left panel: cumulative distribution function (CDF) of Q-values [82] over all of our ensembles, where we show results for our two preferred fit-ranges for the $\Omega_{\rm VI}$ operator. The first fit-range, shown with blue, is $[0.9, 2.0]\,\rm fm$, whose Kolmogorov-Smirnov significance level is $P = 0.27$, i.e. the probability we get the observed CDF or anything worse is 27%, assuming a uniform distribution. We also use a second fit-range $[1.0, 2.0]\,\rm fm$, shown with orange, with $P = 0.57$. The green line represents the CDF of a hypothetical uniform distribution. Right panel: we show the Kolmogorov-Smirnov significance level for several different fit-ranges, $[t_{\rm min}, t_{\rm max}]$, the brighter/darker colours stand for better/worse significance. Our final choices for the above two fit-ranges are shown by the red crosses.

spacing into the analysis and also increase the statistics on coarser ensembles. The current analysis consists of 8640 fits, differing in the fit ranges of the pseudoscalars and the omega baryon, the cuts in the lattice spacing and the functional form of the fit function. We also vary the expansion variable between a naive a^2 and a $\Delta_{KS}(a)$ type dependence in the lattice spacing. As opposed to our earlier publication in 2020, where we used only quadratic polynomials of the expansion variable, this time we also include cubic ones. We have 8640 fits altogether, 83% of which have P-value of at least 0.1; Sample continuum extrapolations, probability distribution function and error budget are shown in Table S3. The w_0 values in the table are obtained by dividing the $w_0 M_\Omega$ product by the experimental value of the Ω baryon mass. The distribution has a narrower Gaussian peak at a lower value corresponding to Δ_{KS} fits and a broader at a higher value corresponding to a^2 fits.

From the above fits we obtain the value of w_0 in the isospin-symmetric point, which is defined by setting the hadron masses to the physical values and E, F and G to zero in Equation (S8):

$$[w_0]_{\rm qcd,typeI} = 0.17235(22)(46)[51]\ \rm fm\ , \tag{S9}$$

where the first and second numbers in parentheses refer to the statistical and systematic uncertainties, respectively, and the number in square brackets is their quadrature sum, the total uncertainty. Note, that the definition of the QCD point is ambiguous, we therefore have indicated the choice of the QCD point in the subscript. For the E, F and G parameters we need to compute the electromagnetic derivatives of the various hadron masses. This has been done in our 2020 work and the fits yield the QED part:

$$[w_0]_{\rm qed,typeI} = 0.00010(3)(2)[3]\ \rm fm\ . \tag{S10}$$

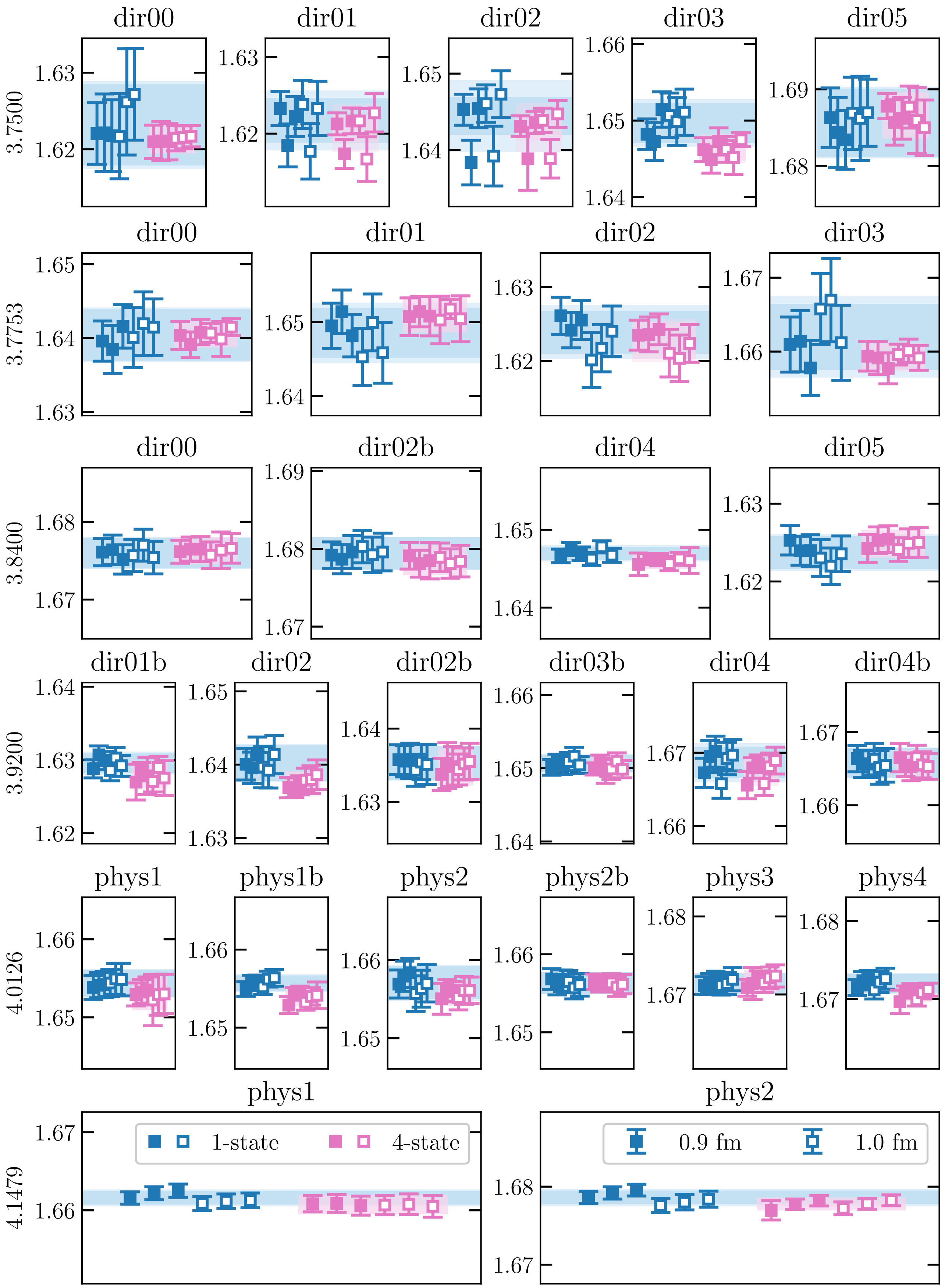

Figure S7: Results for the ground state Ω baryon mass on different ensembles, in units of GeV, using the lattice spacings from Table S1. Each ensemble has six data points with the blue colour, which correspond to correlated single exponential fits to the $\Omega_{\rm VI}$, $\Omega_{\rm XI}$ and $\Omega_{\rm Ba}$ propagators with fit starting point set at 0.9 fm, followed by the same fits starting at 1.0 fm. The earlier/later fit ranges are displayed with filled/open symbols. In addition we have another six data points with pink colour corresponding to fits with four exponentials. The blue/pink bands correspond to averages of the blue/pink points.

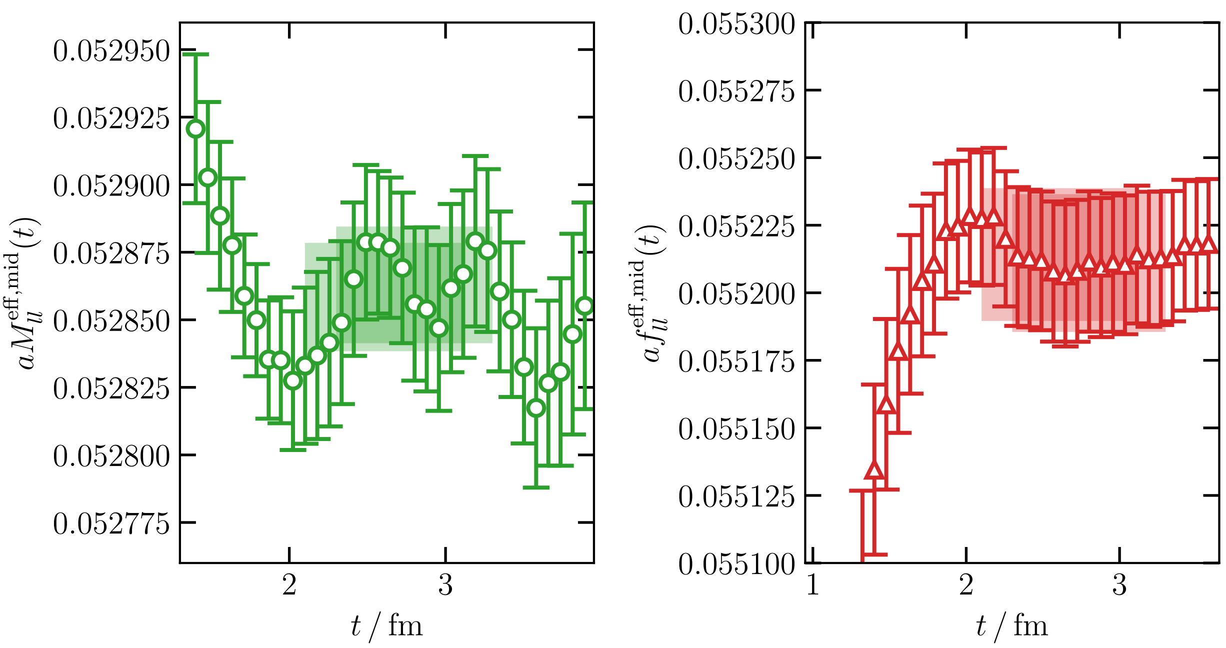

Figure S8: Midpoint effective pion mass and decay constant from one of the $\beta = 3.9200$ ensembles. The semi-transparent shaded bands show the plateaus, whose x-extents correspond to the two plateau regions (1.9 fm – 2.9 fm for the first, and 2.1 fm – 3.1 fm for the second) and y-extents, to the uncertainties on the associated fit results.

Finally, for the sum of the QCD and QED contribution we get

$$[w_0]_{\mathrm{qcd+qed}} = 0.17245(22)(46)[51]\ \mathrm{fm} \tag{S11}$$

which is a scheme independent quantity and the main result of this section. We will compare this value to other determinations in later sections of this paper.

S3 Scale setting with the pion decay rate

S3.1 Pion propagator measurements and fits

On each ensemble we obtain the pion mass and decay constant from the zero-momentum two-point function $G(t)$ of the flavour-non-singlet, pseudo-scalar density with pseudo-Goldstone staggered taste. We use random wall sources and point sinks for the measurements. On our finest lattice the integrated autocorrelation time is at most 10 trajectories for these propagators.

For sufficiently large $t \ll T$, excited states are exponentially suppressed and $G(t)$ approaches the single-state form

$$G(t) \simeq \frac{f_{ll}^2 M_{ll}^3}{4m_l} \frac{\cosh\left(M_{ll}\left(t - \frac{T}{2}\right)\right)}{e^{M_{ll}\frac{T}{2}}\left(1 - e^{-M_{ll}T}\right)}\ , \tag{S12}$$

where M_{ll} and f_{ll} are the mass and decay constant of the pion composed of degenerate, light quarks of bare mass m_l.

We define an effective mass which asymptotically takes the value M_{ll}. We consider two different definitions: the local effective mass

$$M_{ll}^{\mathrm{eff,\ loc}}(t) = \frac{1}{\Delta}\cosh^{-1}\frac{G(t+\Delta) + G(t-\Delta)}{2G(t)}\ , \tag{S13}$$

and the midpoint effective mass

$$M_{ll}^{\text{eff, mid}}(t) = -\frac{1}{2\Delta}\left[\cosh^{-1}\frac{G(t+\Delta)}{G(T/2)} - \cosh^{-1}\frac{G(t-\Delta)}{G(T/2)}\right] , \tag{S14}$$

and take the difference between the two as a source of systematic error. The parameter Δ is chosen independently for each ensemble such that Δ/a is even (to minimize the effects of the oscillating parity partners), and the physical value Δ is close to either 0.2 fm or 0.4 fm. The difference between the two choices for Δ is also taken as a systematic error.

We can also define an effective decay constant

$$f_{ll}^{\text{eff}}(t) = \sqrt{\frac{8m_l^2 G(t)}{M_{ll}^{\text{eff}}(t)^3}\frac{e^{M_{ll}^{\text{eff}}(t)\frac{T}{2}}(1-e^{-M_{ll}^{\text{eff}}(t)T})}{\cosh\left(M_{ll}^{\text{eff}}(t-\frac{T}{2})\right)}} , \tag{S15}$$

where we take M_{ll}^{eff} to be the same combination of loc or mid and Δ value used to extract the mass in a given analysis. Figure S8 shows an example of these effective mass and decay constants as they approach the asymptotic region.

In order to extract the asymptotic values of the effective mass and decay constant, we perform constant fits to their values in plateau regions chosen to be at sufficiently large Euclidean time that excited state effects are negligible. These plateau regions are selected with a method that is already used to optimize the plateau regions for the effective mass of the Ω baryon and makes use of the Q-value that is defined in Ref [82]. Based on this analysis, we select a plateau region of 1.9 fm – 2.9 fm. In order to account for possible systematic errors arising from any residual excited state contaminations, we also repeat the analysis with later plateaus at 2.1 fm – 3.1 fm and include the difference between the two as a systematic error. In Figure S8, we show the fits to these two plateau regions for an example effective mass and decay constant.

The plateau fits included here are all performed by minimizing the uncorrelated χ^2, which provides improved fit stability when fitting to many points [86]. The effects of correlations between the data points are taken into account by utilizing the Q-value [82], and by computing uncertainties in the fit values using the jackknife procedure.

S3.2 Finite-size effects

In our analysis, we correct the lattice data for finite-volume effects with continuum NNLO XPT [87], where for the low-energy constants we take the default values of the computer code in [88]. For our setup, we observe a good convergence of the chiral expansion: in a 6 fm box, the pion decay constant is smaller than its infinite-volume value by about 0.11% and 0.12% in NLO and NNLO, respectively. In our treatment of systematic uncertainties, we estimate possible higher-order effects by using the difference between NNLO and NLO results.

We also take into account the staggered artefacts of finite-size effects at NLO [89–92]. The staggered XPT expressions depend on hairpin parameters, $\delta_{KS}(A)$ and $\delta_{KS}(V)$, which are defined in Section S1.2. We use two different choices for these parameters in the analysis; their difference is treated as a systematic error. For the first choice, we take $\delta_{KS}(A) = -1.76$ and $\delta_{KS}(V) = 0.92$ from global fits to the pseudoscalar data of the Fermilab-MILC collaboration [73]. As a second choice, we use $\delta_{KS}(A) = -1.87$ and $\delta_{KS}(V) = 0.00$, which best fits our lattices with different volumes. On our coarsest lattice, the finite-size effect on the pion decay constant is increased from the above 0.11% to about 0.15% due to the staggered artefacts.

Finite-time effects are also included at NLO, and are found to be on the order of the difference between the NNLO and NLO finite-volume effects.

β	a [fm]	$L/a \times T/a$	tag	am_s	m_s/m_l	#confs
3.7000	0.1315	24×48	volume/24	0.057291	27.899	726
		48×64	volume/48	0.057291	27.899	300
3.7753	0.1116	28×56	dir00	0.047615	27.843	887
3.8400	0.0952	32×64	dir00	0.043194	28.500	1110
			dir02	0.043194	30.205	1072
			dir04	0.040750	28.007	1036
			dir05	0.039130	26.893	1035
0.7300	0.1120	56×84	phys2/56	0.06061	33.728	1305

Table S4: List of the ensembles used to measure the electromagnetic sea-sea contribution to the decay rate. The action of the first seven ensembles is `4stout`, that of the last ensemble is `4hex`. Columns are as in Table S1. In case of the `4hex` ensemble, our strange/light mass ratio is somewhat larger than physical, since there we tuned the light mass such that the taste-averaged pion mass takes approximately the physical value.

S3.3 Electromagnetic effects

In this work, we also consider the electromagnetic corrections to the pion decay rate. We restrict the analysis to the virtual photon exchange diagrams and use the point-like meson approximation for the real photon emission contribution. The structure-dependent corrections to the latter, for the muonic decay rate we use in this work, are found to be negligible [93, 94][3]. The virtual photon exchange contributions have been computed on the lattice in the electro-quenched approximation in [96]. The novelty of our work is that we compute the leading electro-unquenched contributions. Together, the contributions can be used to determine the muonic pion decay rate in lattice QCD.

There are two types of diagrams missing in [96], i.e. those where the photon is coupled to a sea-quark loop. In the first, the photon connects a sea quark to a valence quark or a lepton; in the second, the photon connects two sea quarks; we call them sea-valence and sea-sea contributions, respectively. The former is an $SU(3)$ flavour symmetry violating observable, and as such we expect it to be about 20% of the size of the latter. This flavour suppression of the sea-valence contributions compared to the sea-sea one is seen in several observables in our 2020 work [1]. There, we call these contributions F and G, and the smallness of F compared to G is apparent in the case of hadron masses in Figures 20 and 21 and, in the case of a_μ, in Figures 25 and 26. Also, one can estimate the individual electromagnetic contributions using partially quenched XPT at leading order $O(e^2p^2)$—see Refs. [97, 98] for the necessary expressions. The contribution of the interaction between a sea and a valence quark is free of low-energy constants, and we find that it is a -0.01% relative correction on the isospin-symmetric decay rate. The diagram with the interaction between a sea quark and a lepton is structure-dependent and only appears at $O(e^2p^4)$ in partially-quenched XPT. The flavour suppression and the XPT estimate justify neglecting the sea-valence contribution at our current level of precision. Therefore, in this work we compute only the sea-sea contribution.

The sea-sea contribution to an observable expectation value $\langle O \rangle$ is given by the second derivative with respect to the sea electric charge:

$$\partial_{02}\langle O\rangle = \left\langle [O_0 - \langle O_0\rangle_U] \left\langle \frac{\mathrm{dets}_2''}{\mathrm{dets}_0} \right\rangle_A \right\rangle_U , \tag{S16}$$

where we use the formulae from Section 5 of our 2020 work [1] and replace our old notation $[\ldots]''_{02}$ by $\partial_{02}\ldots$ for more clarity. Our definition of the partial derivative keeps the bare quark masses constant, and this type of "bare" derivative has to be renormalized (see later). We also consider the second derivative

[3] This statement holds also beyond the electro-quenched approximation, as shown recently in [95].

for the product of two or more expectation values, for example

$$\partial_{02}(\langle A\rangle\langle B\rangle) = \partial_{02}\langle A\rangle \cdot \langle B\rangle_0 + \langle A\rangle_0 \cdot \partial_{02}\langle B\rangle \ , \tag{S17}$$

where we use the fact that the first derivatives vanish at zero electromagnetic charge. We also use this notation for observables that are more general functions of expectation values,

$$\partial_{02} f(\langle A\rangle, \langle B\rangle) = \frac{\partial f(\langle A\rangle, \langle B\rangle)}{\partial\langle A\rangle} \cdot \partial_{02}\langle A\rangle + \frac{\partial f(\langle A\rangle, \langle B\rangle)}{\partial\langle B\rangle} \cdot \partial_{02}\langle B\rangle \ .$$

For the measurement of Equation (S16), we need the correlation, with respect to the gluon fields U, between the isospin-symmetric observable O_0 and the second derivative of the quark determinant $\langle \mathrm{dets}_2''/\mathrm{dets}_0\rangle_A$ that is averaged over photon fields A. The fermion determinants are measured on seven ensembles using the 4stout action and on one ensemble using the 4hex action. The measurement algorithm is given in Section 7 of [1], and the ensemble parameters can be found in Table S4.

The electromagnetic sea-sea contribution to the pion decay rate only enters in the quark part of the diagram; the leptons are unaffected. Therefore, the correlation of the isospin-symmetric pion decay constant f_{ll} with the derivative of the quark determinant gives the sea-sea contribution. For our computation, we also need the sea-sea contribution to the pion and kaon masses and to the gradient flow scale. These have already been computed in our 2020 work [1].

We investigate finite-volume effects of the sea-sea contribution by utilizing lattices with spatial extent around 3 fm and around 6 fm; see Table S4. In the analysis, we assume a volume dependence that is linear in the $1/L^2$ variable. This is justified by the fact that the sea-sea contribution probes the internal structure of the pion and the leading structure-dependent finite-volume effects are found to start at $1/L^2$ in the case of the leptonic decay rates of pseudoscalar mesons [99].

S3.4 Determination of w_0 using the pion decay rate - formulae

Our objective is to determine the gradient-flow scale w_0 from lattice QCD to leading order in isospin-breaking corrections, using the muonic decay rate of charged pions as input. In this subsection, we provide the necessary formulae. We start by defining F_{ud}, the square root of the decay rate Γ of the pion, excluding V_{ud} and kinematical factors:

$$F_{ud}^2 = \Gamma(\pi \to \mu\bar{\nu}_\mu[\gamma]) \left[\tfrac{G_F^2}{8\pi}|V_{ud}|^2 M_{ud} m_\mu^2 \left(1 - m_\mu^2/M_{ud}^2\right)\right]^{-1} \ . \tag{S18}$$

Here, μ denotes the muon, m_μ its mass, $\bar{\nu}_\mu$ the corresponding antineutrino, G_F the Fermi constant and M_{ud} the pion mass. This combination was introduced in [96]. It is an observable defined in full QCD plus QED and can be measured in experiment, assuming a value for $|V_{ud}|$. We denote its physical value by $[F_{ud}]_{\mathrm{qcd+qed}}$ for which we obtain

$$[F_{ud}]_{\mathrm{qcd+qed}} = 131.711(45)\ \mathrm{MeV} \ , \tag{S19}$$

using results from [85], in particular $V_{ud} = 0.97367(32)$[4] and $\Gamma = 3.8408(7) \times 10^7/\mathrm{s}$. In pure QCD, this observable corresponds to the usual pion decay constant, denoted here $[F_{ud}]_{\mathrm{qcd}}$. Its value depends on the scheme that defines the separation between the QCD and QED contributions in Equation (S18), whereas $[F_{ud}]_{\mathrm{qcd+qed}}$ does not. In any scheme, we can decompose an observable O into QCD and QED parts, the

[4]There is some tension between the Particle Data Group value $V_{ud} = 0.97367(32)$ coming from nuclear β-decays and the FLAG value $V_{ud} = 0.97439(14)$ [73, 100–106] coming from the decay rate ratio of kaons and pions, assuming the unitarity of the CKM matrix and using lattice computations of the f_K/f_π ratio with isospin-breaking correction taken from chiral perturbation theory. We choose to work with the PDG value here, since it relies on fewer assumptions. We discuss the impact of the uncertainty of V_{ud} on our scale determination in Equation (S34).

latter even into valence-valence, sea-valence, and sea-sea contributions:

$$[O]_{\rm qcd+qed} = [O]_{\rm qcd} + [O]_{\rm qed,vv} + [O]_{\rm qed,vs} + [O]_{\rm qed,ss} \ .$$

We adopt the valence-valence QED contribution from the lattice computation of [96], which employs the scheme of Gasser, Rusetsky and Scimemi (GRS) [107]. This scheme is defined in such a way that the strong coupling α_s and the quark masses m_u, m_d and m_s, renormalized at some fixed scale Λ, remain constant as the electromagnetic coupling is turned on or off. In particular, the authors of [96] determine[5] that the valence-valence QED contribution relative to the strong one is

$$[F_{ud}]_{\rm qed,vv}/[F_{ud}]_{\rm qcd} = (1+\delta R_\pi)^{1/2} - 1 \quad \text{with} \quad \delta R_\pi = 0.0150(18) \ . \tag{S20}$$

To reconstruct the full decay rate, the other QED contributions must also be computed in the GRS scheme. This can be achieved without having to match renormalized quark masses in QCD and in QCD plus QED at a fixed renormalization scale. It is sufficient to have the decomposition of the pion and kaon masses in the GRS scheme. The authors of [96] provide these. For the QCD contributions they obtain [108]:

$$[M_{ud}]_{\rm qcd++} = 135.0(2)\ \text{MeV} \ , \tag{S21}$$

$$[\tfrac{1}{2}(M_{us}+M_{ds})]_{\rm qcd++} = 494.6(1)\ \text{MeV} \ . \tag{S22}$$

A subtle point arises here. This QCD++ component is obtained by subtracting the valence-valence QED contribution from the physical QCD plus QED values. Thus, the QCD component in Ref. [96] includes not only pure QCD but also sea-valence and sea-sea QED effects, as signaled by the label QCD++. For a generic observable O we have

$$[O]_{\rm qcd++} \equiv [O]_{\rm qcd} + [O]_{\rm qed,vs} + [O]_{\rm qed,ss} \ .$$

Using δR_π and the full QCD plus QED value from above, the pion decay constant in this scheme can be written

$$[F_{ud}]_{\rm qcd++} = [F_{ud}]_{\rm qcd+qed}/(1+\delta R_\pi)^{1/2} = 130.73(12)\ \text{MeV} \ . \tag{S23}$$

As we will see soon, we specifically require the QCD++ —not only the QCD—component of these quantities.

We also note that, in Ref. [96], the authors used the QCD pion decay constant from Equation (S23) to set the scale for their calculations of the QCD++ components of the masses in Equations (S21) and (S22). An alternative scale choice—differing from the QCD pion decay constant by isospin-breaking corrections—would only impact the results in Equations (S21) and (S22) at the level of neglected $O(e^4)$ terms. This is because the measurement specifically targeted the valence QED component of these masses, which is an $O(e^2)$ quantity.

We now proceed to determine the product $w_0 F_{ud}$ in full QCD plus QED. We parameterize its quark-mass and electric-charge dependence as

$$w_0 F_{ud} = A \ + B\ F_{ud}^{-2} M_{ud}^2 + C\ F_{ud}^{-2}(M_{us}^2 + M_{ds}^2 - M_{ud}^2)/2 + E\ e_v^2 + F\ e_v e_s + G\ e_s^2 \ , \tag{S24}$$

where e_v and e_s denote the unit electric charges associated with the valence and sea quarks, and where we neglect all $O(e^4)$ and other NLO isospin-breaking corrections, as we do throughout this section and paper. The quantity w_0, as well as all masses and decay constants appearing in this expression, are understood to be those obtained on the lattice in the full QCD plus QED theory, for a variety of quark masses and electric charges. Accordingly, these quantities are not labeled with the subscript $\rm qcd+qed$, which we

[5] Ref. [96] gives $\delta R_\pi = 0.0153(19)$, which value includes a XPT estimate of sea effects of 0.003. Here, we need a value with the valence contribution only, therefore we use the value quoted in the text.

reserve for their physical values, e.g. in Equation (S19). We note that F_{ud} on the left- and right-hand sides of this equation refer to the same quantity.

In Equation (S24), the quantity w_0F_{ud} is treated as a function of the independent variables $F_{ud}^{-2}M_{ud}^2$, $F_{ud}^{-2}(M_{us}^2 + M_{ds}^2 - M_{ud}^2)/2$, e_v and e_s. For instance, G is the second, partial derivative of w_0F_{ud} with respect to e_s, at fixed values of the other variables. More generally, the parameters $A, \cdots, G$, which are not those for w_0M_Ω in Equation (S8), can be obtained by fitting Equation (S24) to the dependence of w_0F_{ud} on the independent variables for varying quark masses and charges around their physical values. All dependence on the independent variables is explicit in Equation (S24)[6].

The observables $F_{ud}^{-2}M_{ud}^2$ and $F_{ud}^{-2}(M_{us}^2+M_{ds}^2-M_{ud}^2)/2$ are themselves functions of QCD plus QED's bare parameters, as is w_0F_{ud}. After appropriate renormalization, they are finite. Because we are working to first order in QED, the bare parameters e_v and e_s are equal to their renormalized values, and are also finite. Thus, Equation (S24) relates independent, finite observables in the full theory, implying that the parameters $A, \cdots, G$ are finite and independent of any scheme used for separating these observables into pure QCD and QED contributions. The remainder of this subsection explains how we compute these parameters, which are needed to determine the physical value of w_0F_{ud}.

We begin with A, B and C. Since our target observable is symmetric under $u \leftrightarrow d$ replacement, and we work to leading order in isospin breaking, no term is required for the strong-isospin breaking (the isospin-breaking effect from the up-down quark mass difference). Now, by definition, QCD plus QED observables are equal to their pure QCD counterparts, up to order-charge-squared corrections, in any separation scheme. Thus, setting $e_v = e_s = 0$, Equation (S24) reduces to

$$w_0 f_{ll} = A + B\ f_{ll}^{-2}M_{ll}^2 + C\ f_{ll}^{-2}(M_{ls}^2 - \tfrac{1}{2}M_{ll}^2)\ , \tag{S25}$$

where f_{ll} and M_{ll} are the pion decay constant and mass, respectively, computed in pure isospin-symmetric QCD. This means that A, B and C can be obtained with a pure isospin-symmetric QCD calculation, by fitting the dependence of w_0f_{ll} on $f_{ll}^{-2}M_{ll}^2$ and $f_{ll}^{-2}(M_{ls}^2 - \frac{1}{2}M_{ll}^2)$ to that equation.

We now turn to the electromagnetic coefficients E, F, and G. As discussed above, the valence-valence coefficient E has already been determined in [96]. To complete the calculation of $[w_0F_{ud}]_{\text{qcd+qed}}$, we must compute the sea-valence and sea-sea electromagnetic coefficients F and G. We begin with the sea-sea electromagnetic coefficient G. We compute it via the bare, second, partial derivative of Equation (S24) with respect to e_s, defined in Equation (S16), and with the rule for second derivatives of products of observables in Equation (S17). Here, the derivatives are taken with e_v and the bare parameters of pure QCD held fixed. Thus, G is obtained by fitting the sea-electric-charge derivative

$$G = \partial_{02}\left(w_0F_{ud} - B\ F_{ud}^{-2}M_{ud}^2 - C\ F_{ud}^{-2}(M_{us}^2 + M_{ds}^2 - M_{ud}^2)/2\right) \tag{S26}$$

to a constant, where the B and C parameters are taken from the isospin-symmetric fit. Note that the terms proportional to B and C in Equation (S26) effectively remove the divergences in the bare derivative $\partial_{02}(w_0F_{ud})$, which arise from the bare QED corrections to the quark masses: G is a physical, renormalized quantity[7]. For this fit, we need the measurements of f_{ll} and the derivative of the quark determinant, as described in Equation (S16). The sea-valence term F can be determined from sea-valence derivatives. These are flavor-$SU(3)$-suppressed and expected to be even smaller than the 0.1% contribution from sea-sea effects. Thus, for the level of precision required here, we can set $F = 0$. We leave its computation for future work.

We now have all the ingredients needed to determine the physical value of w_0F_{ud}. We decompose it

[6] An additional term, like $e_s^2\partial_{02}(F_{ud}^{-2}M_{ud}^2)$, is not needed in Equation (S24). In that Equation $F_{ud}^{-2}M_{ud}^2$ and e_s^2 are treated as independent variables, which means that $F_{ud}^{-2}M_{ud}^2$ can be adjusted independently of e_s^2. In the following step we change the set of independent variables, replacing $F_{ud}^{-2}M_{ud}^2$ and $F_{ud}^{-2}(M_{us}^2 + M_{ds}^2 - M_{ud}^2)/2$ by the bare light and strange quark masses, and $F_{ud}^{-2}M_{ud}^2$ becomes a function of the bare parameters, ie. also of e_s^2.

[7] The B coefficient itself is a finite, renormalized quantity. The divergence related to the QED correction on the light quark mass appears in $\partial_{02}(F_{ud}^{-2}M_{ud}^2)$, that is multiplied by B. This divergence together with the one coming from the C-term cancel the divergence in $\partial_{02}(w_0F_{ud})$ to give a finite G.

as

$$[w_0F_{ud}]_{\rm qcd+qed} = [w_0F_{ud}]_{\rm qcd++} + [w_0F_{ud}]_{\rm qed,vv} \ . \tag{S27}$$

The QCD++ contribution is obtained via

$$[w_0F_{ud}]_{\rm qcd++} = A + B\ [F_{ud}^{-2}M_{ud}^2]_{\rm qcd++} + C\ [F_{ud}^{-2}(M_{us}^2+M_{ds}^2-M_{ud}^2)/2]_{\rm qcd++} + Fe^2 + Ge^2\ , \tag{S28}$$

where the A, B, C, F, G parameters are determined as described above, and the QCD++ values of the pion, kaon masses and decay constant are taken from Equations (S21), (S22) and (S23). For the valence-valence QED part, we use Equation (S20). It can be rewritten as

$$\frac{[w_0F_{ud}]_{\rm qed,vv}}{[w_0F_{ud}]_{\rm qcd}} = (1+\delta R_\pi)^{1/2} - 1\ , \tag{S29}$$

because w_0 is defined in terms of purely gluonic quantities and is therefore free of valence-valence QED contributions.

Adding the QCD++ and valence-valence QED contributions, we get the physical value

$$[w_0F_{ud}]_{\rm qcd+qed} = [w_0F_{ud}]_{\rm qcd++} + ((1+\delta R_\pi)^{1/2}-1)[w_0F_{ud}]_{\rm qcd} \approx [w_0F_{ud}]_{\rm qcd++}(1+\delta R_\pi)^{1/2} \tag{S30}$$

where we drop terms that are higher order in e^2. Finally, dividing by the physical value of F_{ud}, and using Equation (S23), we arrive at the physical value of w_0 itself

$$[w_0]_{\rm qcd+qed} = [w_0F_{ud}]_{\rm qcd++}/[F_{ud}]_{\rm qcd++}\ , \tag{S31}$$

which is the main result of this subsection.

The parameterization in Equation (S24) can also be used to present results in a different scheme, e.g. the Edinburgh consensus scheme, which has now also been adopted by FLAG [100]. It is defined by

$$\begin{aligned}[][M_{ud}]_{\rm qcd,FLAG} &= 135.0\ \rm MeV\\ [\tfrac{1}{2}(M_{us}+M_{ds})]_{\rm qcd,FLAG} &= 494.6\ \rm MeV\\ [F_{ud}]_{\rm qcd,FLAG} &= 130.5\ \rm MeV\end{aligned}$$

Substituting these values into Equation (S24) with E, F and G set to zero and dividing by $[F_{ud}]_{\rm qcd,FLAG}$ gives us $[w_0]_{\rm qcd,FLAG}$. The QED part in this scheme, $[w_0]_{\rm qed,FLAG}$, can be computed by subtracting the QCD part from the full physical value.

S3.5 Determination of w_0 using the pion decay rate - results

Here we discuss in detail the fit procedure, which gives $[w_0]_{\rm qcd+qed}$. We start with the isospin-symmetric fits of Equation (S25), then we explain the fits to the sea-sea contribution, Equation (S26).

The isospin-symmetric fits determine the parameters A, B and C. For the A parameter we allow for polynomials of a^2 at most cubic order. Additionally, we allow for modifying the a^2 term by an $\alpha_s^\gamma(a)$ factor, where $\alpha_s(a)$ is the strong coupling at the scale of the lattice spacing and the exponent γ is taken from the set $\{0.0, 0.5, 1.0, 1.5, 2.0, 2.5\}$. With this variation, we address the uncertainties related to the *a priori* unknown logarithmic corrections of the a^2 dependence (see Reference [109] and the discussion in Section S5.1). The B and C parameters correspond to the mass dependences in the light and strange quarks. Here we allow for a polynomial that is either constant or linear in a^2. We drop the coarsest lattice spacing, $\beta = 3.7000$, entirely from the analysis. The fit results are shown in Table S5.

In these fits we use two different definitions for the gradient-flow: the first is the original prescription of Lüscher [110], for the second we take the Zeuthen-flow [111], which is free from $O(a^2)$ artefacts. These effects are classical, and therefore are not modified by logarithmic corrections[8]. In the continuum

[8]We thank D. Nogradi and A. Ramos for discussions on lattice artefacts of gradient flows.

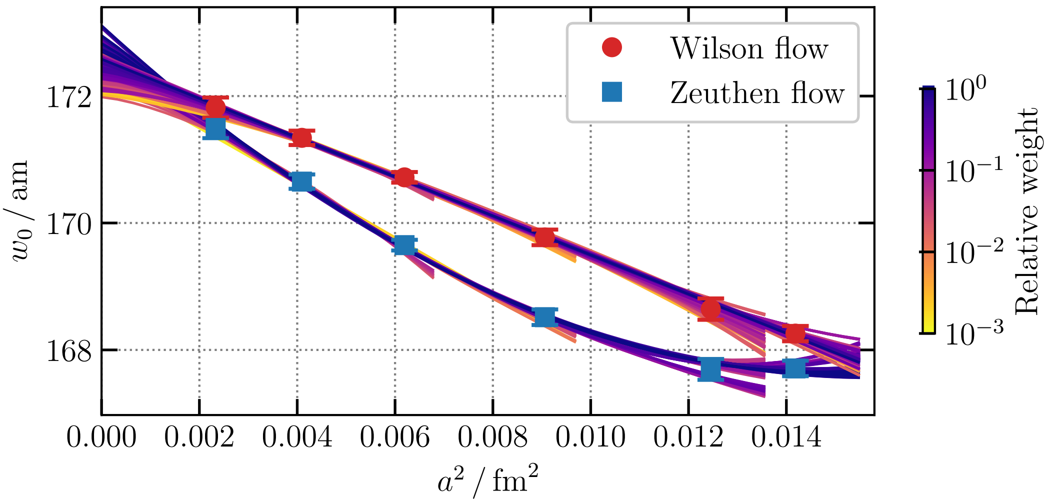

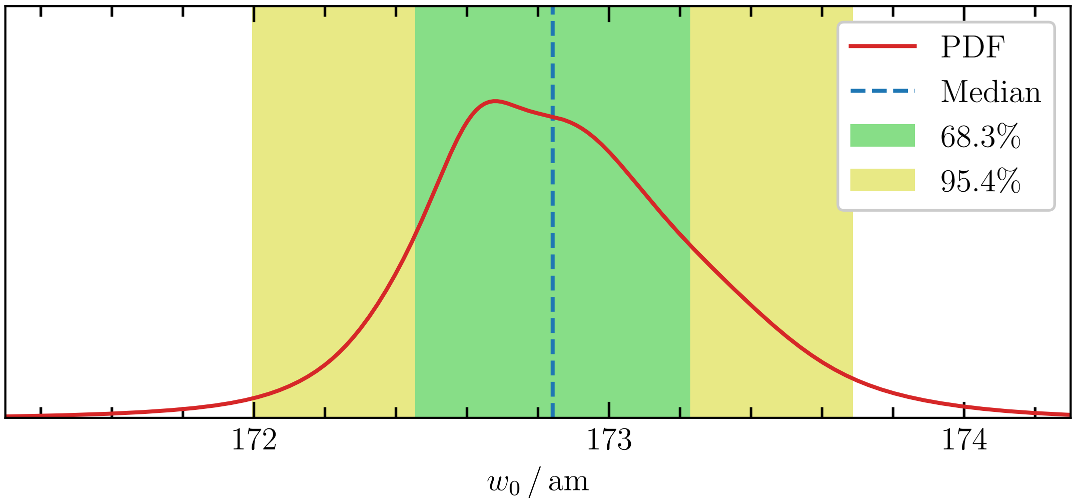

Number of fits	12672	
Fits with $P > 0.1$	74%	
Median	172.84	
Total error	0.42	0.24%
Statistical error	0.34	0.20%
Systematic error	0.25	0.14%
Pseudoscalar fits	0.15	0.09%
Finite volume XPT	0.03	0.02%
Type of flow (Ze/Wi)	0.14	0.08%
Lattice spacing cut	0.04	0.02%
Fit polynomial order	0.11	0.06%
Log corrections γ	0.07	0.04%

Table S5: Gradient-flow scale w_0 in attometers using the pion decay constant as input. Results are given in the isospin-symmetric point of the FLAG scheme and in the infinite volume limit. The continuum extrapolations are shown for two definitions of the gradient flow, Wilson and Zeuthen. More details on the plot conventions can be found in the first part of Section S6.

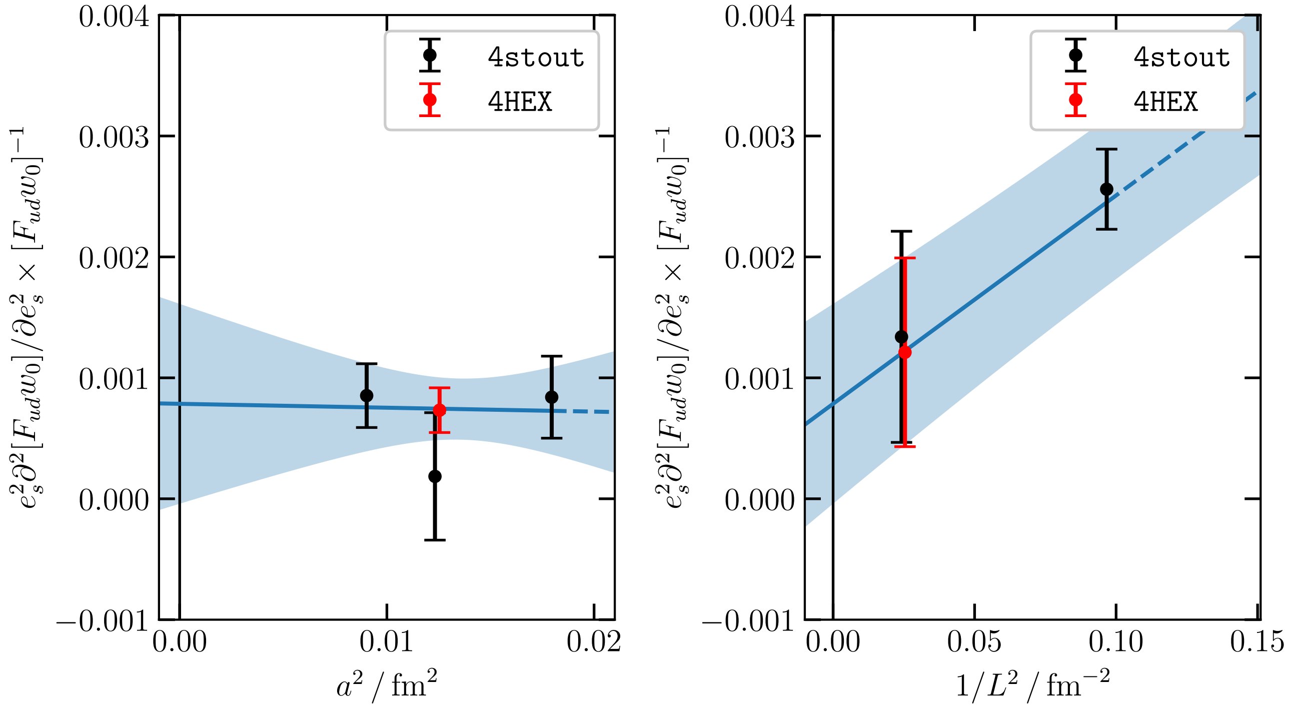

Figure S9: Renormalized second derivative of $[w_0 F_{ud}]$ with respect to the sea electromagnetic charge, which is called G parameter in the text. We multiply by the physical charge squared and normalize by the isospin symmetric value of $[w_0 F_{ud}]$, such that we can interpret the quantity as a relative correction. The left panel shows the continuum, the right panel the infinite volume extrapolation.

extrapolation plots, we show results with both discretizations. The difference between the results of these two flows becomes part of our systematic error.

To facilitate the comparison with other lattice collaborations, we present the isospin-symmetric results in the FLAG scheme, as defined above. The continuum-extrapolated result at the isospin-symmetric point of FLAG is

$$[w_0]_{\text{qcd,FLAG}} = 0.17284(34)(25)[42] \text{ fm} . \tag{S32}$$

We also show a histogram and error budget for these isospin-symmetric fits. The largest source of systematic error comes from the variation in the pseudoscalar fit range, closely followed by the variations in the type of the gradient flow (Wilson vs Zeuthen) and in the functional form used for the continuum extrapolation.

We also investigate the impact of the new $a = 0.048$ fm ensembles. Without these ensembles, we obtain $0.17279(37)(40)[54]$ fm, implying an uncertainty reduction of $1 - 0.00042/0.00054 = 22\%$. This improvement comes mainly from a better control of the uncertainties related to the fit form and from a reduction of the difference between the results obtained from the Wilson and Zeuthen flows: the finest lattice spacing improves the reliability of the continuum extrapolation.

For the sea-sea contribution, we have to determine the G parameter of Equation (S26). Here we assume a linear dependence in a^2 for the `4stout` ensembles; for the single `4hex` ensemble we either assume the same artefacts as for the `4stout` or no artefacts at all. The difference of these two cases becomes part of the systematic error. We also assume a volume dependence, linear in $1/L^2$. Note, a $1/L$ behaviour is excluded, since it must be structure independent. Some of these fits are shown in Figure S9. The left panel shows the continuum extrapolation, the right panel the infinite-volume extrapolation. We plot the G parameter multiplied by the physical value of the electric charge squared and divided by the isospin-symmetric value of $w_0 F_{ud}$. We can read off from the plot that the sea-sea contribution in the continuum is about 0.1% of the isospin-symmetric value, with an error of $\pm 0.1\%$. From the $SU(3)$ flavour

suppression, we estimate the sea-valence contribution to be about 20% of the sea-sea contribution, i.e. about 0.02% of the isospin-symmetric value. This is consistent with our partially-quenched XPT estimate, which is -0.01%. It is therefore reasonable to assume that the sea-valence contribution is negligible at the current level of precision.

We can now put together the results of the above fits as required by Equation (S31) to get

$$[w_0]_{\text{qcd+qed}} = 0.17264(36)(33)[48]\ \text{fm}\ , \tag{S33}$$

which is our final result for the gradient flow scale in QCD plus QED. It has a relative precision of 2.8 per mille. The first error is statistical, the second is systematic, the third is these two added in quadrature. The systematic error can be split up as

$$(33) = (27)_{\text{qcd}}(18)_{\text{qvv}}(9)_{\text{qss}}(6)_{V_{ud}}(3)_{\text{exp}} \tag{S34}$$

where the first error contains the systematic error of the isospin-symmetric fits, the second arises from the uncertainty of the valence-valence electromagnetic effects from the work of [96], the third is the uncertainty of the electromagnetic sea effects, the fourth comes from the uncertainty of V_{ud} and the last from the uncertainty of the other experimental inputs, see Equation (S19). Finally, we can also give the electromagnetic part of w_0 in the FLAG scheme, for which we subtract Equation (S32) from (S33) to get

$$[w_0]_{\text{qed,FLAG}} = -0.00027(10)(23)[25]\ \text{fm}\ . \tag{S35}$$

While the QCD and QED parts are scheme dependent, their sum can be directly compared to our gradient flow scale determination using the Omega baryon as input. This is done in Section S4.

S4 Physical point and isospin decomposition

In our computations, we parameterize the quark-mass and electromagnetic-coupling dependence of the observables using the gradient-flow-based w_0 scale and the connected up, down, and strange pseudoscalar meson masses M_{uu}, M_{dd} and M_{ss}. These masses are computed by taking into account only the quark-connected contributions to the corresponding two-point functions, as in Ref. [112], and are rigorously defined in a partially-quenched theory. Concretely, the parameterization of an observable Y is given as

$$Y = A + B\ w_0^2\hat{M}^2 + C\ w_0^2 M_{ss}^2 + D\ w_0^2\Delta M^2 + E\ e_v^2 + F\ e_v e_s + G\ e_s^2\ , \tag{S36}$$

where e_v and e_s are the electromagnetic couplings of the valence and sea quarks. We use $\hat{M}$ to denote the average $\hat{M}^2 \equiv \frac{1}{2}(M_{uu}^2 + M_{dd}^2)$ and ΔM^2 to denote the difference $\Delta M^2 \equiv M_{dd}^2 - M_{uu}^2$, which is a measure of strong isospin breaking. We called these kinds of parameterizations "Type-II" fits in our 2020 work. In this section, we first determine the physical point, then we decompose the Y observable into different isospin components.

S4.1 Physical point

Our basis observables, w_0, $\hat{M}$, M_{ss}, ΔM, cannot be measured experimentally. Still, they have a well-defined continuum limit so that a physical value can be associated with each of them. These values can be computed in the full theory, i.e. QCD plus QED, where four experimentally measured hadron masses are set to their physical values.

According to leading-order partially-quenched chiral perturbation theory coupled to photons [98], the physical value of $\hat{M}$ is equal to the neutral pion mass, i.e.

$$[\hat{M}]_{\text{qcd+qed}} = 134.9768(5)\ \text{MeV}. \tag{S37}$$

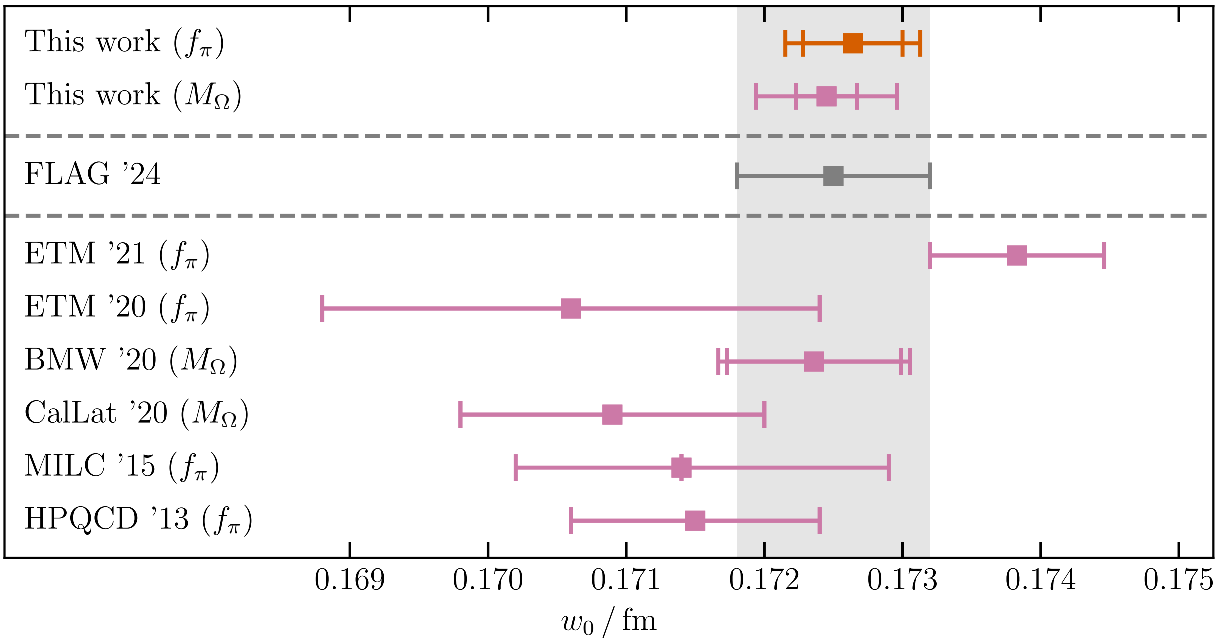

Figure S10: Comparison of recent determinations [1, 102, 106, 113–115] of the gradient-flow scale w_0. The upper two values correspond to our results from Sections S2 and S3 of this work, using the Omega-baryon mass and the pion leptonic-decay rate as input. The grey band shows the latest FLAG average [100].

This equality is valid up to next-to-leading order effects in isospin breaking, which we neglect in this work. For the physical values of the remaining meson masses, we use the analysis from 2020 [1]. For completeness, we give them here:

$$\begin{aligned} [\Delta M^2]_{\text{qcd+qed}} &= 13170(320)(270)[420]\ \text{MeV}^2, \\ [M_{ss}]_{\text{qcd+qed}} &= 689.89(28)(40)[49]\ \text{MeV}, \end{aligned} \tag{S38}$$

where the first error is statistical, the second is systematic, and the third is the total.

Finally, we turn to the gradient-flow scale w_0. Figure S10 shows determinations from various lattice collaborations. As input, either the Omega baryon mass or the pion decay constant is used. The band shows the latest average from FLAG [1, 100, 102, 106, 113, 114]. In our 2020 work and also in a previous version of this paper, we used the Omega baryon mass as input to compute the physical value $[w_0]_{\text{qcd+qed}}$. Our latest determination using this approach (presented in Section S2) is indicated by "This work (M_Ω)" on the plot. As explained in Section S2, all computations based on the Omega baryon have a systematic error of unknown size, arising from multi-hadron excited states in baryon propagators. As a consequence, we replace the Omega baryon with the pion leptonic-decay rate as the input quantity of the scale setting procedure, see Section S3. The determination is the first that takes both valence and sea quark electromagnetic effects into account. It gives the physical value of the gradient flow scale of Equation (S33). This value is labelled by "This work (f_π)" in the comparison plot. It agrees nicely with our determination using the mass of the Omega baryon as input. We use this pion-decay-rate w_0 value in our computations of the hadronic vacuum polarization. That is, on each ensemble the lattice spacing a is defined as the physical value of $[w_0]_{\text{qcd+qed}}$ from Equation (S33) divided by the value of $[w_0/a]$ measured on the given ensemble.

To obtain the physical value of some observable Y, we implement the following procedure:

1. The parameters $A, \ldots, G$ in Equation (S36) are determined from the fitting procedure described in Section S5.

2. The physical values of the basis observables above are substituted into the equation to obtain the physical result.

We incorporate the errors in the basis observables and also their correlations into our analyses by a stochastic sampling of their respective distributions. The procedure is described in detail in Section S5. This approach respects the statistical correlations, while most systematic ingredients are treated as uncorrelated. We justify this choice by noting that our basis observables have very different lattice artefacts compared to the ones with which they are fitted.

S4.2 Isospin decomposition

We can decompose an observable Y into isospin-symmetric and isospin-breaking contributions. This decomposition is, of course, not unique and depends on how the isospin-symmetric theory is defined. Thus, pure QCD results have a scheme ambiguity, which one has to keep in mind when performing comparisons. We have already seen several possible choices: the FLAG, GRS and "Type-I" schemes in Sections S2 and S3.

In our 2020 work, we put forward a scheme that is based on the parameterization in Equation (S36). In that scheme, which we call "BMW" scheme, we define the physical values of the basis observables, w_0, M_{uu}, M_{dd} and M_{ss}, to be the same in QCD and in the full theory, ie. QCD plus QED. In particular, the electromagnetic part of the gradient-flow scale is zero by definition, $[w_0]_{\mathrm{qed,BMW}} = 0$, as opposed to other schemes, where it can attain a non-zero value–see Equations (S10) for the "Type-I" or Equation (S35) for the FLAG scheme. The physical value of the observable Y can be decomposed into isospin-symmetric, strong-isospin-breaking and electromagnetic parts as

$$[Y]_{\mathrm{qcd+qed}} = [Y]_{\mathrm{iso,BMW}} + [Y]_{\mathrm{sib,BMW}} + [Y]_{\mathrm{qed,BMW}} \tag{S39}$$

with

$$\begin{aligned} [Y]_{\mathrm{iso,BMW}} &= A + B\ [w_0^2 \hat{M}^2]_{\mathrm{qcd+qed}} + C\ [w_0^2 M_{ss}^2]_{\mathrm{qcd+qed}} \\ [Y]_{\mathrm{sib,BMW}} &= D\ [w_0^2 \Delta M^2]_{\mathrm{qcd+qed}} \\ [Y]_{\mathrm{qed,BMW}} &= E\ e^2 + F\ e^2 + G\ e^2\ . \end{aligned} \tag{S40}$$

The sum of the first two gives the QCD contribution $[Y]_{\mathrm{qcd,BMW}}$. In this work, the isospin contributions are given in the "BMW" scheme, unless indicated otherwise.

S4.3 Kaon mass decomposition in different schemes

The isospin-symmetric point depends on the observables that define it, an effect commonly referred to as scheme dependence. A similar scheme to ours was put forward already in Ref. [117], though the physical values of the defining observables were not computed there. A scheme based on the Lagrangian parameters of the theory, which keeps renormalized quark masses and the strong coupling fixed while turning on the electromagnetic interaction, was proposed by Gasser, Rusetsky and Scimemi [107]. This scheme is called the GRS scheme in the literature and was implemented on the lattice in Refs. [96, 108].

To compare our scheme with others, we decompose the neutral and charged kaon masses into isospin components. For this purpose, we fit the kaon masses to Equation (S36), and use Equations (S39) and (S40) to compute the various components. The analysis includes about ten thousand fits to estimate systematics related to, e.g. continuum extrapolation and choice of the mass fit range. The decomposition we obtain is

$$[M_{us}]_{\mathrm{iso}} = 494.55[31]\ \mathrm{MeV} \qquad \begin{aligned} [M_{ds}]_{\mathrm{sib}} &= +\,2.98[14]\ \mathrm{MeV} \\ [M_{us}]_{\mathrm{sib}} &= -\,3.13[17]\ \mathrm{MeV} \end{aligned} \qquad \begin{aligned} [M_{ds}]_{\mathrm{qed}} &= 0.05[7]\ \mathrm{MeV} \\ [M_{us}]_{\mathrm{qed}} &= 2.25[8]\ \mathrm{MeV} \end{aligned} \tag{S41}$$

where the uncertainties given are the statistical and systematic errors added in quadrature. Figure S11 shows a comparison with the same decomposition in the GRS scheme. The values are taken from Ref. [108].

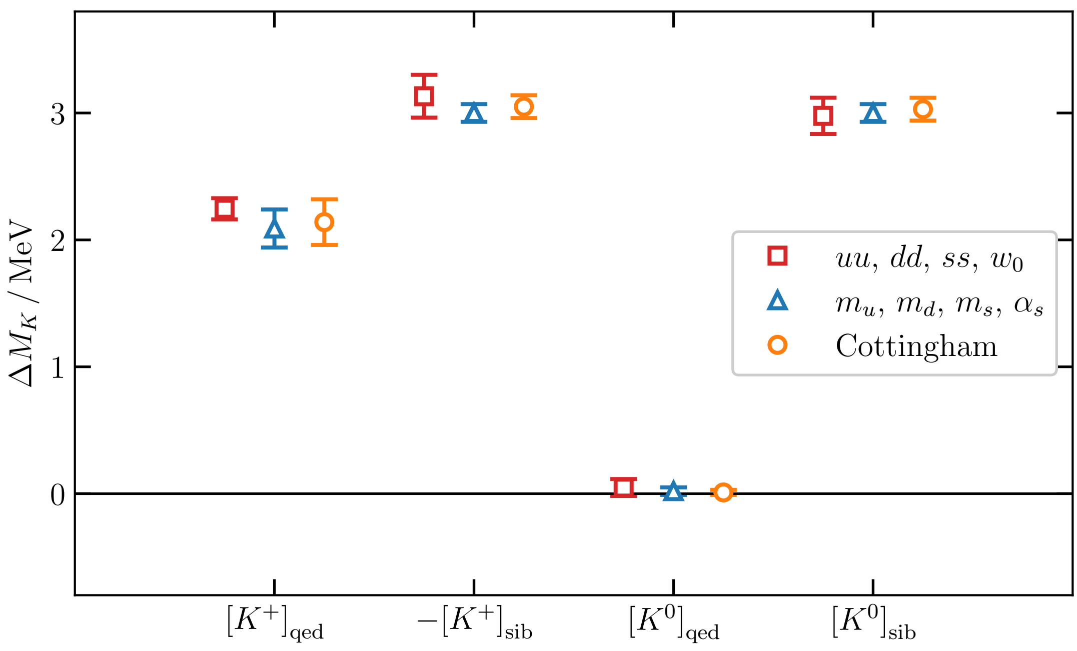

Figure S11: Decomposition of the neutral and charged kaon masses. Red squares stand for the BMW scheme, which is based on the observables $\{M_{uu}, M_{dd}, M_{ss}, w_0\}$; blue triangles for the GRS scheme, based on quark masses and strong coupling constant taken from [96]. Orange circles are obtained by computing the electromagnetic self-energies using the Cottingham-formula [116].

Note that they are computed in the electro-quenched approximation, whereas our results also include sea-valence electromagnetic effects. Sea-sea electromagnetic effects are absent in mass isospin splittings. The neglected sea-valence effects in Ref. [108] should be small, due to $SU(3)$ flavour suppression. It is possible to compute the electromagnetic self-energies of hadrons using the Cottingham formula [118]. For kaons, this was done in Ref. [116]. We also show these results in the Figure. We find good agreement between the different decompositions.

S5 Analysis procedure

S5.1 Fit functions

To obtain the physical isospin-symmetric values of different hadronic vacuum polarization windows we perform global fits to the lattice spacing and quark mass dependence of these observables.

Naïvely, we expect the leading discretization errors to scale like a^2. In general, however, this is modified by the anomalous dimensions of operators in the Symanzik effective theory, to become a sum of terms proportional to $a^2\alpha_s^\gamma(a)$ with different values of the exponent γ [109], where $\alpha_s(a)$ is the strong coupling at the scale of the lattice spacing. The set of values of γ is not yet known for our lattice action and observables. (For the case of unrooted staggered fermions with different number of flavours results have been presented in [119].) Also the prefactors appearing in these terms can be very different. Getting a stable fit with multiple a^2 terms having different γ's is difficult with the currently available lattice spacings. Instead, we perform fits with a single $a^2\alpha_s^\gamma(a)$ term at a time and vary the γ subsequently to address the uncertainty related to these logarithmic corrections[9]. In particular, we use the following function

$$A(a) = A_0 + A_2 \cdot (a/w_0)^2 \alpha_s^\gamma(a) + A_4 \cdot (a/w_0)^4 + A_6 \cdot (a/w_0)^6 \tag{S42}$$

[9] We thank R. Sommer for a discussion on possible fit forms.

to parameterize the lattice artefacts, where we allow the variation of γ. Here A_4 and A_6 describe higher order corrections in the lattice spacing.

For the quark mass dependence, the variables

$$X_l = \hat{M}^2 w_0^2 - \left[\hat{M}^2 w_0^2\right]_{\text{qcd+qed}} \quad \text{and} \quad X_s = M_{ss}^2 w_0^2 - \left[M_{ss}^2 w_0^2\right]_{\text{qcd+qed}} \tag{S43}$$

describe the deviation from the physical light and strange-quark mass respectively. Here "qcd+qed" denotes the physical values given in Equations (S37), (S38) and (S33). In the case of isospin-symmetric fits $\hat{M}$ is given by M_{ll}. For w_0/a we take the Zeuthen version of the gradient flow. In our global fit we consider terms linear in the X_l and X_s variables. No higher orders are needed, since we work close to the physical point. We allow these terms to depend on the lattice spacing, so their coefficients become polynomials of $(a/w_0)^2$, denoted by $B(a)$ and $C(a)$ in the following.

Putting all this together, the global fit function is

$$Y = A(a) + B(a)X_l + C(a)X_s \ , \tag{S44}$$

where Y is one of our dimensionless target observables. We consider different polynomial orders for A, B, and C, as well as omitting up to three of the coarsest lattice spacings (out of a total of six) from the fits. For A we consider linear, quadratic or cubic polynomials in a^2. In the linear term we allow for a modification by a factor of $\alpha_s^\gamma(a)$, with γ chosen from the set $\{0, 0.5, 1.0, 1.5, 2.0, 2.5\}$. For B and C, we consider both constant and linear polynomials in a^2. For each combination of polynomial orders, we consider the lattice spacing cuts that leave at least one more lattice spacing than the total number of coefficients contained in A or A', as well as at least two more lattice spacings than the maximum number of coefficients contained in B or C.

When presenting results for the observables, we will show continuum extrapolation plots: results as a function of the a^2 for a representative subset of the fits. On the plots the points are obtained by shifting the observables on the ensembles to the physical values of the light and strange masses, using the B and C coefficients from the fit. The fit curves correspond to the fit functions with only the A terms kept.

S5.2 Distribution of observables

For a given observable, fit function, lattice spacing cut and other systematic choices we assign a weight using the Akaike Information Criterion (AIC) [120–122] in a modified version as derived in Ref. [1]:

$$w = \exp\left[-\frac{1}{2}\left(\chi^2 + 2n_{\text{par}} - n_{\text{data}}\right)\right] \ , \tag{S45}$$

computed from the χ^2 of the fit, the number of parameters n_{par}, and the number of data points included in the fit n_{data}. The first two terms in the exponent correspond to the standard AIC, and the last term is introduced to weight fits with a different number of ensembles, due to our cuts in the lattice spacing. From these weighted fits we build a probability distribution from which the central value, statistical and systematic errors for the observable Y are constructed. The technique is described in detail in [1], we briefly summarize it below.

To estimate the statistical and systematic errors, the fit procedure is carried out on each jackknife sample with many different choices of the systematic ingredients. There are two different types of systematics: one where the different possibilities enter with equal weight (flat-weighted) and another where they enter with the AIC weight of their corresponding fit qualities (AIC-weighted). We label collectively the flat-weighted systematics with the indices i, j and the AIC-weighted with the indices a, b. For each analysis, given by a pair of indices (i, a), we have an average value y_{ia}, a jackknife error σ_{ia} and an AIC weight w_{ia} from Equation (S45). From these inputs we construct a probability distribution function (PDF)

for the observable Y

$$\mathrm{PDF}(Y) = \sum_{i,a} \frac{w_{ia} \cdot \mathcal{N}(y_{ia}, \sigma_{ia}; Y)}{\sum_b w_{ib} \cdot \sum_j 1} , \tag{S46}$$

which includes both statistical and systematic variations. The statistical variations are assumed to follow a normal distribution, i.e. $\mathcal{N}(y, \sigma; Y)$ is a normal PDF with mean y and standard deviation σ.

The central value of Y is defined by the median of the constructed PDF. The lower and upper total errors are defined by the standard one-sigma quantiles of the corresponding cumulative distribution function (CDF), close to $16\,\%$ and $84\,\%$. A further estimate of the lower and upper errors comes from halving the intervals obtained from the standard two-sigma quantiles, close to $2\,\%$ and $98\,\%$. Any difference between the two estimates is related to deviations of our distribution away from a normal distribution. To be conservative, for each quantity we consider here, we take the larger of these two error estimates to be our final error. The figures attached to this section show the PDF, the median and corresponding error bands. This procedure gives the total uncertainty of Y. It is also possible to decompose the total error into statistical and systematic components, and the latter into each individual systematic ingredient [1].

In Ref. [123] the same PDF as in Equation (S46) is used, however error estimates are constructed using variances of this distribution instead from quantiles. Also the AIC criterion of [123] differs from ours in Equation (S45) in the way the lattice spacing cuts are implemented. This criterion is derived by assuming that removing a data point is equivalent to adding to the model a parameter that fits the data point exactly. Our AIC criterion is derived by directly computing the dependence of the Kullback–Leibler divergence [124] on the number of data points entering the analysis. For the detailed error budgets given in the following tables we use the variance approach of [123] instead of the procedure used for the total uncertainty, since it gives very similar results for much less computational cost.

S5.3 Combining distributions via random sampling

We also need a technique to perform a stochastic sampling of the previously described PDFs. This becomes useful when combining several such PDFs, each with tens of thousands of analyses, where taking into account all possible combinations would be unfeasible. We perform an importance-weighted stochastic sampling of the systematic ingredients. Let us consider two observables, Y and Z, which share some systematic ingredients with the remaining ones considered independent. Then a random sample is constructed in the following way:

1. We make a common random selection for the systematic ingredients shared by Y and Z. (In our case the shared ingredients are always flat-weighted, so we have a uniform distribution in this step.)

2. We make a random selection for the remaining independent ingredients with a probability given by

$$P(i, a) = \frac{w_{ia}}{\sum_b w_{ib} \sum_j 1} , \tag{S47}$$

   where the shared ingredients i are fixed to the values selected in step 1. This is conveniently accomplished by choosing a uniform random number r in the $[0, 1]$ interval and picking the analysis where the CDF built from the $P(i, a)$ values first reaches the number r. This step has to be done for Y and Z independently.

3. We build the combination observable from Y and Z using the shared and unshared ingredients from steps 1 and 2.

To properly account for statistical correlations, we preserve the jackknife samples during the whole construction. Repeating these steps N_R times, we obtain the desired distribution for the combination observable, which has a single systematic ingredient labelled by the sample index, and each of them comes with a flat weight of $1/N_R$. The statistical, systematic and total errors of the combination can then be obtained by the procedure described above. A similar sampling technique was recently proposed in Ref. [125].

To obtain the physical value of an observable Y we need the physical values of w_0 and M_{ss} as inputs. The corresponding distributions are to be taken from the analyses described in Section S4 and we include them as follows. First we perform the analysis for Y at two fixed values of w_0 and two fixed values of M_{ss}, given by the edges of the central one-sigma bands of their distributions. We label the outcome of these analyses with Y_k with $k = 1, \dots 4$. Then we use the above importance-sampling to select random samples, w_0^r and M_{ss}^r. For a given sample, we perform a bilinear interpolation of the fit values and their corresponding χ^2 values from the Y_k obtained at fixed values of w_0 and M_{ss} to the sampled values w_0^r and M_{ss}^r. Finally, we compute the weights corresponding to the interpolated χ^2 values and perform the above importance-sampling once more to obtain the desired sample Y^r. After repeating N_R times, we obtain the corresponding distribution, which can be handled in the usual way. We find that with $N_R = 10^6$ the stochastic error is well below the uncertainties of our observables, so we use this value in our combinations.

S6 Window observables

The lattice contribution of this work consists of computing the leading-order hadronic contribution to the muon magnetic moment, $a_\mu^{\mathrm{LO-HVP}}$, from zero Euclidean distance to $t_{\mathrm{cut}} = 2.8$ fm. Both in our 2017 [24] and in our 2020 work [1] we used lattice results up to a given temporal distance, beyond which upper and lower bounds were used to constrain the long-distance tail of the light and disconnected correlators. We chose $t_{\mathrm{cut}} = 3$ fm and 4 fm in 2017 and 2020, respectively. In the present paper we improve on this procedure and beyond $t_{\mathrm{cut}} = 2.8$ fm we use state-of-the-art results from a data-driven approach. The reasons for choosing this value of t_{cut} and details of the approach are described in Section S10.

Regarding $a_\mu^{\mathrm{LO-HVP}}$ and its various contributions we use the definition and notations from our 2020 work. In particular, since we consider only the LO-HVP contribution, we drop the superscript and multiply the result by 10^{10}, i.e. a_μ stands for $a_\mu^{\mathrm{LO-HVP}} \times 10^{10}$ throughout the Supplementary Information. We will consider several different window observables, where we restrict the integration in Euclidean time to a region between t_1 and t_2 with the standard window function defined in Ref. [14]. We use the notation $15-19$ for a window between $t_1 = 1.5$ fm and $t_2 = 1.9$ fm and accordingly for the other windows. An important feature of the definition is that two adjacent windows can be joined by simple addition, like $04-10$ and $10-28$ equals $04-28$.

A minor difference compared to our 2020 work concerns the splitting up of a_μ into nonperturbative and perturbative parts. Back then we introduced a momentum $Q_{\mathrm{max}} = \sqrt{3}$ GeV to separate the two regions: below Q_{max} we used the lattice computation, above that perturbation theory. Here we remove this separation by sending Q_{max} to infinity. In practice this change only affects the $00-04$ window.

In this work we compute all major contributions to a_μ: light, strange, charm and disconnected[10]. In our earlier work for each individual flavour component we considered the contributions to a_μ obtained by integrating the correlator over all Euclidean times. Here we cut the integral via a one-sided window function that ends at $t = t_{\mathrm{tail}}$, eliminating a noisy contribution with large finite-size and taste-violation effects. We also split the time into several windows. This allows the fit functions to be different in the different windows, giving us more control over statistical and systematic errors. Regarding finite-size effects we perform the continuum extrapolations in a finite box, called the reference box, with L_{ref} spatial and T_{ref} temporal extent. To get the infinite-volume result we compute corrections in dedicated lattice simulations, as described in Section S7.

In the following subsections we begin by presenting our blinding procedure. Then we show results for several windows that are available in the literature. In particular we consider the short-distance window $00-04$, the intermediate-distance window $04-10$, and also a window at longer distances $15-19$. For some of these we use a subset of the configurations. On these observables we show our fit procedure in detail. The results can then be also compared with those in the literature[11]. Afterwards we explain how

[10]By disconnected contributions we refer to the disconnected diagrams of light and strange quarks.

[11]We perform the comparisons in the isospin-symmetric theory, where a scheme ambiguity is present, see Section S4.

we obtain the lattice result for the window $00-28$. Then we describe how the rest of the contributions (strange, charm and isospin breaking) were obtained. Finally, we combine them with the tail contribution to get the all-flavour result for a_μ. In this final section we also give all-flavour results in the intermediate $04-10$ and long-distance $10-\infty$ windows.

The results for the different windows will be presented in Tables and here we give their general description. In the first row, continuum extrapolations as a function of a^2 are shown. For readability, the points are projected to the physical quark masses using an appropriate fit from the plot. The normalized weight of each fit is indicated via the plotted colour scale. For even better readability we also make the data points and fits appearing in the plots available under Reference [126]. In the second row we show with red the probability distribution function including both statistical and systematic variations. The median is given by the blue vertical line, the 1-sigma and 2-sigma bands, shown in green/yellow, contain 68.3% and 95.4% of the distribution, centred at the median. Where results from other collaborations are available in the literature, we show them in another figure in the second row. The references to the other works are then given in the text. If available, the statistical error is given as an inner error-bar. The table in the third row contains the number of fits, the percentage of fits having a P-value larger than 0.1, the median of the observable, the total, statistical and systematic uncertainties and an error budget. All results correspond to the reference box-size, $L_{\rm ref}=6.272$ fm, except in the comparison plot, where infinite-volume results are given.

S6.1 Blinding

To eliminate human bias we performed our analysis in a blinded fashion with independent crosschecks at each step. When constructing the window observables from the current correlation functions they are multiplied by a window dependent blinding factor. The full analysis from fitting the window observables to performing the error analysis on the global fits is implemented and performed independently by two or more groups, who do not know the values of the blinding factors. The results of the analysis are then crosschecked at each stage. In every case consensus was reached. Once the analysis procedures were finalized with full crosschecks, the blinding factors were revealed and divided out to obtain the final results. A special script was written, which carried out the unblinding, added the results to the manuscript and produced the plots of the paper, without the need for human intervention. Our procedure guarantees that no bias was involved in obtaining the final results.

After unblinding, and during the peer review process, concerns were raised 1. about consistency of window functions for the isospin-breaking corrections; 2. about the possibility of an unknown excited-state systematic in the lattice determination of the Ω mass, as described in Section S2; and 3. about the validity of continuum extrapolations with polynomials of $\Delta_{KS}(a)$. Based on these criticisms, 1. the isospin-breaking corrections were re-computed with the exact window functions; 2. the value of w_0 scale was instead calculated in an alternate blinded analysis based upon the experimental muonic pion decay rate, see Section S3; 3. the window observables were re-calculated in an alternate blinded analysis replacing $\Delta_{KS}(a)$ by $a^2\alpha_s(a)^\gamma$, as described in Section S5. The values of the isospin-breaking corrections, the w_0 and the window observables from these new determinations were computed blind and not known at the time of making our decisions about the final procedure, but the original isospin-breaking corrections, the w_0 and window values from the old analyses were.

In response to referee concerns, additional uncertainties were added to the long-distance tail contributions discussed in Section S10 after unblinding. These only increased the uncertainty of this small contribution without changing the central value.

The net effect of these four changes on the final result was to increase the central value by 1.0 (0.14% of the final value), and increase the uncertainty by 0.1 (3% of the total uncertainty). This differences are very small, and much smaller than the uncertainty of our result.

This has been studied numerically in the recent work of RBC [38]. In case of the $04-10$ window an ambiguity of $\delta a_{\mu,04-10}^{\rm light}=0.10(24)(07)$ was found, much smaller than current uncertainties.

S6.2 Short-distance window

The short distance window is plagued by lattice artefacts that are logarithmically enhanced compared to the usual case with on-shell observables [127]. These artefacts arise from arbitrary small time separations: $a_{\mu,00-04}$ is not an on-shell quantity. We can improve the behaviour by removing part of these discretization errors using lattice perturbation theory. We define our tree-level improved observable by the transformation

$$a_{\mu,00-04}^{\text{light}} \to a_{\mu,00-04}^{\text{light}} + a_{\mu,00-04}^{\text{tree}}(0) - a_{\mu,00-04}^{\text{tree}}(a) \ , \tag{S48}$$

where tree stands for leading-order, infinite-volume, massless staggered perturbation theory. The positive effect of the improvement can be seen in the top figure of Table S6 for the light case. We only apply this improvement to the light contribution, since the disconnected component vanishes at this order in perturbation theory.

We also investigate the lattice artefacts by varying the kernel function the current correlator is multiplied with. In particular we change the square bracket in Equation (65) of [1]

$$\left[t^2 - \frac{4}{(aQ)^2}\sin^2\left(\frac{aQt}{2}\right)\right] \to \left[t^2 - \frac{4}{(a\hat{Q})^2}\sin^2\left(\frac{aQt}{2}\right)\right] \tag{S49}$$

where Q is the momentum of the hadronic vacuum polarization and $\hat{Q}$ denotes the lattice momentum defined by $\hat{Q} = 2\sin(aQ/2)/a$. The obtained data points are also shown in the top figure of Table S6 for the light and that of Table S7 for the disconnected contribution. Lattice artefacts are larger with $\hat{Q}$. In tree-level perturbation theory one can show that the coefficient of the logarithmically enhanced cutoff effect has a different sign, if one uses Q or $\hat{Q}$ in the kernel. The light results with $\hat{Q}$ approach the continuum limit from below, whereas the ones with Q show a characteristic turnover behaviour.

In our analyses we include data with and without tree-level improvement and also using Q and $\hat{Q}$. Tree-level improvement may not remove all logarithmic cutoff effects, so we also include fit functions with logarithmic terms. The a-dependent part of our fit functions is as follows:

$$A_2a^2 + A_4a^4 \ , \quad A_2a^2 + A_la^2\log(a^2/w_0^2) \ , \quad A_2a^2 + A_4a^4 + A_la^2\log(a^2/w_0^2) \ . \tag{S50}$$

Note that the true asymptotic form of the discretization errors is unknown, since there is no theory analogous to Symanzik's for the discretization errors of this short-distance quantity. Therefore, the choices given in (S50) are phenomenological.

The results for in the light case can be found in Table S6. To this we can add the finite-size correction from Table S21 to get

$$a_{\mu,00-04}^{\text{light}} = 47.85(5)(13)[14] \tag{S51}$$

with statistical, systematic and total errors. This is our infinite-volume result for the light contribution to the short-distance window, which is compared in the Table to previous lattice results of ETM [36], RBC/UKQCD [38], Mainz/CLS [44], Fermilab Lattice/HPQCD/MILC [45] and Spiegel & Lehner [46]. The results for the disconnected case can be found in Table S7. Note that the number of fits is smaller than in the light case because we have measurements on fewer lattice spacings. Again we perform the finite-size correction and obtain

$$a_{\mu,00-04}^{\text{disc}} = -0.0014(22)(101)[104] \tag{S52}$$

as the infinite-volume result. The relatively large systematic error comes from the uncertainty of the $I = 0$ finite-size effect. This value is compared to the results of ETM [36], Mainz/CLS [44] and Fermilab Lattice/HPQCD/MILC [45] in a figure of the Table.

In order to verify the validity of our continuum extrapolation procedure in the short distance region, we compare our lattice results with the predictions of perturbation theory. For this purpose we calculated light windows $00-01$, $00-02$ and $00-03$ using the same procedure as was used for the short distance

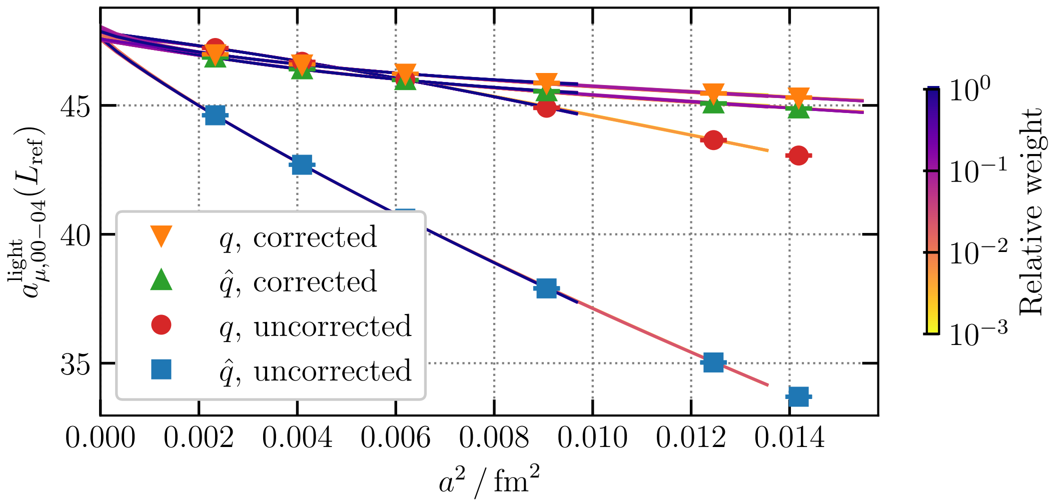

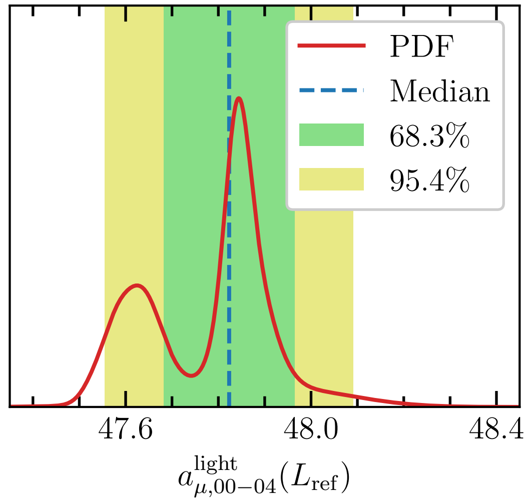

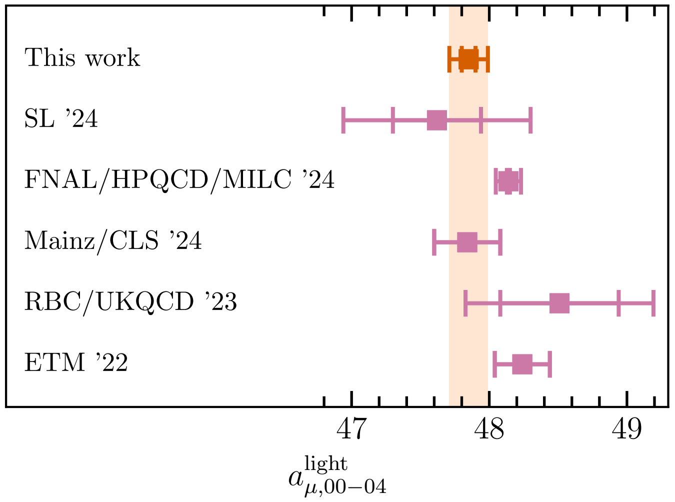

Number of fits	8192	
Fits with $P > 0.1$	54%	
Median	47.82	
Total error	0.14	0.29%
Statistical error	0.05	0.10%
Systematic error	0.13	0.28%
Pseudoscalar fits	0.00	0.00%
M_{ss} value	0.00	0.00%
w_0 value	0.01	0.02%
Tree correction and/or $\hat{q}$	0.09	0.19%
Lattice spacing cut	0.05	0.11%
Fit polynomial order	0.06	0.12%

Table S6: Light-connected window observable $a^{\text{light}}_{\mu,00-04}$. The plot conventions are described in the first part of Section S6. The continuum extrapolations are shown with two different kernel functions, denoted by q and $\hat{q}$. The probability distribution function displays two dominant peaks, corresponding to the variation between q and $\hat{q}$ in the uncorrected case. We compare our result with others from the literature [36, 38, 44–46].

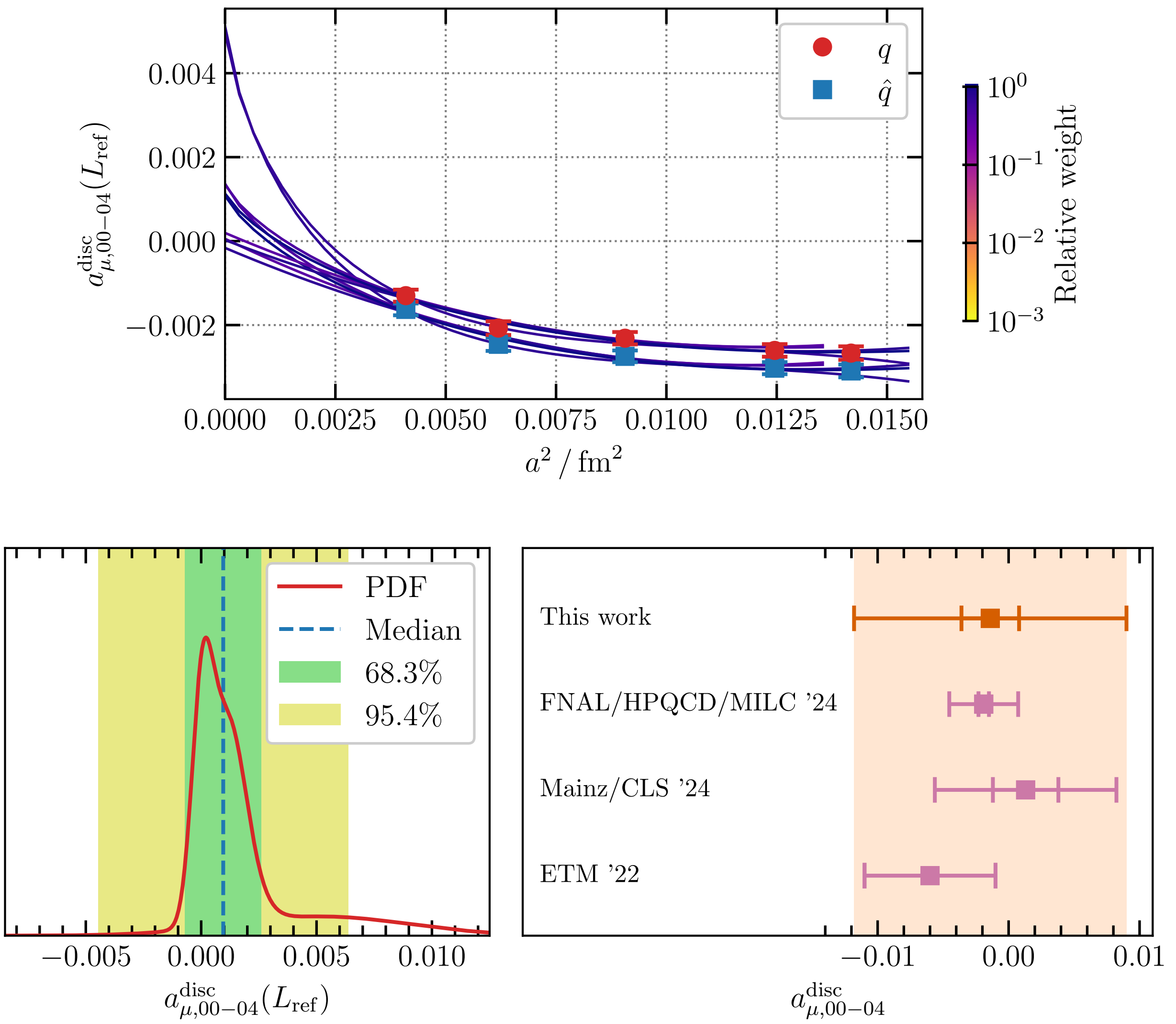

Number of fits	2560	
Fits with $P > 0.1$	100%	
Median	0.0010	
Total error	0.0027	284%
Statistical error	0.0022	226%
Systematic error	0.0016	172%
Pseudoscalar fits	0.0000	1%
M_{ss} value	0.0000	1%
w_0 value	0.0002	16%
Tree correction and/or $\hat{q}$	0.0001	6%
Lattice spacing cut	0.0005	47%
Fit polynomial order	0.0018	192%

Table S7: Disconnected window observable $a^{\rm disc}_{\mu,00-04}$. The plot conventions are described in the first part of Section S6. The continuum extrapolations are shown with two different kernel functions, denoted by q and $\hat{q}$. We compare our result with others from the literature [36, 44, 45].

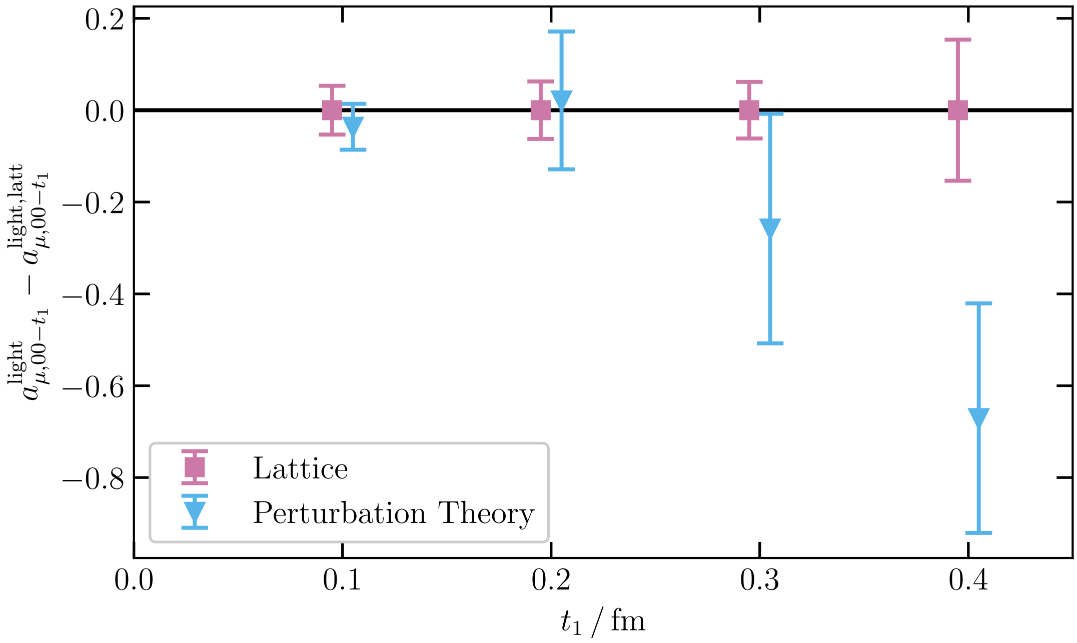

Figure S12: Comparison between the lattice results and those obtained from perturbation theory for the short distance window observables $a^{\text{light}}_{\mu,00-t_1}$ as a function of t_1. The points have the central value of the lattice results subtracted off, and a slight horizontal offset for readability.

window $00-04$. Additionally, we calculated the same quantities, using $N_f = 4$ massless perturbation theory [128]. We change the scale parameter μ in the range $[0.8, 2]\,\text{GeV}$ and this variation enters as the systematic uncertainty of the perturbative calculation. In Figure S12 we plot the lattice and perturbative results for the light windows $a^{\text{light}}_{\mu,00-t_1}$ as the function of t_1. It can be clearly seen that for shorter distances $00-01$, $00-02$ and $00-03$ perturbation theory agrees with the lattice data.

S6.3 Intermediate-distance window

Let us consider first our results for the light-connected part of the so-called intermediate-distance window, the window $04-10$ in our notation. This is a quantity designed to compare different lattice calculations in a controlled way, since its systematic errors are usually well under control. The results are shown in Table S8. The dominant uncertainties are the scale setting error and the one arising from the variation of the polynomial order of the fit functions. After applying the finite-size correction we get the infinite-volume result as

$$a^{\text{light}}_{\mu,04-10} = 206.92(37)(34)[50]\ . \tag{S53}$$

The comparison with the literature is shown in the figure of the second row of Table S8 where, besides our current and previous results, those of Lehner and Meyer [32], χQCD [33], Aubin et al [34], Mainz/CLS [35], ETM [36], RBC/UKQCD [38] and Fermilab Lattice/HPQCD/MILC [45] are given. Also shown are two results from the data-driven approach: the determination from our 2020 work, where we subtracted the other-than-light lattice contributions from the total data-driven value, and a recent pure data-driven computation from Benton et al [39].

In order to quantify the improvement on the uncertainty of $a^{\text{light}}_{\mu,04-10}$ due to the new $a = 0.048\,\text{fm}$ ensemble, we compare the results obtained with and without that ensemble. For consistency, the latter is computed using the distribution of w_0 obtained also without the new ensemble. We obtain $206.48(50)(74)[90]$ without finite volume corrections, to be compared with the median number and errors in Table S8. Accordingly, the error reduction due to the finest ensemble is $1 - 0.50/0.90 = 44\%$.

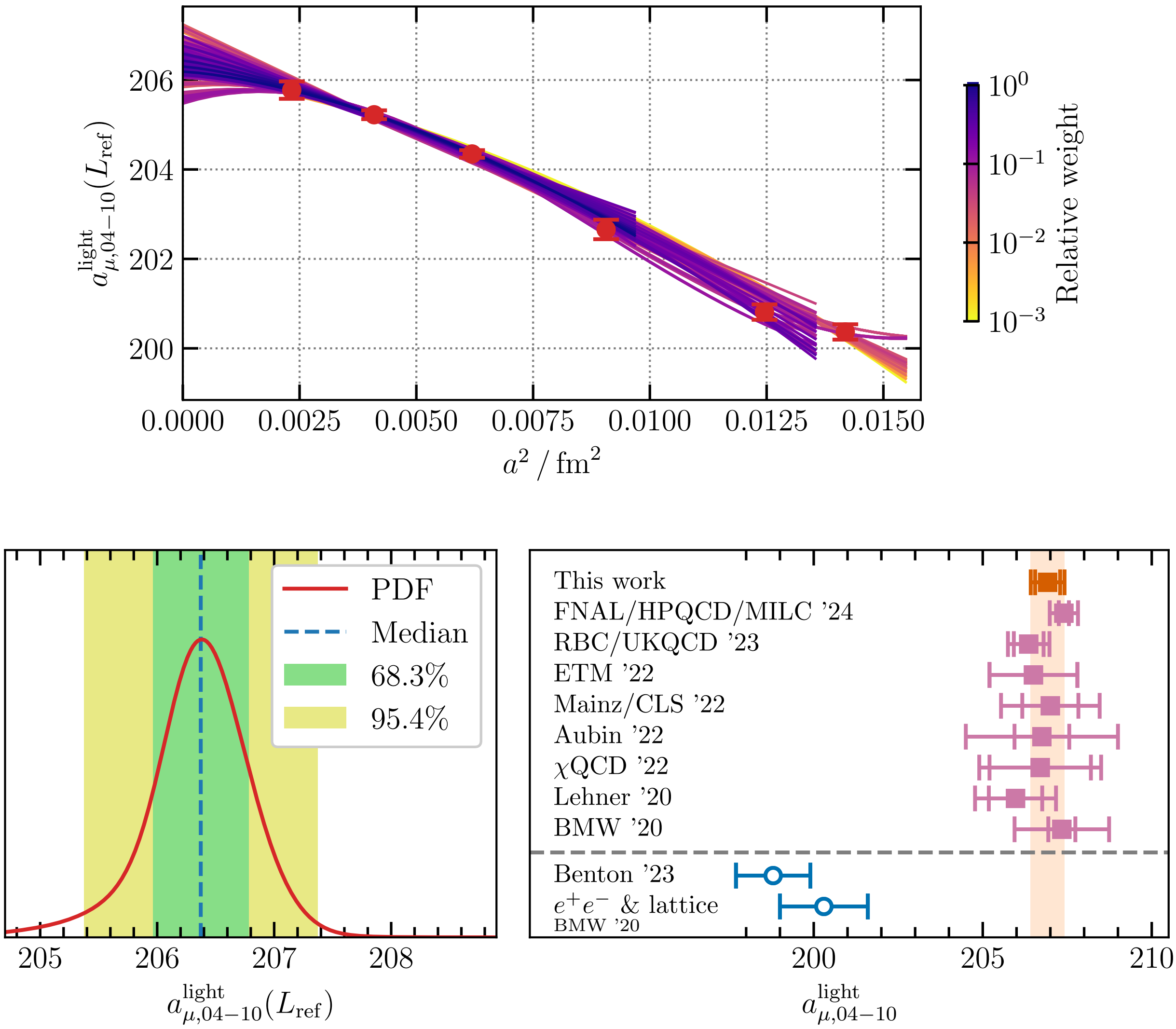

Number of fits	50688	
Fits with $P > 0.1$	80%	
Median	206.37	
Total error	0.50	0.24%
Statistical error	0.37	0.18%
Systematic error	0.33	0.16%
Pseudoscalar fits	0.01	0.00%
M_{ss} value	0.01	0.00%
w_0 value	0.18	0.09%
Lattice spacing cut	0.07	0.04%
Fit polynomial order	0.18	0.09%
Log corrections γ	0.10	0.05%

Table S8: Light-connected window observable $a^{\text{light}}_{\mu,04-10}$. The plot conventions are described in the first part of Section S6. We compare our result with others from the literature, both lattice [1, 32–36, 38, 45] and data-driven [1, 39].

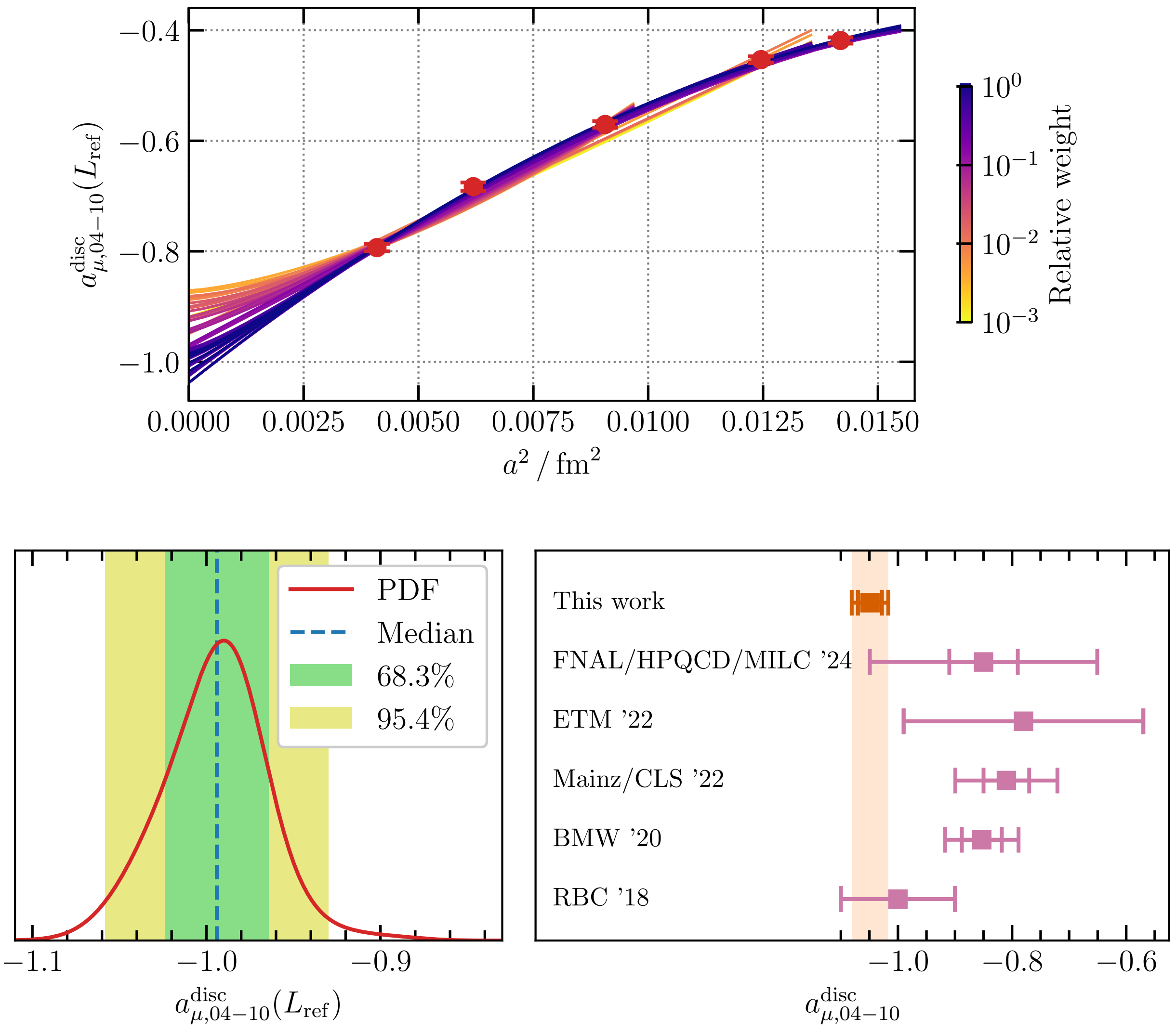

Number of fits	30720	
Fits with $P > 0.1$	48%	
Median	-0.994	
Total error	0.032	-3.2%
Statistical error	0.021	-2.1%
Systematic error	0.024	-2.4%
Pseudoscalar fits	0.000	-0.0%
M_{ss} value	0.000	-0.0%
w_0 value	0.008	-0.8%
Lattice spacing cut	0.013	-1.3%
Fit polynomial order	0.012	-1.2%
Log corrections γ	0.015	-1.5%

Table S9: Disconnected window observable $a^{\rm disc}_{\mu,04-10}$. The plot conventions are described in the first part of Section S6. We compare our result with others from the literature [1, 14, 35, 36, 45].

In the disconnected case the results are given in Table S9. The analysis gives

$$a_{\mu,04-10}^{\mathrm{disc}} = -1.049(21)(24)[32] \ , \tag{S54}$$

again after the application of the finite-size correction. We also provide a comparison to the results of RBC/UKQCD [14], Mainz/CLS [35], ETM [36] and Fermilab Lattice/HPQCD/MILC [45]. The difference compared to the value of our 2020 work comes from the addition of a new lattice spacing and that now we also include quadratic fits in the analysis - previously we only used linear continuum extrapolations.

S6.4 Long-distance window $15-19$

A longer-distance window was proposed by Aubin et al [34], for the range $1.5-1.9$ fm, which is called "W2" there and corresponds to $15-19$ in our notation. A feature of this window is the strong presence of taste-breaking effects. Here we apply the same taste improvement procedure that we had in our 2020 work. For obtaining the central value of our result, we use the staggered version of the rho-pion-gamma model (SRHO) of Jegerlehner and Szafron [129], originally proposed by Sakurai [130]. In the context of removing taste-breaking effects in a_μ, it has already been used by the HPQCD collaboration [131]. In order to assign a systematic error associated with the taste improvement, we also compute the taste-breaking effects using next-to-next-to-leading order staggered chiral perturbation theory (NNLO SXPT) [25, 132–134].

The data with these improvements and also without any improvement are shown in the top figures of Table S10. There is a strong non-linearity in the unimproved data, that gets eliminated by the improvement. In our analysis we use the SRHO model to get the central value of our result. As a systematic error associated with the taste improvement we assign the difference of the result obtained with NNLO SXPT and SRHO. We also assign a systematic to the choice of the starting point of the taste improvement by performing the analysis with two different values of this point, 1.2 fm and 1.4 fm. Due to taste improvement the number of fits is higher than it is in the $04-10$ window. We obtain

$$a_{\mu,15-19}^{\mathrm{light}} = 97.57(1.76)(1.17)[2.11] \tag{S55}$$

in the infinite-volume limit. This value, together with those of Fermilab Lattice/HPQCD/MILC [37] and of Aubin et al [34], is shown in the second row of Table S10.

S6.5 Long-distance window $10-28$

We also compute the light-connected $10-28$ window, which, together with contributions from other flavours and isospin-breaking, will be complemented by a data-driven determination of the $28-\infty$ window to arrive at a hybrid result of the all-flavour, $10-\infty$ window. The analysis of the $10-28$ window proceeds the same way as above, and the results are presented in Table S11. Regarding the disconnected contribution, we fit a single window $10-28$ and use the following combination:

$$a_{\mu,10-28}^{I\approx 0} \equiv \tfrac{1}{10} a_{\mu,10-28}^{\mathrm{light}} + a_{\mu,10-28}^{\mathrm{disc}} . \tag{S56}$$

This definition combines the light and disconnected contributions as they appear in the $I=0$ channel. The total $I=0$ also contains the contributions of the more massive $s, c, \ldots$ quarks, which are not included in $a_{\mu,10-28}^{I\approx 0}$. The advantage of this observable is that the large taste-breaking and finite-size effects of the $I=1$ channel are cancelled. The results are shown in Table S12. The continuum extrapolation is almost completely flat; the largest source of error here is statistical.

Using the sampling procedure of Section S5.3 we combine the light-connected and the $I\approx 0$ observable as

$$a_{\mu,10-28}^{\mathrm{light+disc}} = \tfrac{9}{10} a_{\mu,10-28}^{\mathrm{light}} + a_{\mu,10-28}^{I\approx 0} \tag{S57}$$

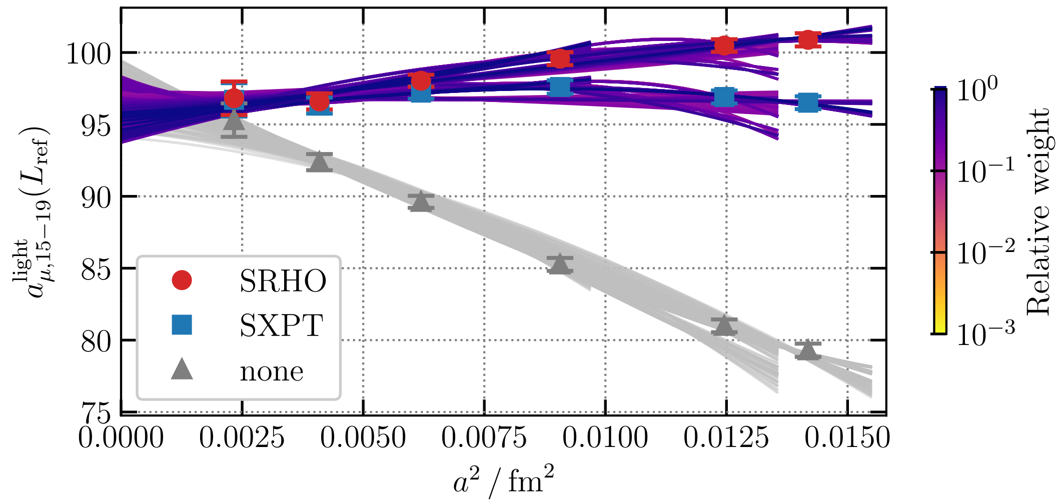

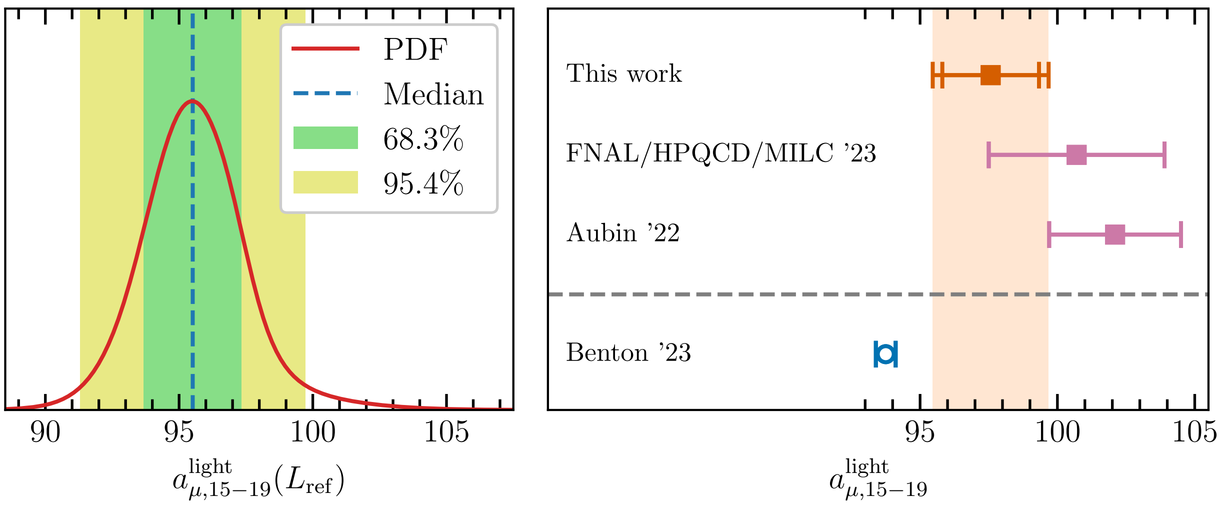

Number of fits	50688	
Fits with $P > 0.1$	100%	
Median	95.51	
Total error	2.10	2.20%
Statistical error	1.75	1.84%
Systematic error	1.16	1.22%
Pseudoscalar fits	0.02	0.02%
M_{ss} value	0.02	0.02%
w_0 value	0.62	0.65%
Taste correction	0.28	0.30%
Lattice spacing cut	0.22	0.23%
Fit polynomial order	0.62	0.64%
Log corrections γ	0.20	0.21%

Table S10: Light-connected window observable $a^{\text{light}}_{\mu,15-19}$. The plot conventions are described in the first part of Section S6. The continuum extrapolations are shown with no, NNLO XPT and SRHO taste improvements. We compare our results with others from the literature, both lattice [34, 37] and data-driven [39].

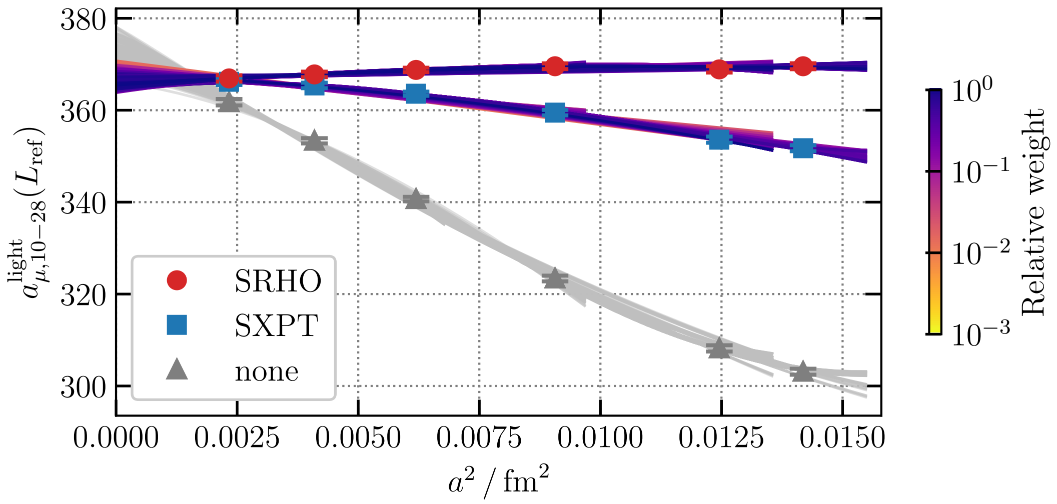

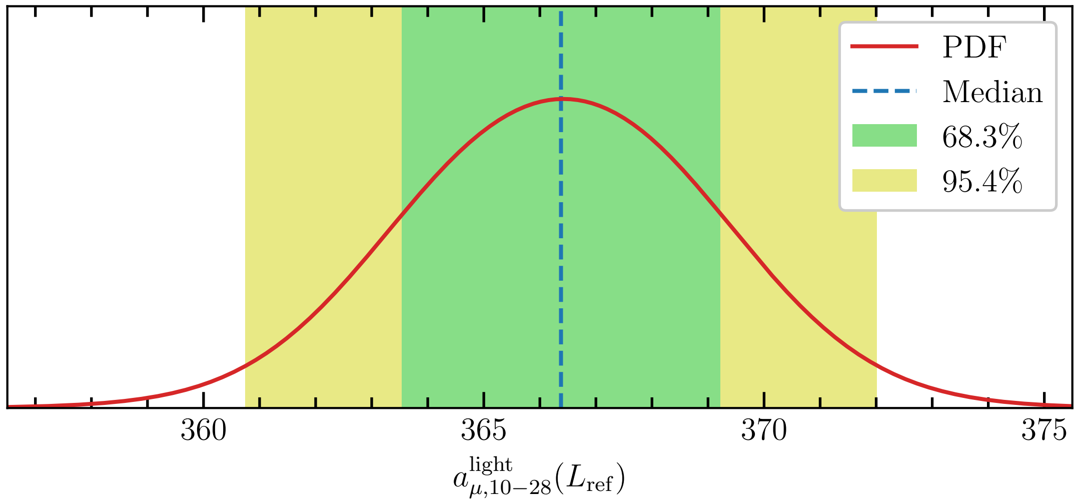

Number of fits	50688	
Fits with $P > 0.1$	99%	
Median	366.38	
Total error	2.84	0.78%
Statistical error	1.76	0.48%
Systematic error	2.23	0.61%
Pseudoscalar fits	0.04	0.01%
M_{ss} value	0.03	0.01%
w_0 value	1.64	0.45%
Taste correction	0.90	0.25%
Lattice spacing cut	0.40	0.11%
Fit polynomial order	0.33	0.09%
Log corrections γ	0.36	0.10%

Table S11: Light-connected window observable $a^{\text{light}}_{\mu,10-28}$. The plot conventions are described in the first part of Section S6. The continuum extrapolations are shown with no, NNLO XPT and SRHO taste improvements.

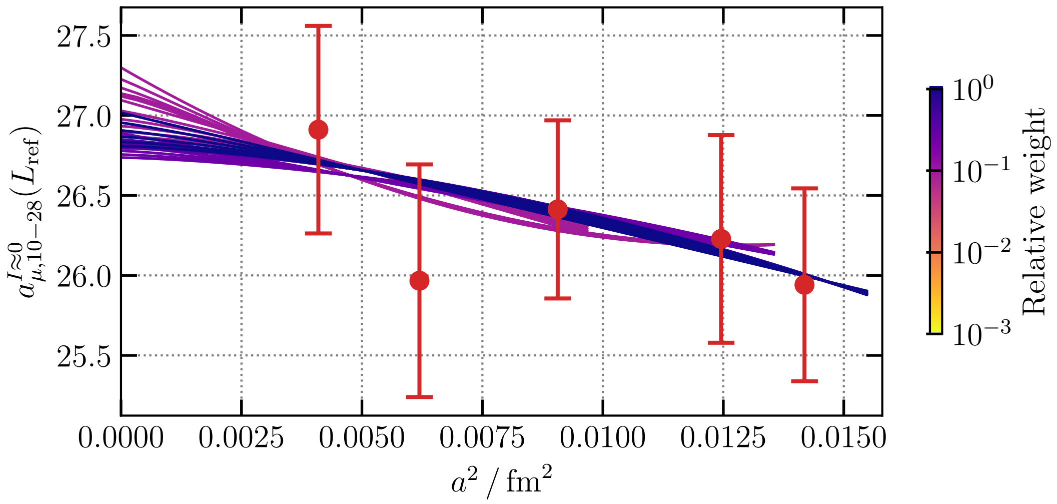

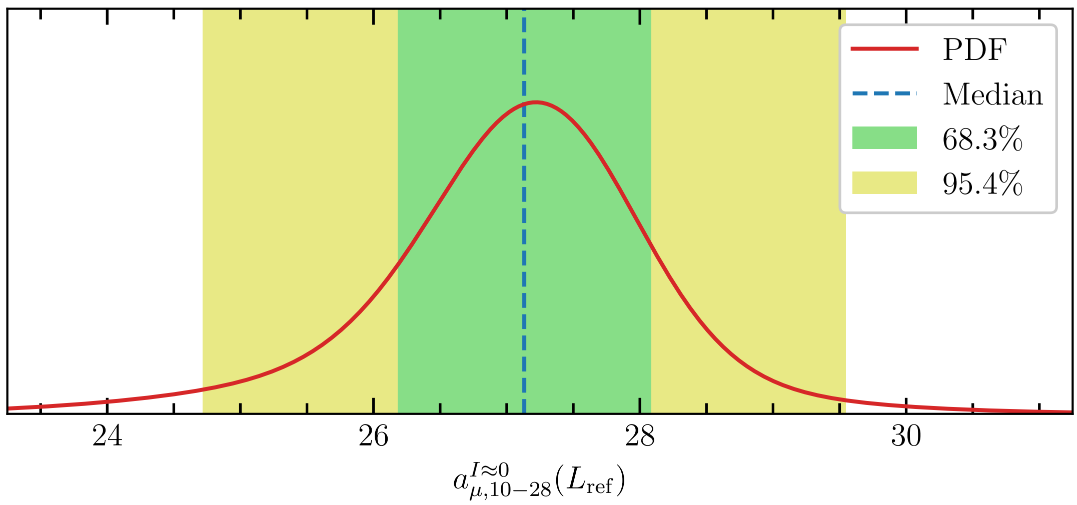

Number of fits	7680	
Fits with $P > 0.1$	100%	
Median	27.13	
Total error	1.21	4.45%
Statistical error	1.18	4.36%
Systematic error	0.24	0.88%
Pseudoscalar fits	0.01	0.03%
M_{ss} value	0.01	0.02%
w_0 value	0.14	0.53%
Lattice spacing cut	0.03	0.12%
Fit polynomial order	0.22	0.83%
Log corrections γ	0.00	0.01%

Table S12: $I \approx 0$ window observable $a^{I\approx 0}_{\mu,10-28}$. The plot conventions are described in the first part of Section S6.

also taking into account the distributions of our scale-setting quantities w_0 and M_{ss}. We obtain

$$a_{\mu,10-28}^{\text{light+disc}} = 365.6(2.4)(2.0)[3.1] \tag{S58}$$

in infinite volume, where we also added the finite-size corrections, both to the $I = 1$ and $I = 0$ values, from Table S21.

S6.6 All-distance window $00 - 28$

Our procedure for the $00-28$ window introduces breakpoints at 0.4 fm, 2.0 fm and 2.4 fm time separations, the corresponding window observables are denoted:

$$a_{\mu,00-04} \;, \quad a_{\mu,04-20} \;, \quad a_{\mu,20-24} \;, \quad a_{\mu,24-28} \;. \tag{S59}$$

We perform the analyses for these observables and sum them up to get the $00-28$ contribution. The first one, $00 - 04$, is the short distance window, which we have already presented. The other three windows are fitted together. In the end, we combine the short distance window with the three others and also with the distributions of w_0 and M_{ss}, as described in Section S5.3.

In choosing the times at which the different windows begin and end, one must make sure that they do not become too narrow, to avoid lattice artefacts, nor too numerous, to avoid problems associated with strong correlations. We allow the fit functions to be different in each window; the χ^2 of the fit includes the residuals and also their correlation matrix. We then loop over all possible combinations of the fit functions and apply our standard analysis procedure, as described in Section S5. There is a clear advantage of this procedure over fitting the total $04 - 28$ range at once. Namely, we can have high-order fit functions at shorter distances, where the data is precise, while we can use low-order fit functions at longer distances, where the data is noisy. The favourable combinations are selected automatically by the AIC weight.

We perform the multi-window analysis for different partitions of the $04 - 28$ interval, varying the number of windows and the times at which they are joined (see Figure S13). We then monitor the continuum extrapolation errors. For this study, the uncertainty coming from scale-setting is irrelevant and therefore not included. However, we note that it still is the dominant source of systematic uncertainty in the $04 - 28$ window. When using two windows instead of one, we find the optimal split to be at 2.4 fm, which decreases the error by about 13%. We also look at splitting the interval into three windows and find a small improvement with respect to the two-window case. As a result, we choose the three windows, $04-20$, $20-24$ and $24-28$, for our final analysis. The window-independent systematics like scale setting, pseudoscalar fit ranges or taste-improvement procedure give us 256 variations that are shared among the three windows. Then in each window we have 198 different fit functions, lattice spacing cuts, and so on. We use all possible combinations of these, so we perform $256 \times 198 \times 198 \times 198$ fits altogether. Representative continuum extrapolations, histograms and error budgets are shown in Table S13.

Regarding the disconnected contribution, we fit a single window $04-28$ and use the $I \approx 0$ combination as in Equation (S56). The results are shown in Table S14.

Finally, we combine the three-window fits of the light, the single-window fit of the $I \approx 0$ observable in the $04 - 28$ window and the light and disconnected contributions in the $00 - 04$ window

$$a_{\mu,00-28}^{\text{light+disc}} = \frac{9}{10} a_{\mu,04-28}^{\text{light}} + a_{\mu,04-28}^{I\approx 0} + a_{\mu,00-04}^{\text{light}} + a_{\mu,00-04}^{\text{disc}} \tag{S60}$$

also taking into account the distributions of our scale-setting quantities w_0 and M_{ss}. We obtain

$$a_{\mu,00-28}^{\text{light+disc}} = 619.6(2.3)(2.1)[3.1] \tag{S61}$$

in infinite volume, where we added the finite-size correction from Table S21.

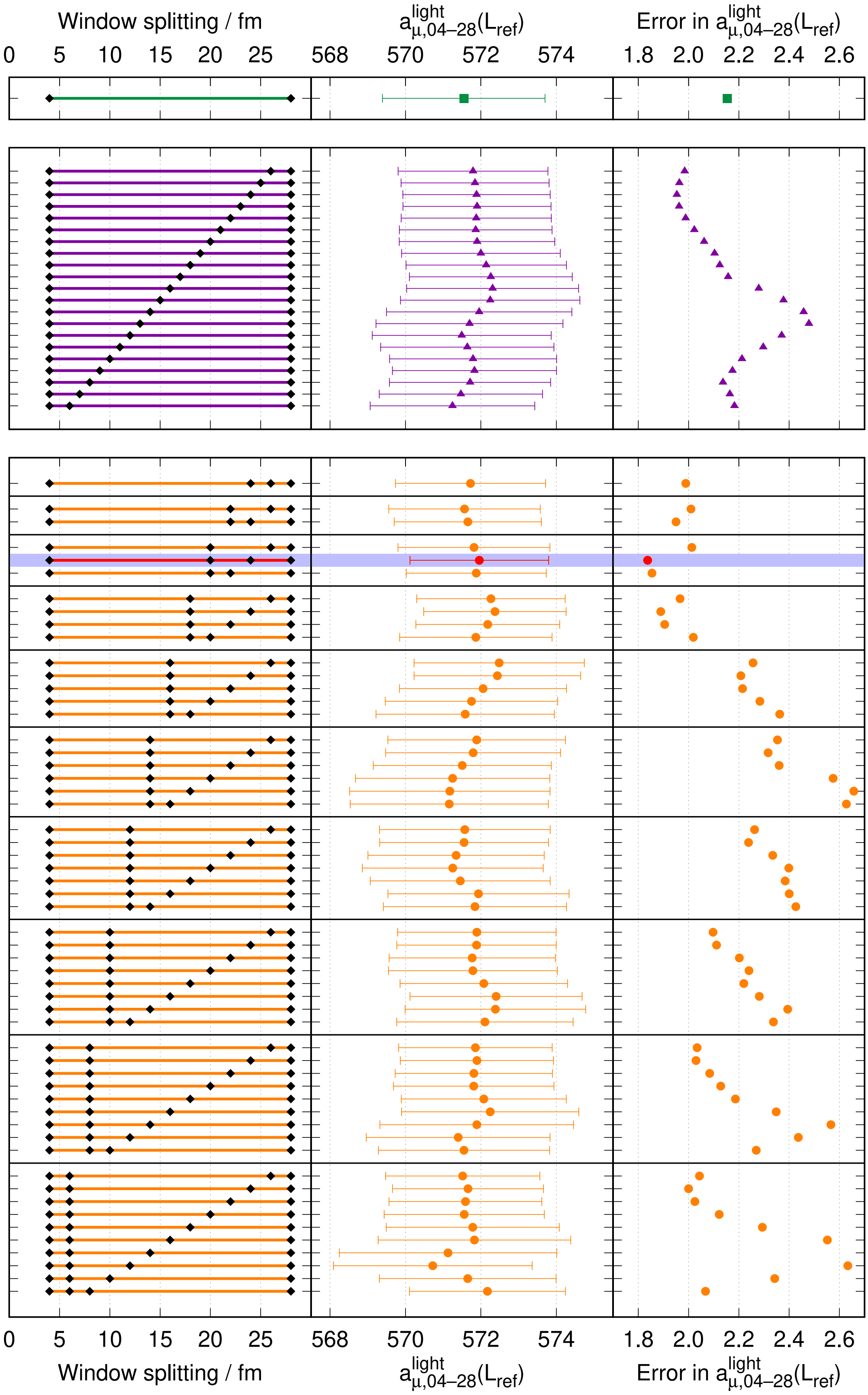

Figure S13: Value and error of the light-connected window observable $a_{\mu,04-28}^{\text{light}}$ depending on the fitting procedure. Each line corresponds to a particular splitting of the window $04-28$. The green point (first panel) corresponds to fitting the window $04-28$ without splitting. The purple points (second panel) show the values and errors when splitting into two windows was used. The orange points (third panel) show the case of splitting the window $04-28$ into three parts. The first column demonstrates the type of splitting. The second column shows the value and error of the result of the particular fitting procedure. The third column shows the error once again for better readability. Our choice, the splitting into windows $04-20$, $20-24$ and $24-28$, is highlighted with a blue shaded band and coloured red. Note that the shown errors do not include the uncertainties arising from the scale setting, which is actually the dominant source of systematics in this window.

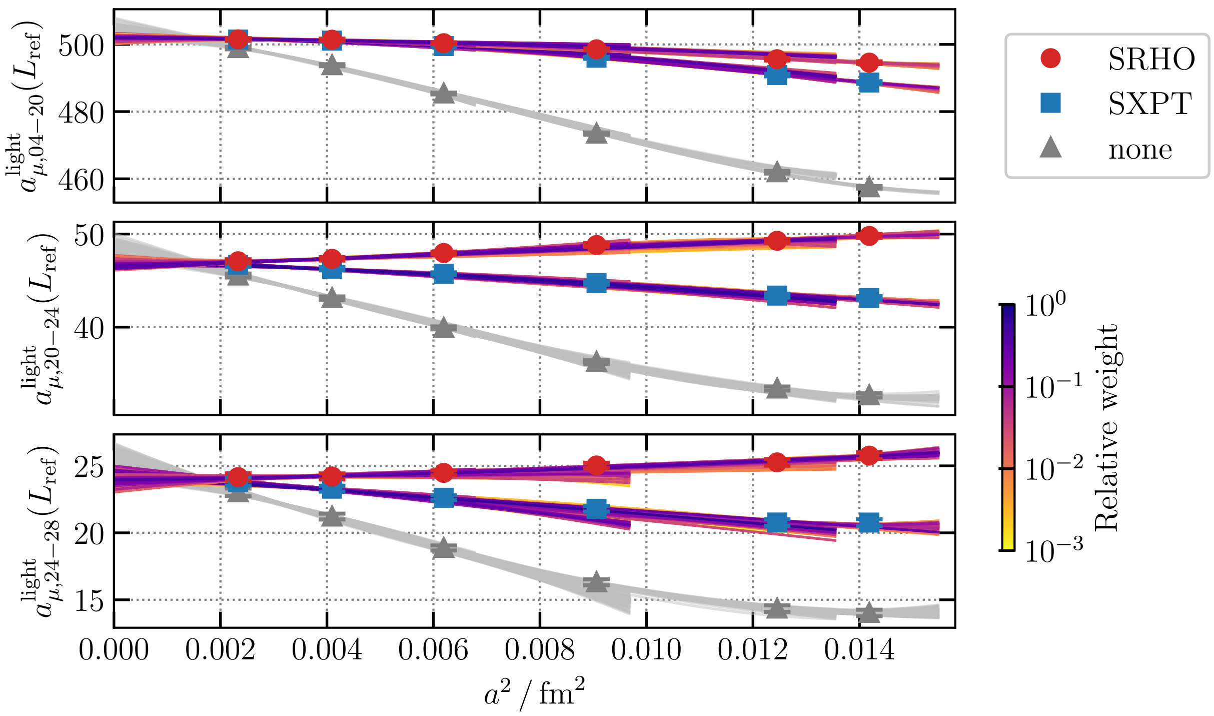

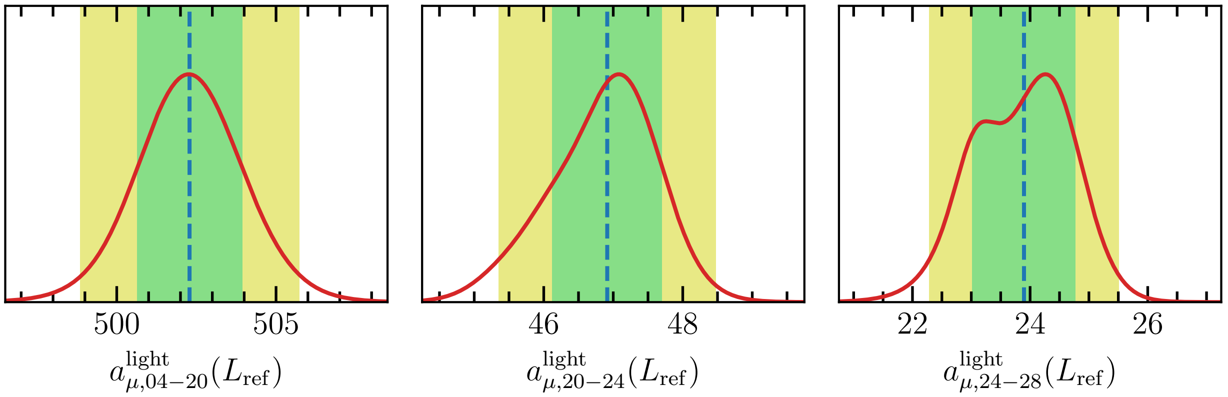

Number of fits	248396544							
Fits with $P > 0.1$	80%							
Median	572.99		502.28		46.91		23.90	
Total error	2.75	0.48%	1.72	0.34%	0.79	1.68%	0.88	3.67%
Statistical error	1.84	0.32%	1.24	0.25%	0.41	0.88%	0.41	1.70%
Systematic error	2.04	0.36%	1.19	0.24%	0.67	1.43%	0.78	3.25%
Pseudoscalar fits	0.05	0.01%	0.03	0.01%	0.02	0.03%	0.01	0.04%
M_{ss} value	0.03	0.01%	0.02	0.00%	0.01	0.02%	0.00	0.02%
w_0 value	1.72	0.30%	1.20	0.24%	0.31	0.66%	0.21	0.86%
Taste correction	0.86	0.15%	0.31	0.06%	0.32	0.69%	0.45	1.89%
Lattice spacing cut	0.40	0.07%	0.16	0.03%	0.20	0.43%	0.15	0.62%
Fit polynomial order	0.40	0.07%	0.23	0.05%	0.20	0.42%	0.27	1.11%
Log corrections γ	0.23	0.04%	0.06	0.01%	0.08	0.17%	0.10	0.42%
	$04-28$		$04-20$		$20-24$		$24-28$	

Table S13: Light-connected window observable $a^{\rm light}_{\mu,04-28}$ obtained as sum of three windows $04-20$, $20-24$ and $24-28$, fitted together. The plot conventions are described in the first part of Section S6. The continuum extrapolations are shown with no, NNLO XPT and SRHO taste improvements. The two-peak structure in the $24-28$ histogram comes from the taste improvement variation.

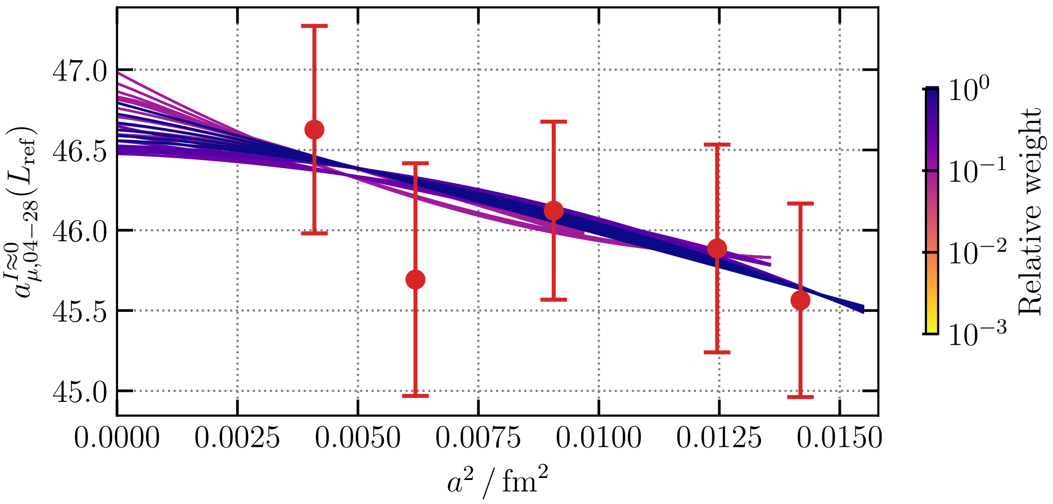

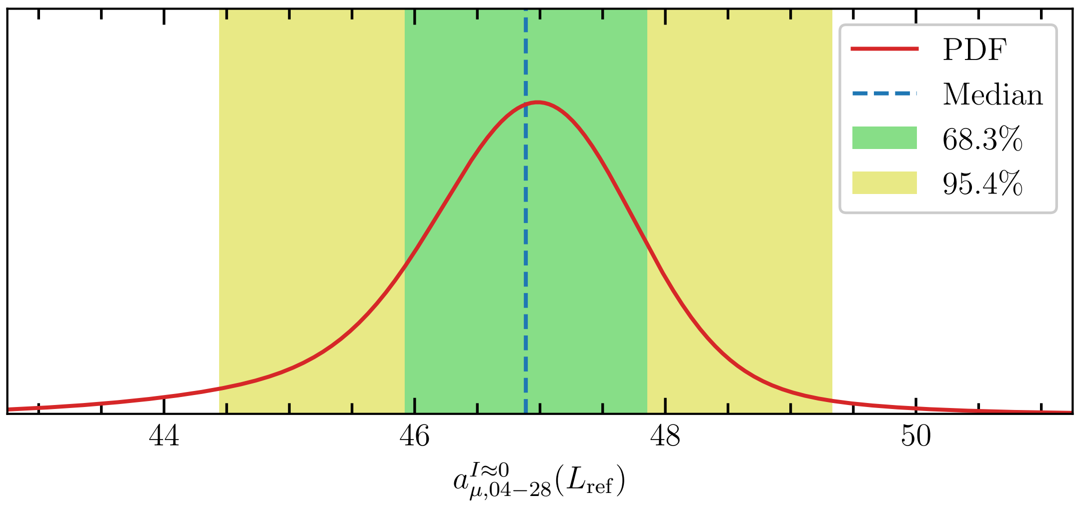

Number of fits	7680	
Fits with $P > 0.1$	100%	
Median	46.89	
Total error	1.22	2.61%
Statistical error	1.19	2.54%
Systematic error	0.28	0.59%
Pseudoscalar fits	0.01	0.02%
M_{ss} value	0.01	0.01%
w_0 value	0.17	0.36%
Lattice spacing cut	0.04	0.08%
Fit polynomial order	0.25	0.54%
Log corrections γ	0.01	0.02%

Table S14: $I \approx 0$ window observable $a^{I\approx 0}_{\mu,04-28}$. The plot conventions are described in the first part of Section S6.

S6.7 Long-distance window $10-\infty$ and comparison with 2020 result

The standard long-distance window starts at Euclidean time 1.0 fm and extends to infinity. It is a central observable of the averaging procedure of the Theory Initiative [3]. Here we provide a pure lattice computation of this quantity[12]. Results are given at the isospin symmetric point of the WP25 scheme defined in Equation (3.9) of the White Paper [3]. According to this reference, results in the WP25 and BMW schemes can be directly compared, without including matching factors, at the current level of precision.

We apply the bounding method, originally proposed in [135, 136] and described in detail in Section 13 of our 2020 work. Lattice data is used up to a Euclidean time of 4.0 fm, after which it is replaced either with zero (lower bound) or with a two-pion correlator (upper bound). Here, to reduce uncertainties, this bounding method is adapted to the situation in which the $10-\infty$ window is obtained as the sum of smaller windows, that is

$$a_{\mu,10-\infty}^{\text{light}} = a_{\mu,10-32}^{\text{light}} + a_{\mu,32-36}^{\text{light}} + a_{\mu,36-\infty}^{\text{light}}. \tag{S62}$$

Thus, the lower bound is implemented via

$$a_{\mu,36-\infty}^{\text{light}}|_{\text{lo}} = a_{\mu,36-40}^{\text{light}} \ , \tag{S63}$$

whereas for the upper bound, we consider

$$a_{\mu,36-\infty}^{\text{light}}|_{\text{up}} = a_{\mu,36-40}^{\text{light}} + \left(\frac{a_{\mu,39-40}^{\text{light}}}{a_{\mu,39-40}^{2-\text{pion}}}\right) a_{\mu,40-\infty}^{2-\text{pion}} \ . \tag{S64}$$

The contributions to a_μ labelled "2-pion" are built from a correlation function that falls off exponentially with a rate given by the energy of two non-interacting pions, each with one unit of momentum, i.e. the ground state in the $I=1$ channel.

In this approach, the $10-32$, $32-36$ and $36-\infty$ windows are fitted together, like the three contributions to the $04-28$ window in Section S6.6. And again, in the interval from 1.0 to 4.0 fm only lattice data are used and above 4.0 fm, these are replaced by the bounds described above.

Because the 2-pion correlator, used in the upper bound of Equation (S64), increases more slowly than the lattice correlator as Euclidean time is decreased, the ratio of the lattice to the 2-pion $39-40$ window, in parentheses in that equation, is larger than da_μ^{light}/dt at $t=4.0$ fm. In turn, this means that the upper bound of Equation (S64) is even weaker than the one obtained by equating $da_\mu^{2-\text{pion}}/dt$ to da_μ^{light}/dt at $t=4.0$ fm. We choose the former because it smooths out possible fluctuations in the lattice correlator around $t=4.0$ fm.

The lower and upper bounds for $a_{\mu,10-\infty}^{\text{light}}$, resulting from our procedure, are used as a two-point systematic in our histograms. Thus, our central value for the $10-\infty$ window corresponds approximately to their average, and their half-difference becomes a systematic uncertainty.

Our results are presented in Table S15. In the long-distance-window results collected in the current White Paper, finite-size corrections are obtained via phenomenology, not from a lattice calculation. For a fair comparison, we apply the same choice here. We use the data-driven determination from Table S24 and for the much smaller finite-time correction, NNLO XPT. For the latter, we obtain $\Delta a_\mu^{I=1}(L_{\text{ref}}, T_{\text{ref}} \to L_{\text{ref}}, \infty) = -0.47$. Adding these corrections to the lattice results, we obtain in infinite volume,

$$a_{\mu,10-\infty}^{\text{light}} = 404.6(2.7)(2.5)[3.7] \ . \tag{S65}$$

This number can be compared to the other lattice results [51, 52, 137], published while the present paper was being refereed. Those results were subsequently averaged by the Theory Initiative to provide a new recommended value for a_μ in the 2025 White Paper [3]. In Figure S14, we compare our result with those from the other groups, together with the White Paper average.

[12] We thank H. Wittig for discussions on this point.

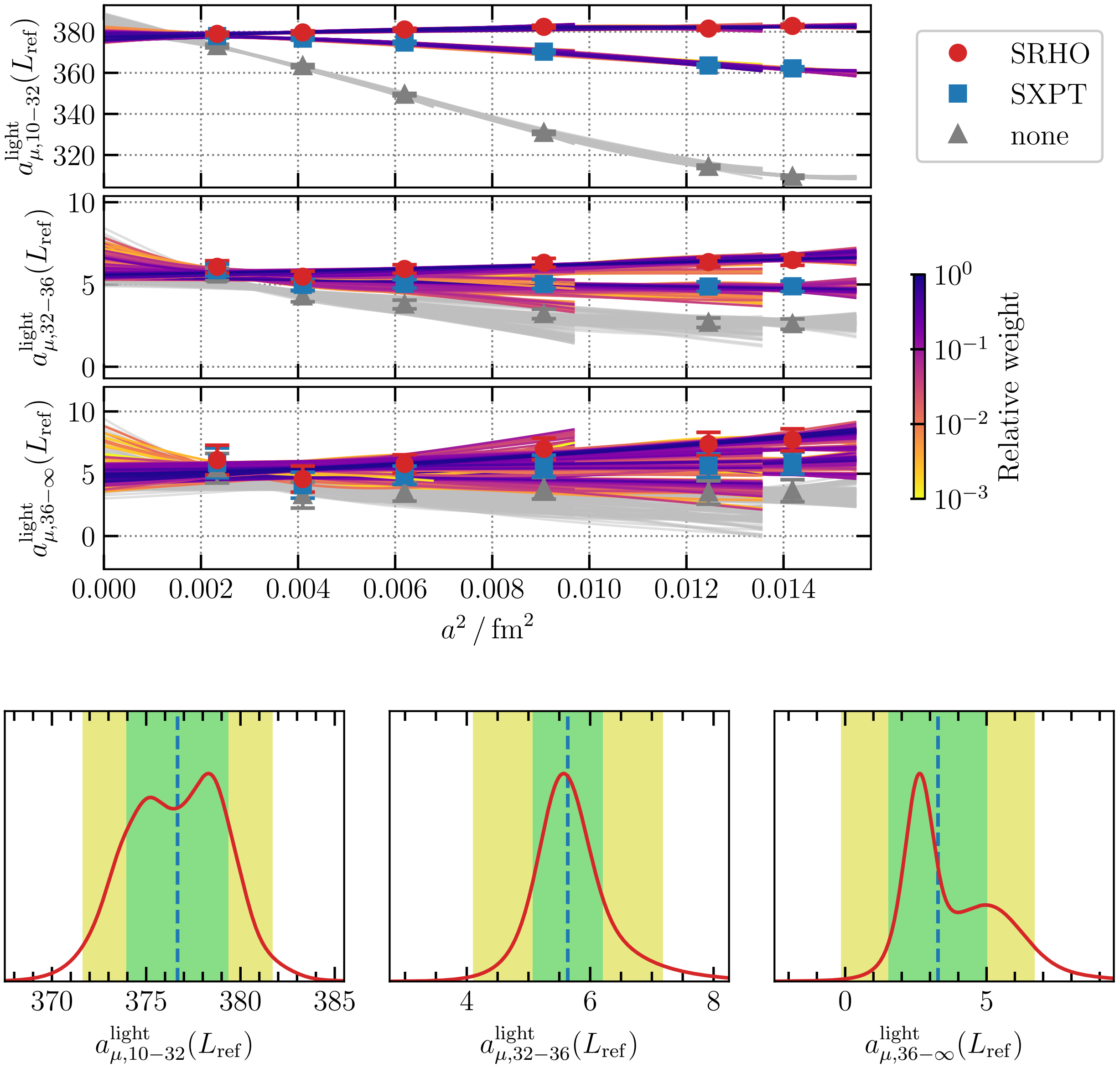

Number of fits	496793088							
Fits with $P > 0.1$	100%							
Median	386.06		376.66		5.64		3.28	
Total error	3.64	0.94%	2.70	0.72%	0.77	13.66%	1.75	53.22%
Statistical error	2.68	0.70%	1.47	0.39%	0.63	11.16%	0.97	29.51%
Systematic error	2.46	0.64%	2.27	0.60%	0.44	7.89%	1.45	44.29%
Pseudoscalar fits	0.06	0.02%	0.05	0.01%	0.02	0.28%	0.02	0.55%
Taste correction	1.49	0.39%	1.40	0.37%	0.13	2.33%	0.04	1.34%
Bounding	1.22	0.32%	0.00	0.00%	0.01	0.20%	1.21	36.93%
Lattice spacing cut	0.54	0.14%	0.53	0.14%	0.07	1.25%	0.11	3.38%
Fit polynomial order	0.98	0.25%	0.57	0.15%	0.31	5.54%	0.30	9.24%
Log corrections γ	0.64	0.16%	0.62	0.16%	0.03	0.61%	0.06	1.70%
	$10-\infty$		$10-32$		$32-36$		$36-\infty$	

Table S15: Light-connected window observable $a^{\text{light}}_{\mu,10-\infty}$ obtained as sum of three windows $10-32$, $32-36$ and $36-\infty$, fitted together. The plot conventions are described in the first part of Section S6. The continuum extrapolations are shown with no, NNLO XPT and SRHO taste improvements, and for the case of the upper bound. The two-peak structure in the histogram of $10-32$ and $36-\infty$ comes from the taste improvement variation and the bounding, respectively.

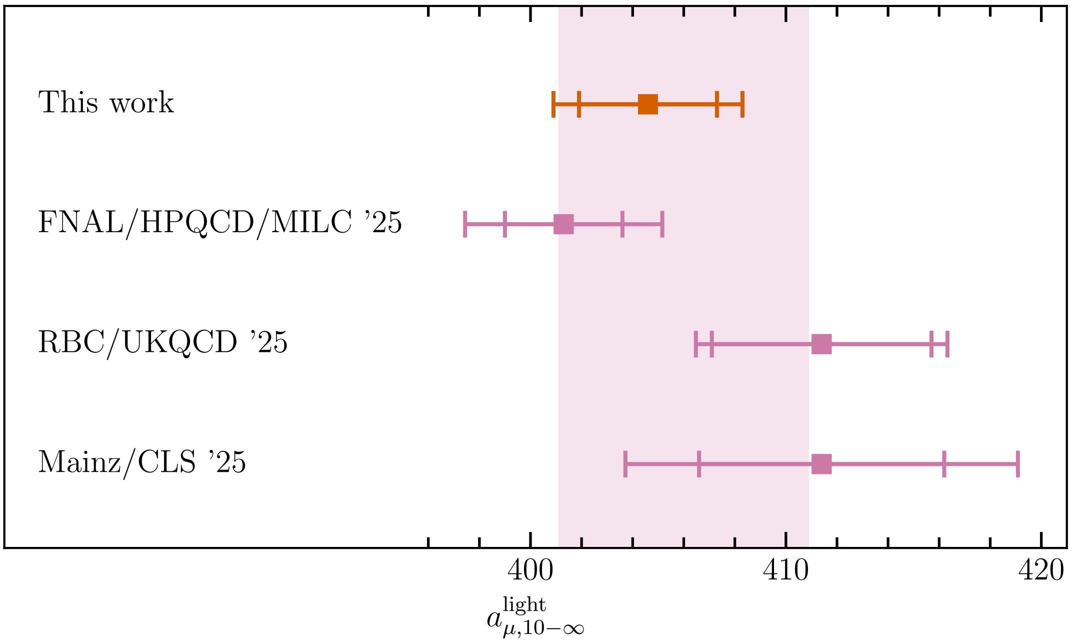

Figure S14: Comparison between different lattice results for the light connected long-distance window, $a_{\mu,10-\infty}^{\text{light}}$. The results are given in the WP25 scheme defined in Equation (3.9) of the White Paper [3]. The pink band is the average of all the lattice results excluding ours, as published in [3].

In what follows, we address three questions relevant to our analysis. First, we quantify the gain in precision achieved by including the finest lattice spacing. Second, we assess the improvement obtained when the lattice determination of the $28-\infty$ tail is replaced by our data-driven approach. Third, we examine how our current determination of the light-connected contribution differs from our earlier work. For this purpose, it is sufficient to restrict attention to results obtained in our reference volume. Our starting point is the $10-\infty$ window presented in Table S15, for which we obtain:

$$a_{\mu,10-\infty}^{\text{light}}(L_{\text{ref}}) = 386.1(2.7)(2.5)[3.6] \; . \tag{S66}$$

To answer the first question, we perform the analysis without the finest ensembles, i.e. those with lattice spacing $a = 0.048$ fm. We obtain

$$a_{\mu,10-\infty}^{\text{light}}(L_{\text{ref}}) = 383.2(4.0)(4.1)[5.7] \qquad \text{(without the finest lattice).} \tag{S67}$$

From this and the previous result, we conclude that the inclusion of the new lattice spacing reduces the error by $1-3.6/5.7 = 37\%$. Note that in our 2020 work, the error on the light-connected contribution, Equation (S70), is smaller than that of Equation (S66), obtained by including our finest lattices. The reason for this is that our current analysis is more conservative than the 2020 one. Here, we include fit functions that are cubic in a^2, whereas they only went up to quadratic order in our 2020 work. Moreover, the largest lattice spacing used here is only $a = 0.119$ fm, while previously it was $a = 0.132$ fm. There are also other differences in the analysis, e.g. we now use a multi-window fit procedure, whereas earlier we fitted the whole time range at once.

To answer the second question, we take the value of the $10-28$ window from Table S11:

$$a_{\mu,10-28}^{\text{light}}(L_{\text{ref}}) = 366.4(1.8)(2.2)[2.8] \; . \tag{S68}$$

Comparing its uncertainty with that of the result in Equation (S66), our hybrid approach—where the lattice determination of the $28-\infty$ tail is replaced by a data-driven estimate whose uncertainty can be neglected for the purposes of this exercise—achieves a $1-2.8/3.6 = 22\%$ improvement in precision

relative to a purely lattice-based computation employing the bounding method.

For addressing the third question, we combine our pure lattice determination for the $10-\infty$ window with the short- and intermediate-distance windows to get

$$a_\mu^{\rm light}(L_{\rm ref}) = 640.1(2.8)(2.5)[3.7] \ , \tag{S69}$$

which is larger than our 2020 result

$$a_\mu^{\rm light}(L_{\rm ref}) = 633.7(2.1)(4.2)[4.7] \qquad \text{(our 2020 estimate)} \tag{S70}$$

by 6.4 units. According to Equations (S66) and (S67), about 2.8 units come from the addition of a finer lattice spacing. An additional contribution of approximately 4.0 units arises from the fact that, in the nomenclature of Ref. [1], our 2020 result was obtained using a Type-I fit, in which the lattice scale is set directly using the mass of the Ω^- baryon, whereas the present result is based on Type-II fits, which employ the intermediate scale w_0. The size of this contribution is obtained directly from the difference between the results for the light-connected contribution from the Type-II and Type-I analyses, using the 2020 dataset. Any residual discrepancy can be attributed to the differences in the analysis procedures described above.

We can also give an estimate of the error on the difference between Equations (S69) and (S70), taking correlations into account. For the statistical correlation coefficient between the 2020 and current results, we obtain $r = 0.41$. To correlate the systematic errors, we use the error budgets from Table S15 of the present paper and from Table 14 of our previous work. The taste-correction uncertainty is treated as fully correlated. However, there is still a large residual uncertainty on the difference due to the significant reduction from 3.8 in our 2020 work to 1.5 in the current one. We assigned no uncertainty to the bounding method in 2020 (it was assumed to be covered by the statistical error), so the uncertainty of 1.2 that we assign this time is uncorrelated. The remaining systematic was 1.8 in 2020, whereas it is 1.5 now. As the two analyses are very different, one could argue for taking zero correlation between their remaining systematics. However, as another extreme, we also consider the case of full correlation. Assuming zero or full correlation, we obtain for the total uncertainty of the difference 4.5 or 3.8, respectively. This means that our new light connected value is 1.4σ or 1.7σ higher than the 2020 one, depending on the assumption made about the correlations in their remaining systematics.

S6.8 Strange and charm contributions

In addition to the light and disconnected parts of a_μ, we also performed an update of the strange and charm contributions. The procedure for the strange quark is the same as was used for the light. The main difference is that we split the strange contribution $a_{\mu,00-28}^{\rm strange}$ into only two windows, the short-distance $a_{\mu,00-04}^{\rm strange}$ and the remaining part $a_{\mu,04-28}^{\rm strange}$, which are fitted separately. We have 5632 independent fits for the strange short-distance contribution, and for both the remaining part and the intermediate window we have 2880 fits each. We obtain for the short-distance window

$$a_{\mu,00-04}^{\rm strange} = 9.04(3)(6)[7] \ , \tag{S71}$$

for the intermediate-distance window

$$a_{\mu,04-10}^{\rm strange} = 27.04(11)(8)[14] \ , \tag{S72}$$

and for a long-distance window

$$a_{\mu,10-28}^{\rm strange} = 16.89(9)(7)[11] \ , \tag{S73}$$

with the percentages of fits with P-value of at least 0.1 being 88%, 87% and 31%, respectively. Summing up the short-distance and the rest we get the contribution of the strange quark to a_μ:

$$a^{\rm strange}_{\mu,00-28} = 53.12(14)(11)[18] \ . \tag{S74}$$

The behaviour of the charm quark contribution to a_μ is different from the light, disconnected or strange contributions, and we modified the fit procedure accordingly. For the quark-mass mistuning in the charm sector we use the variable

$$X_c = M_{lc}^2 w_0^2 - \left[M_{lc}^2 w_0^2\right]_{\rm qcd} \tag{S75}$$

which describes the charm quark mass deviation of our ensembles from the physical point. The physical value of $M_{lc} = M_D = 1863.1(6)$ MeV in the isospin symmetric limit is taken from Ref. [108]. The global fit function then has the form

$$Y = A(a^2) + D(a^2)X_c \ , \tag{S76}$$

We used the same options for $A(a^2)$ as for the light, short-distance function given in Equation (S50). For the short distance part we also included tree-level corrected result and $Q/\hat{Q}$ variation in the fit procedure. In total we have 1408 fits for the charm short-distance contribution and 352 fits for the remaining part, with the percentages of fits with P-value above 0.1 being 81%, 47% and 27%, respectively. The final numbers for the individual windows together with the total result are

$$\begin{aligned} a^{\rm charm}_{\mu,00-04} &= 11.71(15)(21)[26] \ , \\ a^{\rm charm}_{\mu,04-10} &= 2.95(12)(17)[21] \ , \\ a^{\rm charm}_{\mu,10-28} &= 0.0142(17)(22)[28] \ , \\ a^{\rm charm}_{\mu,00-28} &= 14.68(24)(29)[38] \ . \end{aligned} \tag{S77}$$

S6.9 Other contributions, total and comparison with 2020 result

Until now we discussed the light, strange, charm and disconnected contributions to $a_{\mu,00-28}$, in the isospin-symmetric limit. The remaining ones are listed in the second part of Table S16, most of them are isospin-breaking corrections. Here we give details on how we have computed them.

In our 2020 work we computed isospin-breaking contributions for the total a_μ. In a previous version of this paper we took over the isospin-breaking contributions from our 2020 work as they are, instead of computing them in the $00-28$ window. We now remove this shortcoming. For this purpose we repeat the very same analysis as in our 2020 work for isospin breaking in the $a_{\mu,00-28}$ window, in the cases of the light, disconnected and strange flavours. These are called "Type-II" fits and are detailed in Section 24 of the 2020 paper [1]. The results are reported in Table S16. It turns out, that the numbers obtained in 2020 for the total range and the numbers obtained now for the $00-28$ window are in good agreement with each other. However, we consider the errors of the $00-28$ window quantities more reliable, since the long distance part can be very challenging to estimate.

Now we add up the light, disconnected, strange, charm and isospin-breaking contributions in the $00-28$ window determined on the lattice in this work, plus the remaining contributions from previous lattice computations. These are the first, second and third sections of Table S16. We are not correlating the isospin-breaking, the strange, and charm computations with each other or with the light plus disconnected part. We justify this choice by noting that these observables describe very different physics, each with its own kind of lattice artefact and in case of the isospin breaking sometimes they are measured on different sets of ensembles than the isospin symmetric ones. Finally we add the $28-\infty$ window from the data-driven approach discussed in Section S10. Altogether we get for the leading-order hadronic vacuum polarization contribution to the muon magnetic moment:

$$a_\mu = 715.1(2.5)(2.3)[3.4] \tag{S78}$$

$00-28$	light and disconnected	$619.6(2.3)(2.1)[3.1]$	this work, Equation (S61)
$00-28$	strange	$53.12(14)(11)[18]$	this work, Equation (S74)
$00-28$	charm	$14.68(24)(29)[38]$	this work, Equation (S77)
$00-28$	light qed	$-1.86(53)(27)$	this work
$00-28$	light sib	$7.06(64)(35)$	this work
$00-28$	disconnected qed	$-1.00(46)(28)$	this work
$00-28$	disconnected sib	$-4.39(58)(63)$	this work
$00-28$	strange qed	$-0.0136(85)(77)$	this work
$00-\infty$	disconnected charm	$0.0(1)$	[24], Section 4 in Supp. Mat.
$00-\infty$	charm qed	$0.0182(36)$	[138]
$00-\infty$	bottom	$0.271(37)$	[139]
$28-\infty$	tail from data-driven	$27.59(26)(45)[52]$	this work, Equation (S90)
$00-\infty$	total	$715.1(2.5)(2.3)[3.4]$	

Table S16: List of all contributions to a_μ.

$04-10$	light	$206.92(37)(34)[50]$	this work, Equation (S53)
$04-10$	disconnected	$-1.049(21)(24)[32]$	this work, Equation (S54)
$04-10$	strange	$27.04(11)(8)[14]$	this work, Equation (S72)
$04-10$	charm	$2.95(12)(17)[21]$	this work, Equation (S77)
$04-10$	light qed	$0.035(40)(44)$	[1], Table 17
$04-10$	light sib	$0.753(40)(16)$	[1], Table 17
$04-10$	disconnected qed	$-0.117(17)(6)$	[1], Table 17
$04-10$	disconnected sib	$-0.237(9)(6)$	[1], Table 17
$04-10$	strange qed	$-0.0050(35)(37)$	[1], Table 17
$04-10$	total	$236.29(41)(39)[57]$	

Table S17: List of all contributions to the intermediate-window $a_{\mu,04-10}$.

$10-28$	light and disconnected	$365.6(2.4)(2.0)[3.1]$	this work, Equation (S58)
$10-28$	strange	$16.89(9)(7)[11]$	this work, Equation (S73)
$10-28$	charm	$0.0142(17)(22)[28]$	this work, Equation (S77)
$10-28$	light qed	$-1.79(52)(29)$	this work
$10-28$	light sib	$6.24(64)(36)$	this work
$10-28$	disconnected qed	$-0.89(45)(29)$	this work
$10-28$	disconnected sib	$-4.13(57)(60)$	this work
$10-28$	strange qed	$-0.0094(55)(42)$	this work
$28-\infty$	tail from data-driven	$27.59(26)(45)[52]$	this work, Equation (S90)
$10-\infty$	total	$409.5(2.6)(2.2)[3.4]$	

Table S18: List of all contributions to the long-distance-window $a_{\mu,10-\infty}$.

with statistical, systematic and total errors. This is the main result of this work.

This result differs from our 2020 determination by 7.6 units (see also the discussion in Subsection S6.7). We next address whether this difference is statistically significant. We begin by outlining, in general terms, the various sources of correlation between our current and previous analyses, before quantifying their impact below.

Statistical correlations between our 2020 analysis and the present work can be computed using jackknife resampling, and we find them to be small. This dilution arises both from the inclusion of a new lattice spacing and from our use of the $a_{\mu,00\text{–}28}$ window rather than a_μ.

In addition, the dominant source of systematic uncertainty differs markedly between the two studies. In 2020, the leading systematic error originated from the difference between the SRHO and NNLO taste-improvement procedures. That uncertainty is now significantly smaller, owing to the availability of finer lattices and to our decomposition of the analysis into multiple windows. This uncertainty is fully correlated, but due to its reduction in the present work, there is still a large residual uncertainty on the difference.

In the present work, the dominant systematic uncertainty instead stems from the scale-setting quantity w_0, whereas the 2020 analysis relied on M_Ω for scale setting. Because neither the uncertainties associated with M_Ω nor those in a_μ are expected to correlate with fluctuations in w_0, it is reasonable to treat these sources of systematic as uncorrelated.

Regarding finite-size corrections, we assume full correlation between the 2020 and present analyses, since these corrections were obtained from the same `4hex` configurations and were applied to a_μ in 2020 and to $a_{\mu,00\text{–}28}$ here.

By contrast, the continuum-extrapolation uncertainties in the 2020 and 2025 analyses are expected to be largely uncorrelated, given the substantial methodological differences in how each is obtained. In 2020, the continuum limit was obtained from a single fit of a_μ, whereas in the present work $a_{\mu,00\text{–}28}$ is decomposed into four windows, each with its own fit function and lattice-spacing cuts. This methodological difference provides a reasonable basis for treating the associated continuum-extrapolation uncertainties as uncorrelated. Nevertheless, for completeness, we also consider the opposite extreme of full correlation. The same treatment is applied to the remaining systematic uncertainties, such as those associated with the fit ranges for the pseudoscalar masses, etc.

Accounting for the correlations discussed above, we estimate the uncertainty on the difference between the 2020 and present results. The statistical correlation coefficient is $r = 0.16$. The taste-improvement systematic was 3.8 in 2020 and 0.9 in the current analysis; assuming full correlation, the uncertainty on the difference is 2.8. Continuum-extrapolation and remaining systematic uncertainties remain essentially unchanged at 1.9.

For finite-size corrections, treated as fully correlated and using the `4hex` simulations, the uncertainty on the difference is also 1.9, arising from the finite volume corrections beyond 2.8 fm that do not enter into the hybrid result.

Combining all contributions in quadrature, and considering both zero and full correlation for the continuum-extrapolation and remaining systematics, we obtain total uncertainties of 5.2 and 4.5, respectively. Thus, the new result exceeds the 2020 value by 1.5σ or 1.7σ, in agreement with the difference observed for the light-connected contribution in Section S6.7.

We can also give a complete result for the intermediate-window observable. The light and disconnected contributions are given in Equations (S53) and (S54). The others were determined in our previous work, which we give here for completeness in Table S17. Adding up all these contributions we obtain the value

$$a_{\mu,04-10} = 236.29(41)(39)[57] \ , \tag{S79}$$

which is compared to other determinations, lattice and data-driven, in Figure 2 of the main article.

We further provide a complete result for the long-distance window observable, where the Euclidean time range goes from 1.0 fm to infinity. The largest contribution is given by the light flavour in the $10-28$ window, whose analysis details can be found in Table S11. Adding the disconnected contribution gives the result in Equation (S58). We performed analyses for all the other contributions as in the case of the

$00-28$ window, with the results listed in Table S18. The $28-\infty$ window is taken from the data-driven approach again. Adding all of those contributions up, we obtain

$$a_{\mu,10-\infty} = 409.5(2.6)(2.2)[3.4] \ . \tag{S80}$$

S7 Finite-size effects

Finite spatial and temporal box sizes cause a significant distortion of the target observables. We remove this by the same procedure as applied in our 2020 work [1]. The largest contribution comes from the finite-size effects in the isospin-symmetric part in the $I=1$ channel. The finite-size effects in the $I=0$ channel are much smaller and even smaller are those in the isospin-breaking part. Our procedure defines two box-sizes, the reference box size $L_{\rm ref}=6.272$ fm and a large box size $L_{\rm big}=10.752$ fm.

To compute the $I=1$ finite-volume effects in our 2020 work we prepared a dedicated set of simulations, the `4hex` data-set. We designed it to be sensitive to finite-size effects even in the presence of staggered taste violations. For this purpose we utilized a new staggered action, the action details can be found in our 2020 publication. We lowered the Goldstone-pion masses to $M_\pi=110$ MeV, so that the average of the masses of the pion taste multiplet equals the physical pion mass. We performed simulations with two different lattice geometries, one on a 56×84 and another on a large 96×96, which have spatial sizes $L_{\rm ref}$ and $L_{\rm big}$. For each case the simulations were performed using two different Goldstone-pion masses, $M_\pi=104$ MeV and 121 MeV. These were used to interpolate to the physical point defined above. The simulations and measurements were already available in our 2020 work, in Table S19 we give results for $a_\mu^{\rm light}$ in the different windows that are used in this paper.

In addition, we also utilized chiral perturbation theory (XPT), and its staggered version (SXPT), to compute finite-size effects [1, 25]. Values obtained for several different windows are given for $L_{\rm ref}$ and $L_{\rm big}$ box sizes, both with next-to-leading-order (NLO) and next-to-next-to-leading order (NNLO) chiral perturbation theory, in Table S20.

The `4hex` lattice data and chiral perturbation theory are combined in the following three step procedure to obtain our result in infinite volume:

1. On each `4stout` ensemble we apply a shift on the observables which brings them from the simulation box size to $L_{\rm ref}$. These shifts are computed with NNLO SXPT using the taste-violation of the ensemble as input. We then perform a continuum extrapolation, which then corresponds to a box size of $L_{\rm ref}$.

2. We add the difference in the observables between box sizes $L_{\rm big}$ and $L_{\rm ref}$ computed with the `4hex` simulation.

3. We add the difference between the infinite and $\rm big$ box sizes computed in NNLO XPT, also including corrections for the finite-time extent.

The `4hex` finite-size corrections in step 2 are obtained from the results in Table S19. We take into account lattice artefacts in the same way as in our 2020 work: we reduce the measured finite-size effect by 7%, and assign a 7% uncertainty to this correction step. The size of this correction step was taken from the deviation of the total a_μ light from its continuum value. The results obtained are shown in the second column of Table S21. They are computed from the $a_\mu^{\rm light}$ numbers from Table S19 including a multiplication by the $\left(\frac{9}{10}\right)$ charge factor. The first error is statistical and the second is an estimate of the cutoff effect. We can also compare the $L_{\rm ref}\to L_{\rm big}$ finite-size effect from the `4hex` simulations with the prediction of XPT, the corresponding numbers can be found in Table S20, both for NLO and NNLO XPT. We find a good agreement between the values of the `4hex` simulations and those of NNLO XPT.

Regarding step 3, which is the very small, residual finite-volume effect of the large box, we employ NNLO XPT. Here we can use the numbers from the last column of Table S20. We can estimate the size

window	$L \times T$	$M_\pi = 104$ MeV	$M_\pi = 121$ MeV	$M_\pi = 110$ MeV
$00-28$	56×84	656.77(72)	641.77(60)	651.35(49)
	96×96	669.39(55)	649.02(48)	662.03(43)
$00-04$	56×84	42.84(00)	42.85(00)	42.84(00)
	96×96	42.87(00)	42.87(00)	42.87(00)
$04-10$	56×84	207.53(03)	206.54(04)	207.17(02)
	96×96	208.19(02)	206.96(02)	207.75(01)
$10-28$	56×84	406.40(72)	392.38(59)	401.33(48)
	96×96	418.33(55)	399.19(47)	411.41(43)
$10-\infty$	56×84	436.22(2.87)	419.15(2.16)	430.05(2.04)
	96×96	459.72(1.94)	434.22(1.73)	450.50(1.40)
$15-19$	56×84	107.83(15)	103.79(14)	106.37(11)
	96×96	110.32(10)	105.27(11)	108.50(08)
$28-35$	56×84	20.31(79)	18.60(52)	19.69(54)
	96×96	25.31(33)	21.50(42)	23.93(28)

Table S19: Results for a_μ^{light} in different windows for our 4hex simulations. These are performed with $M_\pi = 104$ MeV and 121 MeV Goldstone-pion masses. The last column contains values interpolated to the Goldstone-pion mass of $M_\pi = 110$ MeV, where the taste-averaged pion mass takes on the π^0's physical, mass value.

	$\Delta a_\mu^{I=1}(L_{\text{ref}} \to \infty)$		$\Delta a_\mu^{I=1}(L_{\text{big}} \to \infty)$	
window	NLO	NNLO	NLO	NNLO
$00-28$	6.90	8.97	0.39	0.43
$00-04$	0.01	0.02	0.00	0.00
$04-10$	0.33	0.40	0.01	0.01
$10-28$	6.56	8.55	0.37	0.42
$10-\infty$	12.02	16.33	1.18	1.40
$15-19$	1.48	1.87	0.07	0.07
$28-35$	2.46	3.45	0.25	0.29

Table S20: Finite-volume effects in the $I = 1$ channel computed in chiral perturbation theory. Results are shown for two different box sizes, L_{ref} and L_{big}, and for two different orders, NLO and NNLO.

window	$\Delta a_\mu^{I=1}(L_{\text{ref}} \to L_{\text{big}})$ from 4hex	$\Delta a_\mu^{I=1}(L_{\text{big}} \to \infty)$ from NNLO	$\Delta a_\mu^{I=1}(L_{\text{ref}} \to \infty)$ from 4hex+NNLO	$\Delta a_\mu^{I=0}(L_{\text{ref}} \to \infty)$ from N4LO est.
$00-28$	8.94(57)(67)	$+0.43-0.06$	9.31(88)	0.00(19)
$00-04$	0.02(00)(00)	$+0.00-0.00$	0.02(00)	0.00(01)
$04-10$	0.48(02)(04)	$+0.01-0.00$	0.49(04)	0.00(00)
$10-28$	8.44(57)(64)	$+0.42-0.06$	8.80(85)	0.00(18)
$10-\infty$	17.12(2.08)(1.29)	$+1.40-0.84$	17.68(2.45)	0.00(55)
$15-19$	1.78(12)(13)	$+0.07-0.00$	1.85(18)	0.00(03)
$28-35$	3.55(50)(27)	$+0.29-0.11$	3.73(57)	0.00(16)

Table S21: Finite-size effects on a_μ in different windows. The second column gives the difference in $a_\mu^{I=1}$ between boxes big and ref computed with the 4hex simulations. The third column gives our NNLO XPT computations for the finite-size effects of the big box: the first/second number corresponds to the finite-volume/finite-time correction. The fourth is the sum of the second and third columns with errors added in quadrature. The last column is an estimate of the finite-size effect in the $I = 0$ channel from N4LO XPT.

of the neglected N3LO contribution[13], using the the NLO and NNLO values in the Table. It turns out to be an order of magnitude smaller than the total error in our `4hex` data set, so the NNLO approximation used here is sufficient. We also performed these XPT computations taking into account a finite temporal extent. The relevant formulae can be found in our 2020 paper. These finite-time effects are even smaller than the residual finite-volume effects. Both the finite-volume and the finite-time effects are listed in the third column of Table S21.

There is also a finite-size effect in the $I = 0$ channel. We expect it to be N4LO, as discussed in our 2020 work. In the fifth column of Table S21, we give our estimates for the N4LO contribution in the different windows. Those numbers are obtained from the NLO and NNLO values in Table S20. The estimates are taken to be an additional systematic error in the final result. There are also finite-size effects in the isospin-breaking contributions, for which our 2020 estimate was ± 0.10 in the total a_μ. In the windows these effects have to be proportionally smaller and can be safely neglected at the current level of precision.

To summarize, the finite-size effect in the L_{ref} box size can be obtained by adding the $I = 1$ and $I = 0$ contributions from the last two columns of Table S21. The light and disconnected cases can be corrected from the values in the Table according to

$$\Delta a_\mu^{\mathrm{light}} = \tfrac{10}{9}\Delta a_\mu^{I=1} \quad \text{and} \quad \Delta a_\mu^{\mathrm{disc}} = -\tfrac{1}{9}\Delta a_\mu^{I=1} + \Delta a_\mu^{I=0} \; . \tag{S81}$$

These numbers have to be added to the continuum-extrapolated results of the `4stout` data-set, which correspond to L_{ref} box size.

S8 Data-driven checks of finite-volume corrections

Using a data-driven approach, in this section we provide an alternative evaluation of the finite-volume corrections to the various isovector window observables considered in this paper. This serves as a cross-check for the corresponding lattice calculation based on the `4hex` ensembles, and despite being an interesting result on its own, it is not used in our final evaluation. We generically denote these finite-volume corrections as $\Delta a_\mu^{I=1}(L_{\mathrm{ref}} \to \infty)$.

We employ a combination of the Meyer-Lellouch-Lüscher [26–28] (MLL) and the Hansen-Patella [29, 30] (HP) methods. They allow the calculation of finite-volume corrections from the infinite-volume $\pi\pi$ elastic phase shift δ_{11} in the $I^G(J^{PC}) = 1^+(1^{--})$ channel. The methods additionally require the $I = 1$ component of the pion electromagnetic form factor $F_\pi^{I=1}(s)$, evaluated in the timelike domain $s > 0$ for MLL and in the spacelike region $s \leq 0$ for HP.

The MLL formalism has been widely used with the Gounaris-Sakurai parametrization [140] of the form factor and the phase shift. We adopt instead a data-driven approach based on the parametrization of Ref. [13, 141, 142]. The corresponding parameters are obtained via new individual fits to three measurements of the $e^+e^- \to \pi^+\pi^-$ spectra by BaBar [15, 16], CMD-3 [2] and KLOE [17–20], according to the procedure described in Ref. [13]. For each data set we then obtain the phase shift $\delta_{11}(s)$ and the form factor,

$$F_\pi^{I=1}(s) = J(s)\Omega(s) \; , \tag{S82}$$

where $\Omega(s)$ is the Omnès function [143],

$$\Omega(s) = \exp \frac{s}{\pi} \int_{s_{\mathrm{th}}}^{\infty} ds' \, \frac{\delta_{11}(s')}{s'(s'-s)} \; , \tag{S83}$$

and $J(s) = 1 + \alpha_V s$, where α_V is one of the fit parameters. Equation (S82) corresponds to the $I = 1$ component of the pion form factor, up to inelastic contributions modelled by $J(s)$.

Following Ref. [1], for MLL eight finite-volume states are used to construct the finite-volume correlator

[13] We use N3LO for next-to-next-to-next-to-leading order and the N4LO for the subsequent one.

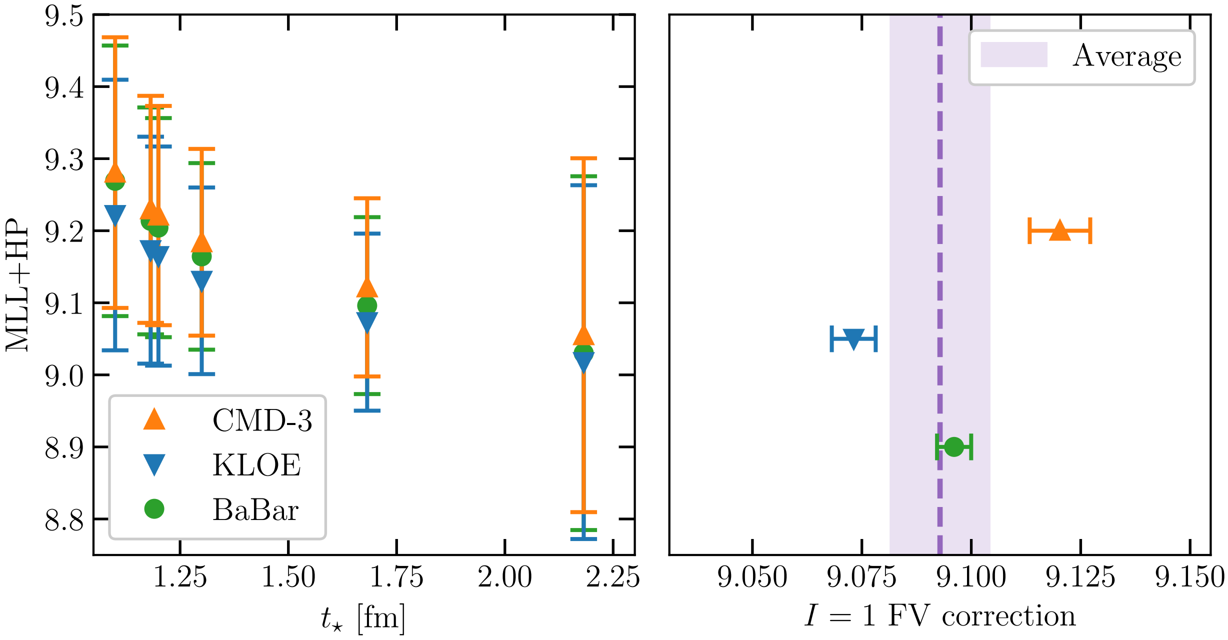

Figure S15: Results for a data-driven determination of finite-volume effects in the $00-28$ window. The left panel shows, on the y-axis, the sum of the MLL and HP (1-pion and regular) contributions to the $I=1$ finite-volume correction, obtained using fits to the different experimental data sets, as a function of the time $t_\star$ at which we switch from HP to MLL. In addition to the small error due to the fit parameters, the uncertainty reported here accounts for the truncation of either series and for the one associated with the regular part of HP, as described in the main text. The right panel contains again $I=1$ finite-volume corrections from individual data sets, but the uncertainty comes solely from the fit parameters that determine the phase shift. On the same plot the weighted average is shown as a purple dashed vertical line, the purple band corresponds to its uncertainty multiplied by a factor of $\sqrt{\chi^2/\mathrm{dof}}$, as described in the text.

$G(t,L)$, an approximation that is increasingly more accurate at larger Euclidean times t. The last, eighth term is taken as an estimate of the uncertainty due to the truncation of the sum over states. It is added to the finite-volume correlator with a 100% uncertainty. To keep this uncertainty small and its estimate reliable, we use MLL only at times larger than $t_\star = (M_\pi L/4)^2/M_\pi$, as suggested in Ref. [144].

Below $t_\star$, where MLL poorly represents the finite-volume correlator, the HP series of Ref. [30] is used, including the three terms that have been computed, thus neglecting contributions of order $\exp\left(-M_\pi L\sqrt{2+\sqrt{3}}\right)$ and higher. The last, third term is used as an estimate of the uncertainty due to the truncation of the series. The calculation of these terms requires knowledge of the Compton scattering amplitude of a pion off a spacelike photon. As shown in Ref. [30] this is dominated by the exchange of a single pion. This is in turn related to the spacelike pion form factor, for which we use again Equation (S83). The result for the HP single pion term is then added to the MLL contribution. Their uncertainties are summed assuming 100% correlation, since they derive from the same phase shift.

The rest of the Compton amplitude was computed in NLO XPT in Ref. [30]. We adopt the same expression, which amounts to a small contribution to the result, called "regular part", to which we assign a 100% uncertainty that is combined in quadrature with the other uncertainties. Results for the combination of MLL and HP are listed in Table S22 for the windows of interest in this paper.

The choice of $t_\star$ should not be critical as long as one does not use MLL at short distances, or HP at long distances, where the methods are not reliable. We have checked the dependence of our result on different values of $t_\star$. We find compatibility within the uncertainties, provided we do not consider large or small values of $t_\star$, as shown in the left panel of Figure S15 where we scan around $t_\star = 1.682\,\mathrm{fm}$, using

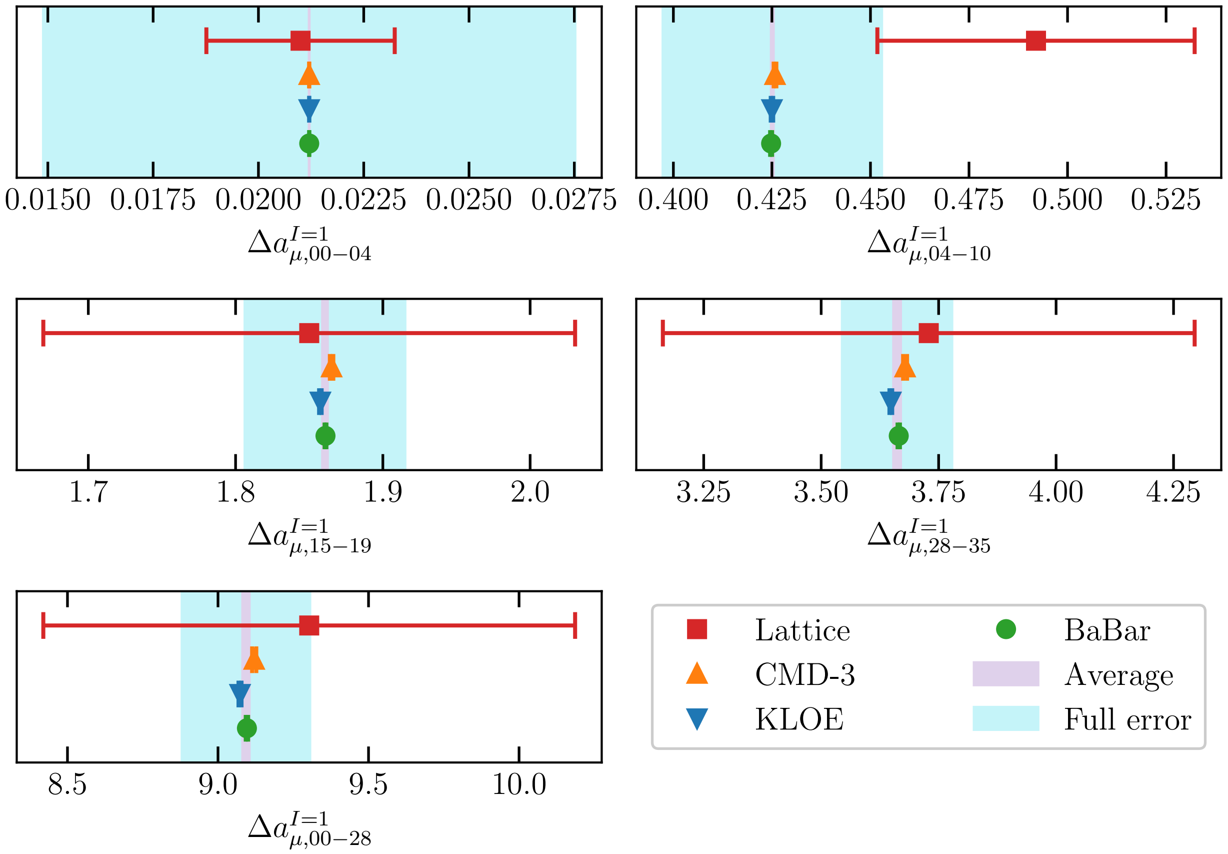

Figure S16: Comparison of results for the $I = 1$ finite-volume correction in various windows. The red points correspond to the `4hex` lattice results. The other points correspond to the data-driven determinations from individual data sets. Their averages are also displayed (inner purple band), as described in Figure S15. Finally, the outer cyan band displays the total uncertainty on our data-driven determination, accounting for the systematic effects described in this section.

the $00-28$ window as an example.

Another potential source of systematic uncertainty is the inelastic contribution to $F_\pi^{I=1}(s)$ due to four-pion states. Given that inelasticities become sizeable only at energies around $M_\omega + M_\pi$ [145], one would expect them not to propagate significantly into the finite-volume correction to a_μ. We note that XPT predicts that they begin at order s^3. In the chiral regime, s^3 is two orders higher than the linear s term in $J(s)$. Thus we conservatively take the effect of that linear term as an estimate of the possible size of such higher-order contributions. This is achieved by repeating our calculation of the finite-volume corrections without that linear term in $J(s)$. This results in a small shift in the finite-volume corrections, which we take as an additional systematic.

While we use the phase shift in the isospin limit, where we identify the pion mass with M_{π^0}, our parametrization of the phase shift is fitted to experimental data, where the pions are charged. We regard this as an isospin-breaking effect in the finite-volume corrections. In order to estimate this effect, we adopt the two-loop inverse-amplitude-method (IAM) expression of $\delta_{11}(s)$ from Ref. [146], which has the advantage of displaying dependence on the pion mass. By using the inputs for the IAM provided in Ref. [145, 147], we perform our calculation with the phase shift at the mass of neutral and of charged pions. The resulting shifts in the finite-volume corrections are taken as estimates of this additional source of uncertainty. We consider this effect to be independent of the other systematics and we add it to them in quadrature.

Finally, we need to propagate the uncertainties from the fits to BaBar, KLOE and CMD-3. To this end we generate Gaussian samples for the fit parameters associated with each experimental data set. While

the parameters associated with different experiments are not correlated, they are correlated for a given data set. We account for those correlations and compute the standard deviations on the resulting finite-volume corrections. Since the data sets are independent, we compute an average of the corresponding finite-volume corrections, each weighted by its uncertainty. The result is reported in the right panel of Figure S15, where the uncertainty has been rescaled by a factor of $\sqrt{\chi^2/\mathrm{dof}}$. With this procedure, before including the various other systematic uncertainties described above, the finite-volume correction determined from CMD-3 data is not compatible with the one coming from fits to BaBar and KLOE data. This is because the phase shift (the input to this calculation) is obtained by global fits that extend up to ~ 1 GeV, thus including the region dominated by the ρ peak, where discrepancies between experiments are observed. As we will see later, this effect is much smaller than the other systematics of the calculation. The same applies for including experiments, other than the above three: they would not significantly impact the final result and error for the finite-volume effect.

The various sources of uncertainties on the finite-volume corrections studied here are listed in Table S23: truncation, regular part of HP, inelastic, tension between experiments and isospin breaking. They are assumed to be independent and are summed in quadrature. Truncation, inelastic and isospin uncertainties are computed for each data set and we only keep the largest value for each source of uncertainty. The result of this combination is shown for each window in Table S23, which contains our complete data-driven determinations of the finite-volume corrections. We find them to be in agreement with the corresponding values computed using the 4hex simulations. In Figure S16 we update the right panel of Figure S15 to include the full uncertainty (cyan band) and a comparison with the corresponding 4hex value, and we display results for all windows.

In Table S24 we compare finite-volume effects as computed in the data-driven approach in this section to those computed in NNLO XPT and in the 4hex simulations, as described in the previous section. We find a good agreement between the various approaches. In our final results the bulk of the finite-size correction is taken from the 4hex simulations, and for a residual finite-size effect we use NNLO XPT.

S9 Verification of isospin-breaking contributions

As already mentioned in Section S6, we obtain the isospin-breaking (IB) contributions to $a_{\mu,00-28}$ by repeating the same analysis as in our 2020 work [1], now in the $00-28$ window. In order to further validate our previous procedures, we have performed several additional checks.

S9.1 Isospin breaking contributions to a_μ^{light}

In the present subsection we revisit the computation of the strong-isospin-breaking (SIB) and the valence QED contributions to a_μ^{light}, obtained with the derivatives

$$[a_\mu^{\mathrm{light}}]'_m \equiv m_l \left.\frac{\partial [a_\mu^{\mathrm{light}}]}{\partial\,\delta m}\right|_{\delta m=0} \quad \text{and} \quad [a_\mu^{\mathrm{light}}]''_{20} \equiv \frac{1}{2}\left.\frac{\partial^2 [a_\mu^{\mathrm{light}}]}{\partial e_v^2}\right|_{e_v=0} . \tag{S84}$$

Here $\delta m \equiv m_d - m_u$ denotes the difference in the down and up quark masses, $m_l \equiv \frac{1}{2}(m_u + m_d)$ denotes their average, and e_v denotes the valence electric charge.

In our work [1], these contributions were obtained using a chiral extrapolation: we performed computations with valence quark masses that were multiples κ of the sea quark mass, m_l, then we used the measurements at the values $\kappa = 3, 5, 7$ to perform an extrapolation to the physical point of $\kappa = 1$ gauge-configuration by gauge-configuration. Here we check the validity of this procedure by eliminating this extrapolation and by performing measurements directly at $\kappa = 1$.

In order to compute the SIB contribution $[a_\mu^{\mathrm{light}}]'_m$, we have extended the Low Mode Averaging (LMA) technique [148–151], which we were already using to reduce the noise in the isospin-symmetric contribution. We have performed measurements at $\kappa = 1$ on the same ensembles that were previously used for the

window	MLL	HP(1-pion)	HP(regular)	sum
$00-28$	$6.60(0.02)$	$2.43(0.09)$	$0.06(0.06)$	$9.09(0.12)$
$00-04$	$0.00(0.00)$	$0.01(0.00)$	$0.01(0.01)$	$0.02(0.01)$
$04-10$	$0.00(0.00)$	$0.40(0.01)$	$0.02(0.02)$	$0.43(0.03)$
$10-28$	$6.60(0.02)$	$2.02(0.08)$	$0.03(0.03)$	$8.65(0.11)$
$10-\infty$	$15.11(0.02)$	$2.02(0.08)$	$0.03(0.03)$	$17.16(0.11)$
$15-19$	$1.13(0.01)$	$0.72(0.03)$	$0.01(0.01)$	$1.86(0.04)$
$28-35$	$3.66(0.00)$	$0.00(0.00)$	$0.00(0.00)$	$3.66(0.00)$

Table S22: Results for the HP and MLL contributions in the windows of interest in this paper. HP is used to compute the finite-volume corrections to $a_\mu^{I=1}$, integrating the correlator from $t = 0$ up to $t_\star = 1.682\,\mathrm{fm}$. It is divided into the single pion contribution, reported in the third column, and the regular part, in the fourth column. From $t_\star$ to infinity, MLL is used. The uncertainty on the sum, reported in the last column, assumes 100% correlation between the uncertainties on MLL and HP (1-pion). The uncertainty attached to the regular part of HP is added in quadrature.

window	trunc. (MLL+HP)	HP(regular)	inel.	exp.	iso.	quad. sum
$00-28$	0.11	0.06	0.14	0.01	0.10	0.21
$00-04$	0.00	0.01	0.00	0.00	0.00	0.01
$04-10$	0.01	0.02	0.00	0.00	0.01	0.03
$10-28$	0.10	0.03	0.14	0.01	0.09	0.20
$10-\infty$	0.10	0.03	0.42	0.04	0.26	0.51
$15-19$	0.04	0.01	0.03	0.00	0.02	0.05
$28-35$	0.00	0.00	0.10	0.01	0.06	0.12

Table S23: Uncertainties in the finite-volume corrections to $a_\mu^{I=1}$ in the windows of interest in this paper. The numbers reported correspond to the data set (BaBar, KLOE or CMD-3) that has the largest uncertainty for the given entry. The second column is the sum of the truncation uncertainties of HP and MLL, which are assumed to be 100% correlated. The third column is due to the 100% uncertainty on the leftover regular part of HP. The fourth column is the difference between the finite-volume correction result, with and without the inelastic contribution to the pion form factor. The fifth column shows the error associated with the weighted average over experiments, inflated by $\sqrt{\chi^2/\mathrm{dof}}$. In the sixth column, we report an estimate of isospin-breaking effects. The last column contains the sum in quadrature of all these uncertainties.

window	data-driven	NNLO XPT	4hex + NNLO XPT
$00-28$	$9.09(0.21)$	8.97	$9.37(88)$
$00-04$	$0.02(0.01)$	0.02	$0.02(00)$
$04-10$	$0.43(0.03)$	0.40	$0.49(04)$
$10-28$	$8.65(0.20)$	8.55	$8.86(85)$
$10-\infty$	$17.16(0.51)$	16.33	$18.52(2.45)$
$15-19$	$1.86(0.05)$	1.87	$1.85(18)$
$28-35$	$3.66(0.12)$	3.45	$3.84(57)$

Table S24: Results for the finite-volume correction to $a_\mu^{I=1}$ in the windows of interest in this paper, using three different approaches: the data-driven approach, the pure NNLO XPT approach and 4hex direct measurements supplemented by NNLO XPT for residual effects. The figures of the last two are taken over from Tables S20 and S21 and they correspond to an infinite-time extent.

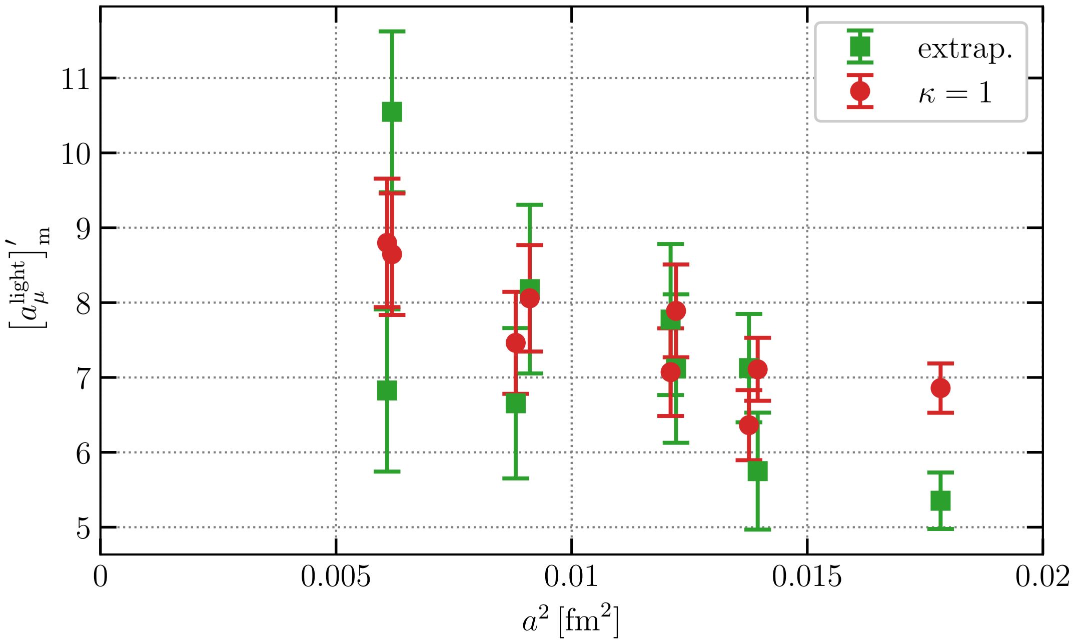

Figure S17: Strong-isospin-breaking (SIB) contribution to $a_\mu^{\rm light}$. The green squares show the results from [1], obtained via a chiral extrapolation from measurements performed at valence quark masses which are $\kappa = 3, 5, 7$ times larger than the physical light-quark mass m_l. The red circles correspond to the measurements performed directly at $\kappa = 1$, using an LMA-based technique.

κ extrapolation procedure. The new computation confirms the results of [1] within error-bars (cf. Figure S17).

For the valence QED contribution $[a_\mu^{\rm light}]''_{20}$, we have checked the gauge-configuration by gauge-configuration extrapolation procedure on one selected configuration. We have randomly chosen one QCD and QED configuration from one of our $a = 0.0787\,{\rm fm}$ ensembles, and measured the valence QED contribution using the LMA technique. For the exact eigen part we projected out 4128 eigenvectors, and used 36000 random source vectors for the $\kappa = 3, 5, 7$ measurements, and 72000 random source vectors for the $\kappa = 1$ measurement. Figure S18 shows $[a_\mu^{\rm light}]''_{20}$ as a function of the time cut t_c, the upper limit of the integration of the Euclidean time current-current correlator.

S9.2 Strong-isospin-breaking contribution to $a_\mu^{\rm disc}$

In our previous work [1] the SIB contribution to $a_\mu^{\rm disc}$ was computed by performing a numerical derivative: the mass derivative was approximated by the difference of the measurements at the light quark masses $1.0 \cdot m_l$ and $0.9 \cdot m_l$. Using the recently developed frequency-splitting estimator (FSE) technique of Ref. [152], we have remeasured on many of our ensembles both the disconnected contribution to a_μ, and its SIB correction as an exact mass derivative. By using the FSE method, the number of required random source vectors drops by one to two orders of magnitude. A comparison of the new FSE measurements of $[a_\mu^{\rm disc}]'_m$ and the results from [1] is shown in Figure S19.

S9.3 Corrections

We discovered two mistakes in our 2020 work, which affect the isopsin-breaking contributions. None of them was numerically important for a_μ, in the sense that the associated changes are far less than our final uncertainty.

First we set too aggressive time-cuts for the light-quark sea-QED contribution, $[a_\mu^{\rm light}]''_{02}$. We used 1.0 fm and 1.5 fm, at which point the corresponding correlator is not yet consistent with zero. An

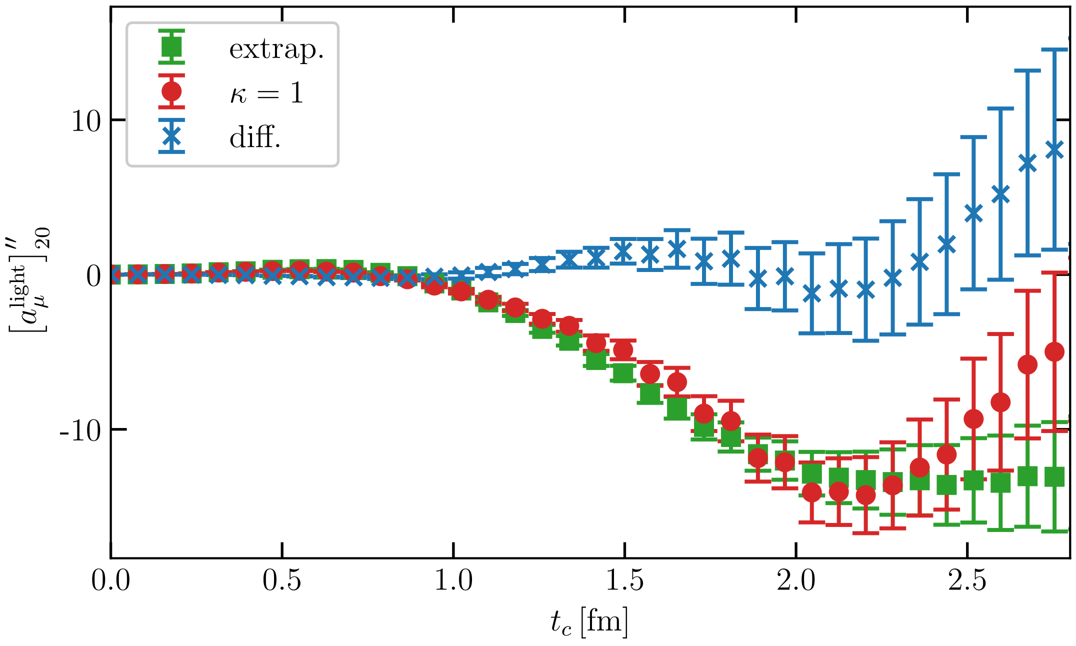

Figure S18: The valence QED contribution $[a_\mu^{\rm light}]''_{20}$ on a selected $a = 0.0787\,\text{fm}$ configuration, as a function of the upper limit of the time integration t_c. The green squares show the results obtained through a chiral extrapolation from measurements performed at valence quark masses which are $\kappa = 3, 5, 7$ times larger than the physical light quark mass m_l. The red circles correspond to the measurements performed directly at $\kappa = 1$, and the blue crosses show the difference of the two methods.

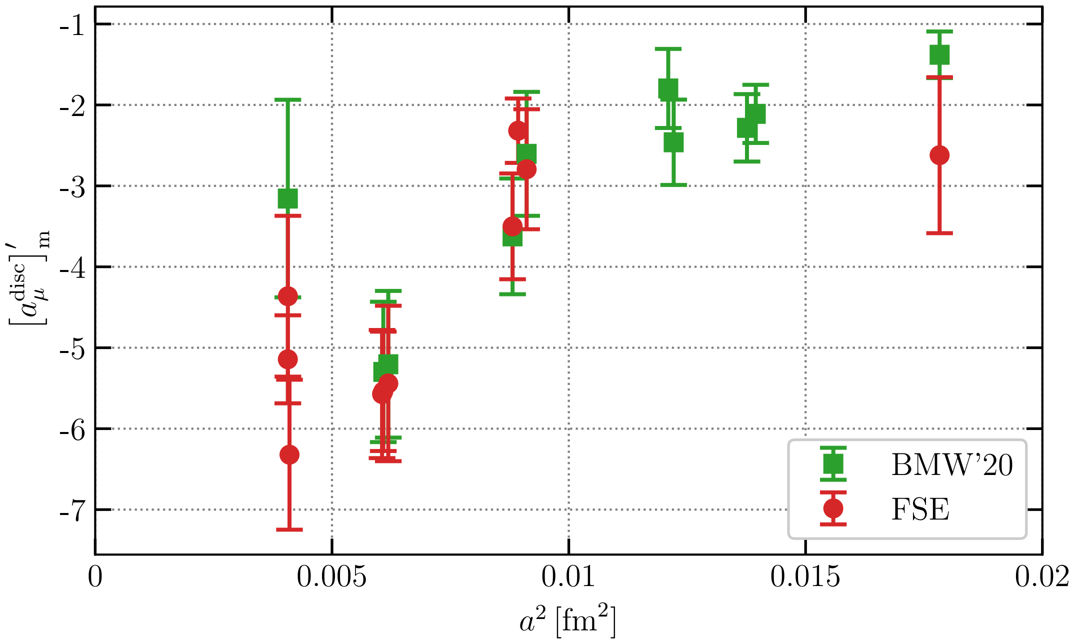

Figure S19: Strong-isospin-breaking (SIB) contribution to $a_\mu^{\rm disc}$. The green squares show the data points obtained using the finite difference-method of Ref. [1], with 3000 random source vectors. The red circles are obtained using the FSE technique of Giusti et. al. [152], with 128 random-source vectors.

appropriate choice, 2.2 fm and 2.7 fm, decreases the contribution by one standard deviation and the values in Figure 1 of the main text and in Table 15 of the Supplemental Information in [1] have to be changed as follows:

$$\text{light qed:} \quad -0.93(35)(47) \to -1.57(42)(35) \tag{S85}$$
$$\text{light qed-ss:} \quad 0.37(21)(24) \to -0.36(27)(15) \tag{S86}$$

Since in the present work we recompute the IB corrections to the $00-28$ window without further time-cuts, the numbers in Table S16 are unaffected.

In addition, we mistakenly subtracted a term related to one-photon-reducible contributions, $a_\mu^{1\gamma R}$, that we computed by means of infinite-volume QED. For consistency with our lattice setup, this term should also be computed in QED$_L$. Due to the zero spatial-momentum projection of the correlation function the spatial momentum of the photon vanishes. However, in the QED$_L$ prescription these particular modes are removed from the theory such that this subtraction is identically zero. Therefore in Table 16 of [1] the following change

$$\text{one-photon-reducible subtraction:} \quad -0.321(11) \to 0 \tag{S87}$$

has to be applied.

Regarding the total a_μ, we keep using the originally published number as our 2020 result, when we refer to it in this paper. As we mentioned, fixing the two mistakes would change a_μ only by a small fraction of its total error. In the new numbers of this work, these mistakes are of course corrected.

S10 Long-distance contributions

As emphasized in the main text, our goal is to compute the leading-order hadronic vacuum polarization contribution to the anomalous magnetic moment of the muon, with fully controlled uncertainties that are significantly smaller than available today.

The vast majority of our final result (over 95%) comes directly from lattice simulations. However, there is a contribution for which a data-driven approach yields an order of magnitude smaller uncertainty than a lattice calculation. It is the one corresponding to the large Euclidean-time "tail" of the current correlator. We choose it to be the contribution from a window [14] that extends from $2.8\,\text{fm}$ to ∞, for reasons explained below. We denote it $a_{\mu,28-\infty}$. Another set of contributions for which a data-driven approach can lead to uncertainty reduction is the one corresponding to finite-volume corrections, as discussed in Section S8. However, in the present work dedicated lattice simulations are used to compute those corrections, and the corresponding data-driven determinations are only used as cross-checks.

Before going into the details of how this tail contribution is obtained, it is important to clarify a number of issues. As is well known, the 2020 "Muon $g-2$ Theory Initiative" White Paper (WP '20) data-driven determination of the full LO-HVP contribution [31] leads to significant tensions between the standard-model prediction and the experimentally-measured value of $(g_\mu - 2)$ [4, 153]. In addition, our previous lattice determination of a_μ is 2.1σ larger than the one given in WP 20, a tension that rises to over 4σ when one considers the intermediate-distance window contribution $a_{\mu,04-10}$ [3, 32–36, 38, 45, 49]. To make the situation even more confusing, the recent measurement of the $e^+e^- \to \pi^+\pi^-$ spectrum by the CMD-3 collaboration [2, 154] leads to predictions for a_μ and for $a_{\mu,04-10}$ that are in good agreement with our 2020 lattice ones [23].

In that last reference [23], some of us reappraised the data-driven determination of a_μ, showing that results, based on measurements of the $\pi^+\pi^-$ spectrum by BaBar [15, 16], CMD-3 [2], KLOE[17–20] and via hadronic τ decays [21, 22], display significant tensions. This reappraisal was prompted by a detailed study of radiative corrections in initial-state radiation measurements.

In light of those challenges, one may wonder whether it is justified to consider a data-driven determination of $a_{\mu,28-\infty}$. To understand why it is, we emphasize a few important facts:

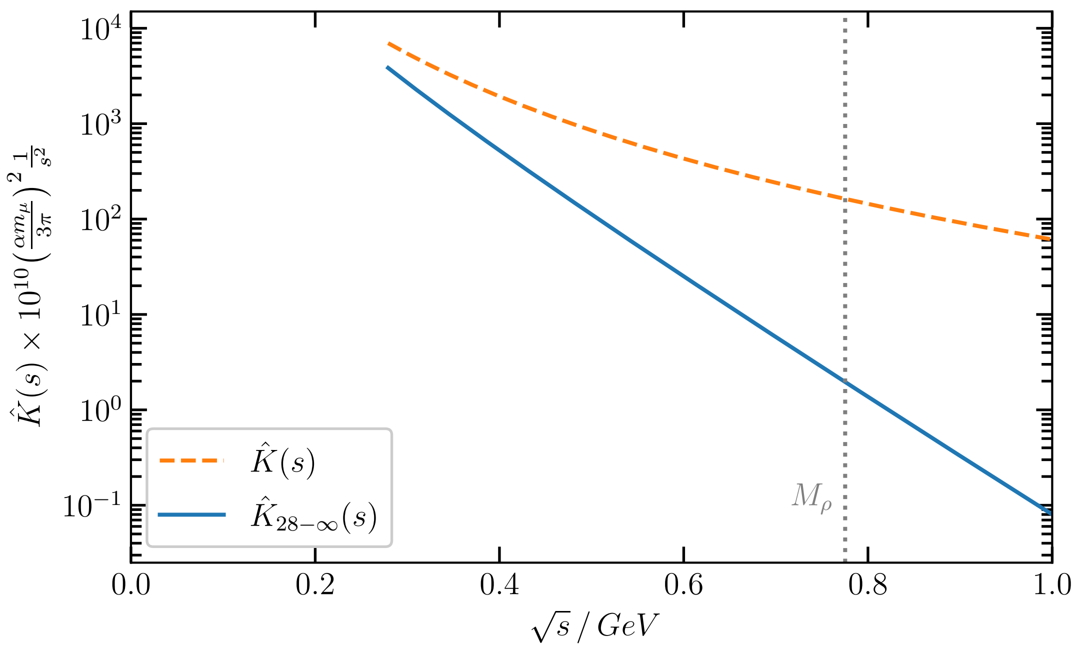

Figure S20: Kernels $\hat{K}(s)$ and $\hat{K}_{28-\infty}(s)$ plotted as a function of centre-of-mass energy $\sqrt{s}$ from the two-pion threshold to $1\,\mathrm{GeV}$. These kernels multiply the R-ratio $R(s)$ in the integrals over s which yield a_μ and its tail.

- The kernel, $\hat{K}_{28-\infty}(s)$, which multiplies the R-ratio in the integral that yields $a_{\mu,28-\infty}$ in the data-driven approach falls off much more quickly with increasing energy than the equivalent kernel for a_μ, $\hat{K}(s)$. This difference is illustrated in Figure S20. Thus, $a_{\mu,28-\infty}$ is far less sensitive than the full a_μ to the ρ peak, where the observed discrepancies between different experiments occur [3, 23].

- To visualize the effect of this exponential suppression on the data-driven determination of the tail we plot in Figure S21 the difference, with respect to a common reference, of the data for the two-pion contribution to $da_{\mu,28-\infty}/ds$, coming from all major experiments. The reference curve is used to give a common baseline to all the measurements. All experiments which have data for $\sqrt{s} \leq 0.55\,\mathrm{GeV}$ agree well within their uncertainties.

- As shown in Figure S22, using the $\pi^+\pi^-$ spectrum from each experiment that fully covers the energy range that is critical for this calculation, all four determinations of this tail contribution are entirely consistent. The χ^2/dof associated with the weighted average of those four determinations is 1.0. The reason is that the tail contribution is dominated by the low-mass part of the spectrum, below the ρ peak, where all four measurements are in good agreement, as discussed in the previous points.

- We have checked that the data-driven contribution to a_μ from $t \geq 2.8\,\mathrm{fm}$ is entirely compatible with our lattice calculation. This is shown in Figure S23 where we compare results for the window $28-35$ that accounts for close to 70% of the full tail, obtained from the four measurements and in lattice QCD. Here again the data-driven determinations are entirely compatible among themselves, with a χ^2/dof of 1.1, and also with our lattice result. The lattice calculation shown here validates the data-driven approach to the 8% level. This somewhat disadvantageous 8% uncertainty is the reason why we used the data-driven method for the tail observable.

- The total uncertainty on our average of the data-driven $a_{\mu,28-\infty}$, including the additional conservative uncertainties we add below, is 0.52 in our 10^{-10} units, a number that must be compared to our total uncertainty of 3.4 on a_μ. Even if the uncertainty on the tail were arbitrarily doubled, the effect on the total uncertainty would be insignificant.

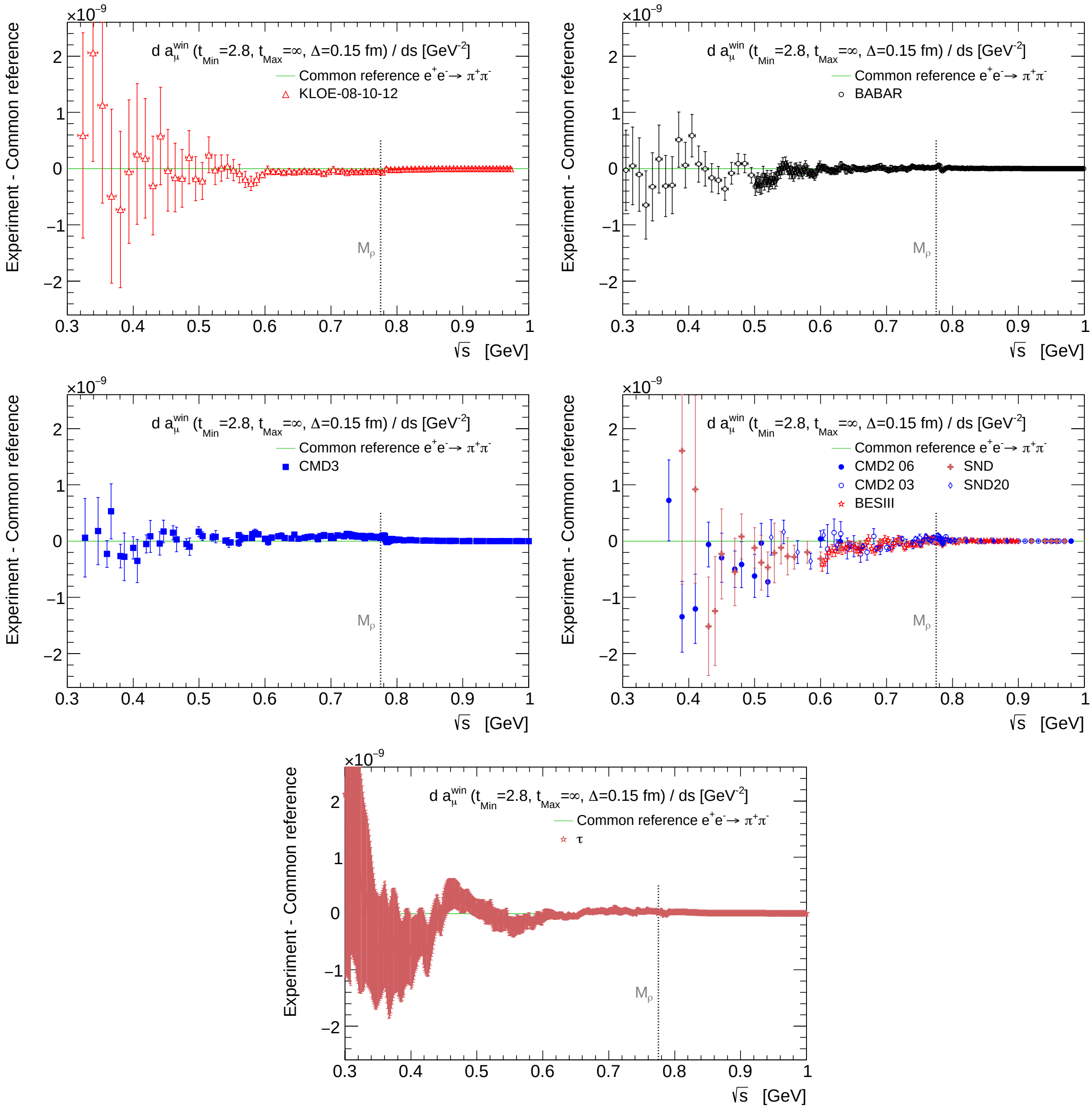

Figure S21: Difference with a common baseline of integrands for the tail contribution to a_μ from threshold to $1\,$GeV. The data points are obtained by multiplying the normalized two-pion cross-section measurements from different experiments with the tail kernel, $\hat{K}_{28-\infty}(s)$, and by subtracting from them the common baseline plotted as the green line at 0. Results are shown for KLOE (top left), BaBar (top right), CMD-3 (middle left), a set of measurements from BESIII, CMD-2 and SND (middle right), and from τ decays (bottom). All experiments which have data for $\sqrt{s} \leq 0.55\,$GeV agree well within their uncertainties. The higher-mass contributions are highly suppressed by the kernel for the tail. The difference in the areas caught between two datasets and the common reference (with an appropriate rescaling on the x-axis from $\sqrt{s}$ to s) measures the difference in their corresponding predictions for the two-pion contribution to $a_{\mu,28-\infty}$. Those differences are visibly small compared to the full size of that contribution, whose value is more than 26×10^{-10} but also to the large uncertainties present at low mass. This is made more quantitative in Figure S22 where we plot the individual results for $a_{\mu,28-\infty}$, obtained using the data of each of the four measurements of the $e^+e^- \to \pi^+\pi^-$ cross sections by BaBar [15, 16], CMD-3 [2], KLOE[17–20] and of the rate for $\tau^- \to \pi^-\pi^0\nu_\tau$ decays [21, 22].

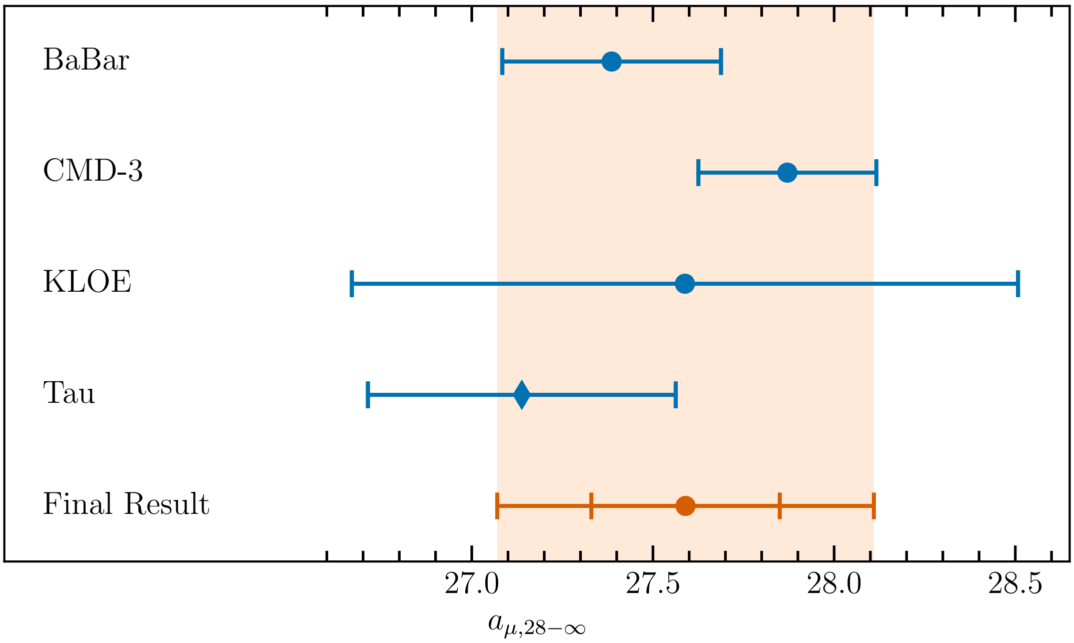

Figure S22: Results for $a_{\mu,28-\infty}$ obtained using the $\pi^+\pi^-$ spectra measured by BaBar, KLOE, CMD-3 and in τ decays. The orange circle at the bottom shows the weighted average. The outer error bars, and the corresponding shaded band include the additional uncertainty estimates that we conservatively include, obtained as described in the text.

Now, the reasons for choosing the tail to start at $t_{\text{cut}} = 2.8$ fm are the following:

- To ensure the result for a_μ presented in this paper is dominated by the lattice contribution, we choose to start the data-driven tail above $t = 2.8\,\text{fm}$. This guarantees that the lattice contribution accounts for over 95% of the result.
- Beyond reducing the uncertainty on $a_{\mu,28-\infty}$ by an order of magnitude, the use of a data-driven tail reduces the finite-volume correction that must be applied to the lattice result by a factor of 2 and the associated uncertainty by even more.
- As discussed above, for these large times the data-driven determinations of $a_{\mu,28-\infty}$ agree very well.

Having justified the use of a data-driven approach for determining the tail contribution, we now explain briefly how it is calculated. As mentioned above, the computation is performed following the approach of Ref. [23]. In that work, the measurements of the $\pi^+\pi^-$ spectrum by BaBar [15, 16], KLOE [17–20], CMD-3 [2] and via hadronic τ decays [21, 22] are considered separately. Outside their centre-of-mass energy ranges and for other hadronic channels, the data from each experiment are complemented by the combined experimental and perturbative QCD results compiled in Ref. [13, 22], with a full treatment of uncertainties and correlations. For BaBar and τ decays, centre-of-mass energies range from $0.3\,\text{GeV}$ and $2M_{\pi^\pm}$ to $1.8\,\text{GeV}$, and for CMD-3, $0.33\,\text{GeV}$ up to $1.2\,\text{GeV}$. KLOE covers the range from $0.32\,\text{GeV}$ to $0.97\,\text{GeV}$.

The HVPTools framework [10–13] is then used to Laplace transform [6] these four spectra into the corresponding Euclidean-time correlators. The latter are subsequently integrated, with the window weights of Ref. [14], to give the four data-driven results for $a_{\mu,28-\infty}$. As discussed above, the tail contributions are shown in Figure S22 and the comparison of results for the $28-35$ window in Figure S23.

To obtain our final results for the tail and the $28-35$ window contributions, we perform weighted averages of the BaBar, KLOE and CMD-3 and τ-decay determinations, taking into account all correlations. This procedure yields our final central values for those quantities, as well as the uncertainties originating from those in the cross sections.

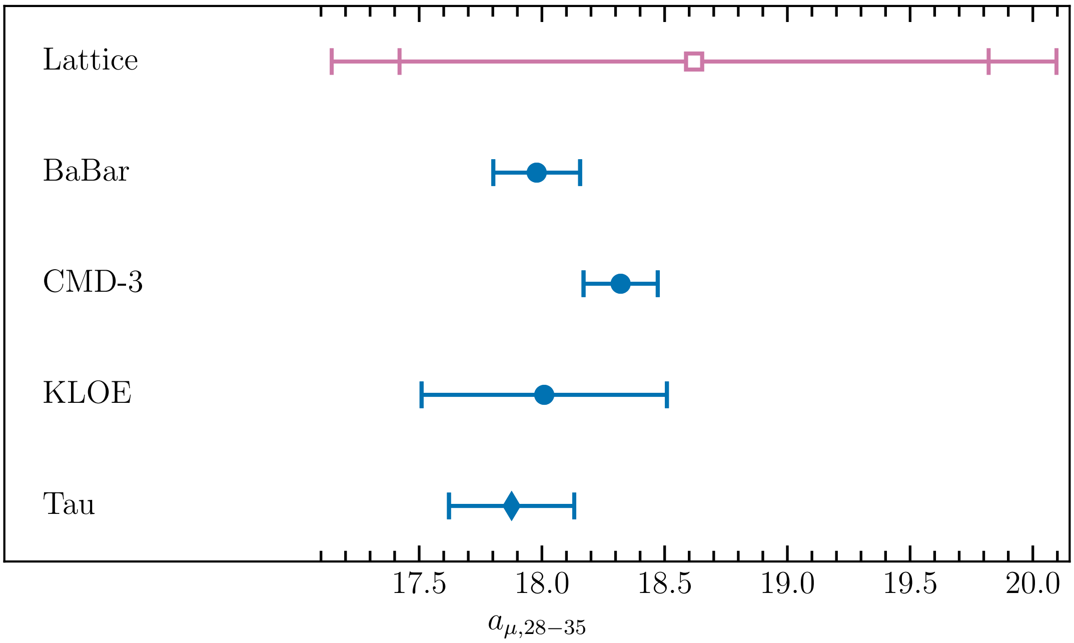

Figure S23: Results for $a_{\mu,28-35}$. Figure description is the same as in Figure S22. Additionally, the pink square on the top represents our lattice result, which is in excellent agreement with the data-driven determinations.

The agreement of the results using the different data sets is excellent. The χ^2/dof for $a_{\mu,28-\infty}$ is less than 1.

The agreement between the results obtained using different data sets fully extends to those determined via τ data. The use of τ data in this context requires that one estimates isospin-breaking corrections. This was done very carefully in Ref. [21]. Nevertheless, we conservatively consider the absolute value of the full difference between the averages, obtained with and without the τ-decay data, as an additional uncertainty. We add it linearly to the other uncertainties. In that way, our error bar necessarily covers the average in which only e^+e^- data are used. For both quantities studied in the present section this additional uncertainty is smaller than the one induced by those in the cross sections.

Altogether, we obtain

$$a^{\mathrm{avg}}_{\mu,28-\infty} = 27.59(17)(9)[26] \qquad \text{(S88)}$$

as the starting point of our tail-related window result in the data-driven approach. The first error comes from propagating the uncertainties of the results used in the weighted average. Here no PDG-style error rescaling is needed since the two χ^2/dof are less than 1. The second error is the additional uncertainty from including or not including the τ data set. The third, conservative total error is the first two added linearly.

We consider several sources of systematic uncertainties on the value in Equation (S88). For this purpose we use the well-tested HVPTools framework, which combines spectra of different experiments in a local fashion and integrates the combination. For this error estimation we focus on the two-pion contribution[14] in the interval of $\sqrt{s} \in [0.3, 1.8]$ GeV, which provides over 97% of $a_{\mu,28-\infty}$. Also we take only the $e^+e^- \to \pi^+\pi^-$ measurements, but not the τ data set. Using these analyses we derive the following systematics:

1. In our value in Equation (S88) we use a weighted average of integrals of the two-pion spectra obtained by individual experiments. Instead, we could perform the weighted average of the spectra first, followed by the integration. The difference between the two approaches is 0.12 in units of 10^{-10}.

[14] A study of the two-pion contribution to $a_{\mu,28-\infty}$ using a dispersive approach was recently performed in [155].

2. In our value in Equation (S88) we use four data-sets, which have almost full coverage in the relevant energy region. This allows for a simple analysis procedure, integrating the spectra first and then averaging. These data-sets turn out to agree on the tail observable. Agreement is no guarantee of reliability, so we investigate adding other experiments with less energy coverage, the principal ones being BESIII [156], CMD-2 [157–160], SND06 [161], SND20 [162] and CLEO [163]. They can only be included in an approach combining the actual spectra, this was performed in [23], which we will refer to as DHLMZ23. The difference between the combinations with and without those experiments is 0.16.

3. In our determination of the tail the recent CMD-3 measurement provides a very important contribution, because of its precision in the low-mass region. We test the sensitivity of our result to this single measurement by removing it from the average. For this purpose we turn to the combination performed in [13], which we will refer to as DHMZ19. The difference of the DHLMZ23 and DHMZ19 combinations, 0.31, quantifies the effect of removing the CMD-3 dataset. (Note, that the two combinations also differ in the SND20 experiment, but it only plays a small role here.)

4. Even without the CMD-3 experiment there are well-known tensions on the total a_μ (dominated by the ρ-meson peak) in the data-driven approach between the BaBar and KLOE datasets. As we discussed earlier, this is far less important in the tail contribution. We quantify its impact by looking at the difference of the DHMZ19 combination obtained by alternatively removing each of those two experiments. This difference is 0.26. As discussed in WP '20, the BaBar/KLOE tension has a direct impact on the difference between the DHMZ19 and an alternative analysis approach KNT19 [41]. Actually this latter difference is also 0.26[15].

We take all these variations and add them up in quadrature to get our uncertainty estimate

$$(0.12)_{\rm int-avg}(0.16)_{\rm other\ exp.}(0.31)_{\rm CMD3}(0.26)_{\rm BaBar/KLOE} \to (0.45) \tag{S89}$$

on the two-pion contribution to the tail observable. Combined with the number in Equation (S88), this gives our final result for the tail:

$$a_{\mu,28-\infty} = 27.59(26)(45)[52] \,, \tag{S90}$$

with a total uncertainty of 1.9%. This is the result that we add to our lattice determination of the complementary window, $a_{\mu,00-28}$, to obtain our final result for the HVP contribution to the muon. It is important to note that even if the uncertainty on the tail was multiplied by a factor two, the final uncertainty on our result for a_μ would change insignificantly.

References

1. Borsányi, S. *et al.* Leading hadronic contribution to the muon magnetic moment from lattice QCD. *Nature* **593,** 51–55. arXiv: 2002.12347 [hep-lat] (2021).
2. Ignatov, F. V. *et al.* Measurement of the $e^+e^- \to \pi^+\pi^-$ cross section from threshold to 1.2 GeV with the CMD-3 detector. *Phys. Rev. D* **109,** 112002. arXiv: 2302.08834 [hep-ex] (2024).
3. Aliberti, R. *et al.* The anomalous magnetic moment of the muon in the Standard Model: an update. *Phys. Rept.* **1143,** 1–158. arXiv: 2505.21476 [hep-ph] (2025).
4. Aguillard, D. P. *et al.* Measurement of the Positive Muon Anomalous Magnetic Moment to 127 ppb. *Phys. Rev. Lett.* **135,** 101802. arXiv: 2506.03069 [hep-ex] (2025).
6. Bernecker, D. & Meyer, H. B. Vector Correlators in Lattice QCD: Methods and applications. *Eur. Phys. J.* **A47,** 148. arXiv: 1107.4388 [hep-lat] (2011).

[15] We are very grateful to A. Keshavarzi, D. Nomura and T. Teubner for sharing their KNT19 compilation, which we use to compute this result.

10. Davier, M., Höcker, A., Malaescu, B., Yuan, C. Z. & Zhang, Z. Reevaluation of the hadronic contribution to the muon magnetic anomaly using new $e^+e^- \to \pi^+\pi^-$ cross section data from BABAR. *Eur. Phys. J. C* **66,** 1–9. arXiv: 0908.4300 [hep-ph] (2010).
11. Davier, M., Höcker, A., Malaescu, B. & Zhang, Z. Reevaluation of the Hadronic Contributions to the Muon $g-2$ and to $\alpha(M_Z)$. *Eur. Phys. J. C* **71.** [Erratum: Eur.Phys.J.C 72, 1874 (2012)], 1515. arXiv: 1010.4180 [hep-ph] (2011).
12. Davier, M., Höcker, A., Malaescu, B. & Zhang, Z. Reevaluation of the hadronic vacuum polarisation contributions to the Standard Model predictions of the muon $g-2$ and $\alpha(M_Z^2)$ using newest hadronic cross-section data. *Eur. Phys. J.* **C77,** 827. arXiv: 1706.09436 [hep-ph] (2017).
13. Davier, M., Höcker, A., Malaescu, B. & Zhang, Z. A new evaluation of the hadronic vacuum polarisation contributions to the muon anomalous magnetic moment and to $\alpha(M_Z^2)$. *Eur. Phys. J. C* **80,** 241. arXiv: 1908.00921 [hep-ph] (2020).
14. Blum, T. *et al.* Calculation of the hadronic vacuum polarization contribution to the muon anomalous magnetic moment. *Phys. Rev. Lett.* **121,** 022003. arXiv: 1801.07224 [hep-lat] (2018).
15. Aubert, B. *et al.* Precise measurement of the $e^+e^- \to \pi^+\pi^-(\gamma)$ cross section with the Initial State Radiation method at BABAR. *Phys. Rev. Lett.* **103,** 231801. arXiv: 0908.3589 [hep-ex] (2009).
16. Lees, J. P. *et al.* Precise Measurement of the $e^+e^- \to \pi^+\pi^-(\gamma)$ Cross Section with the Initial-State Radiation Method at BABAR. *Phys. Rev. D* **86,** 032013. arXiv: 1205.2228 [hep-ex] (2012).
17. Ambrosino, F. *et al.* Measurement of $\sigma(e^+e^- \to \pi^+\pi^-\gamma(\gamma))$ and the dipion contribution to the muon anomaly with the KLOE detector. *Phys. Lett. B* **670,** 285–291. arXiv: 0809.3950 [hep-ex] (2009).
18. Ambrosino, F. *et al.* Measurement of $\sigma(e^+e^- \to \pi^+\pi^-)$ from threshold to 0.85 GeV^2 using Initial State Radiation with the KLOE detector. *Phys. Lett. B* **700,** 102–110. arXiv: 1006.5313 [hep-ex] (2011).
19. Babusci, D. *et al.* Precision measurement of $\sigma(e^+e^- \to \pi^+\pi^-\gamma)/\sigma(e^+e^- \to \mu^+\mu^-\gamma)$ and determination of the $\pi^+\pi^-$ contribution to the muon anomaly with the KLOE detector. *Phys. Lett. B* **720,** 336–343. arXiv: 1212.4524 [hep-ex] (2013).
20. Anastasi, A. *et al.* Combination of KLOE $\sigma\big(e^+e^- \to \pi^+\pi^-\gamma(\gamma)\big)$ measurements and determination of $a_\mu^{\pi^+\pi^-}$ in the energy range $0.10 < s < 0.95$ GeV^2. *JHEP* **03,** 173. arXiv: 1711.03085 [hep-ex] (2018).
21. Davier, M. *et al.* The Discrepancy Between τ and e^+e^- Spectral Functions Revisited and the Consequences for the Muon Magnetic Anomaly. *Eur. Phys. J. C* **66,** 127–136. arXiv: 0906.5443 [hep-ph] (2010).
22. Davier, M., Höcker, A., Malaescu, B., Yuan, C.-Z. & Zhang, Z. Update of the ALEPH non-strange spectral functions from hadronic τ decays. *Eur. Phys. J. C* **74,** 2803. arXiv: 1312.1501 [hep-ex] (2014).
23. Davier, M., Höcker, A., Lutz, A.-M., Malaescu, B. & Zhang, Z. Tensions in $e^+e^- \to \pi^+\pi^-(\gamma)$ measurements: the new landscape of data-driven hadronic vacuum polarization predictions for the muon $g-2$. *Eur. Phys. J. C* **84,** 721. arXiv: 2312.02053 [hep-ph] (2024).
24. Borsányi, S. *et al.* Hadronic vacuum polarization contribution to the anomalous magnetic moments of leptons from first principles. *Phys. Rev. Lett.* **121,** 022002. arXiv: 1711.04980 [hep-lat] (2018).
25. Aubin, C. *et al.* Light quark vacuum polarization at the physical point and contribution to the muon $g-2$. *Phys. Rev. D* **101,** 014503. arXiv: 1905.09307 [hep-lat] (2020).
26. Meyer, H. B. Lattice QCD and the Timelike Pion Form Factor. *Phys. Rev. Lett.* **107,** 072002. arXiv: 1105.1892 [hep-lat] (2011).

27. Lellouch, L. & Lüscher, M. Weak transition matrix elements from finite volume correlation functions. *Commun. Math. Phys.* **219,** 31–44. arXiv: hep-lat/0003023 [hep-lat] (2001).

28. Lüscher, M. Two particle states on a torus and their relation to the scattering matrix. *Nucl. Phys.* **B354,** 531–578 (1991).

29. Hansen, M. T. & Patella, A. Finite-volume effects in $(g-2)_\mu^{\text{HVP,LO}}$. *Phys. Rev. Lett.* **123,** 172001. arXiv: 1904.10010 [hep-lat] (2019).

30. Hansen, M. T. & Patella, A. Finite-volume and thermal effects in the leading-HVP contribution to muonic $(g-2)$. *JHEP* **10,** 029. arXiv: 2004.03935 [hep-lat] (2020).

31. Aoyama, T. *et al.* The anomalous magnetic moment of the muon in the Standard Model. *Phys. Rept.* **887,** 1–166. arXiv: 2006.04822 [hep-ph] (2020).

32. Lehner, C. & Meyer, A. S. Consistency of hadronic vacuum polarization between lattice QCD and the R-ratio. *Phys. Rev. D* **101,** 074515. arXiv: 2003.04177 [hep-lat] (2020).

33. Wang, G., Draper, T., Liu, K.-F. & Yang, Y.-B. Muon $g-2$ with overlap valence fermions. *Phys. Rev. D* **107,** 034513. arXiv: 2204.01280 [hep-lat] (2023).

34. Aubin, C., Blum, T., Golterman, M. & Peris, S. Muon anomalous magnetic moment with staggered fermions: Is the lattice spacing small enough? *Phys. Rev. D* **106,** 054503. arXiv: 2204.12256 [hep-lat] (2022).

35. Cè, M. *et al.* Window observable for the hadronic vacuum polarization contribution to the muon $g-2$ from lattice QCD. *Phys. Rev. D* **106,** 114502. arXiv: 2206.06582 [hep-lat] (2022).

36. Alexandrou, C. *et al.* Lattice calculation of the short and intermediate time-distance hadronic vacuum polarization contributions to the muon magnetic moment using twisted-mass fermions. *Phys. Rev. D* **107,** 074506. arXiv: 2206.15084 [hep-lat] (2023).

37. Bazavov, A. *et al.* Light-quark connected intermediate-window contributions to the muon $g-2$ hadronic vacuum polarization from lattice QCD. *Phys. Rev. D* **107,** 114514. arXiv: 2301.08274 [hep-lat] (2023).

38. Blum, T. *et al.* Update of Euclidean windows of the hadronic vacuum polarization. *Phys. Rev. D* **108,** 054507. arXiv: 2301.08696 [hep-lat] (2023).

39. Benton, G. *et al.* Data-Driven Determination of the Light-Quark Connected Component of the Intermediate-Window Contribution to the Muon $g-2$. *Phys. Rev. Lett.* **131,** 251803. arXiv: 2306.16808 [hep-ph] (2023).

41. Keshavarzi, A., Nomura, D. & Teubner, T. $g-2$ of charged leptons, $\alpha(M_Z^2)$, and the hyperfine splitting of muonium. *Phys. Rev.* **D101,** 014029. arXiv: 1911.00367 [hep-ph] (2020).

44. Kuberski, S. *et al.* Hadronic vacuum polarization in the muon $g-2$: the short-distance contribution from lattice QCD. *JHEP* **03,** 172. arXiv: 2401.11895 [hep-lat] (2024).

45. Bazavov, A. *et al.* Hadronic vacuum polarization for the muon $g-2$ from lattice QCD: Complete short and intermediate windows. *Phys. Rev. D* **111,** 094508. arXiv: 2411.09656 [hep-lat] (2025).

46. Spiegel, S. & Lehner, C. High-precision continuum limit study of the HVP short-distance window. *Phys. Rev. D* **111,** 114517. arXiv: 2410.17053 [hep-lat] (2025).

49. Davier, M. *et al.* Hadronic vacuum polarization: Comparing lattice QCD and data-driven results in systematically improvable ways. *Phys. Rev. D* **109,** 076019. arXiv: 2308.04221 [hep-ph] (2024).

51. Blum, T. *et al.* Long-Distance Window of the Hadronic Vacuum Polarization for the Muon $g-2$. *Phys. Rev. Lett.* **134,** 201901. arXiv: 2410.20590 [hep-lat] (2025).

52. Djukanovic, D. *et al.* The hadronic vacuum polarization contribution to the muon $g-2$ at long distances. *JHEP* **04,** 098. arXiv: 2411.07969 [hep-lat] (2025).

53. Lüscher, M. & Weisz, P. On-Shell Improved Lattice Gauge Theories. *Commun. Math. Phys.* **97.** [Erratum: Commun. Math. Phys.98,433(1985)], 59 (1985).

54. Morningstar, C. & Peardon, M. J. Analytic smearing of SU(3) link variables in lattice QCD. *Phys. Rev.* **D69,** 054501. arXiv: hep-lat/0311018 [hep-lat] (2004).

55. Clark, M. A., Babich, R., Barros, K., Brower, R. C. & Rebbi, C. Solving Lattice QCD systems of equations using mixed precision solvers on GPUs. *Comput. Phys. Commun.* **181,** 1517–1528. arXiv: 0911.3191 [hep-lat] (2010).

56. Babich, R. *et al. Scaling lattice QCD beyond 100 GPUs* in *International Conference for High Performance Computing, Networking, Storage and Analysis* (Sept. 2011). arXiv: 1109.2935 [hep-lat].

57. https://github.com/jinluchang/Qlattice.

58. Hunter, J. D. Matplotlib: A 2D graphics environment. *Computing in Science & Engineering* **9,** 90–95 (2007).

59. McNeile, C., Davies, C. T. H., Follana, E., Hornbostel, K. & Lepage, G. P. High-precision c and b masses, and QCD coupling from current-current correlators in lattice and continuum QCD. *Phys. Rev. D* **82,** 034512. arXiv: 1004.4285 [hep-lat] (2010).

60. Aoki, Y. *et al.* FLAG Review 2021. *Eur. Phys. J. C* **82,** 869. arXiv: 2111.09849 [hep-lat] (2022).

61. Carrasco, N. *et al.* Up, down, strange and charm quark masses with $N_f = 2+1+1$ twisted mass lattice QCD. *Nucl. Phys. B* **887,** 19–68. arXiv: 1403.4504 [hep-lat] (2014).

62. Chakraborty, B. *et al.* High-precision quark masses and QCD coupling from $n_f = 4$ lattice QCD. *Phys. Rev. D* **91,** 054508. arXiv: 1408.4169 [hep-lat] (2015).

63. Bazavov, A. *et al.* Up-, down-, strange-, charm-, and bottom-quark masses from four-flavor lattice QCD. *Phys. Rev. D* **98,** 054517. arXiv: 1802.04248 [hep-lat] (2018).

64. Alexandrou, C. *et al.* Quark masses using twisted-mass fermion gauge ensembles. *Phys. Rev. D* **104,** 074515. arXiv: 2104.13408 [hep-lat] (2021).

65. Clark, M. A. & Kennedy, A. D. Accelerating dynamical fermion computations using the rational hybrid Monte Carlo (RHMC) algorithm with multiple pseudofermion fields. *Phys. Rev. Lett.* **98,** 051601. arXiv: hep-lat/0608015 (2007).

66. Yin, H. & Mawhinney, R. D. Improving DWF Simulations: the Force Gradient Integrator and the Möbius Accelerated DWF Solver. *PoS* **LATTICE2011** (ed Vranas, P.) 051. arXiv: 1111.5059 [hep-lat] (2011).

67. Sexton, J. C. & Weingarten, D. H. Hamiltonian evolution for the hybrid Monte Carlo algorithm. *Nucl. Phys. B* **380,** 665–677 (1992).

68. Hasenbusch, M. Speeding up the hybrid Monte Carlo algorithm for dynamical fermions. *Phys. Lett. B* **519,** 177–182. arXiv: hep-lat/0107019 (2001).

69. Schaefer, S., Sommer, R. & Virotta, F. Critical slowing down and error analysis in lattice QCD simulations. *Nucl. Phys. B* **845,** 93–119. arXiv: 1009.5228 [hep-lat] (2011).

70. Joswig, F., Kuberski, S., Kuhlmann, J. T. & Neuendorf, J. pyerrors: A python framework for error analysis of Monte Carlo data. *Comput. Phys. Commun.* **288,** 108750. arXiv: 2209.14371 [hep-lat] (2023).

71. Borsányi, S. *et al.* Calculation of the axion mass based on high-temperature lattice quantum chromodynamics. *Nature* **539,** 69–71. arXiv: 1606.07494 [hep-lat] (2016).

72. Ishizuka, N., Fukugita, M., Mino, H., Okawa, M. & Ukawa, A. Operator dependence of hadron masses for Kogut-Susskind quarks on the lattice. *Nucl. Phys.* **B411,** 875–902 (1994).

73. Bazavov, A. *et al.* $|V_{us}|$ from $K_{\ell 3}$ decay and four-flavor lattice QCD. *Phys. Rev. D* **99,** 114509. arXiv: 1809.02827 [hep-lat] (2019).

74. Golterman, M. F. L. & Smit, J. Lattice Baryons With Staggered Fermions. *Nucl. Phys.* **B255,** 328–340 (1985).
75. Bailey, J. A. Staggered baryon operators with flavor SU(3) quantum numbers. *Phys. Rev.* **D75,** 114505. arXiv: hep-lat/0611023 [hep-lat] (2007).
76. Gusken, S. *et al.* Nonsinglet Axial Vector Couplings of the Baryon Octet in Lattice QCD. *Phys. Lett.* **B227,** 266–269 (1989).
77. Billoire, A., Marinari, E. & Parisi, G. Computing the hadronic mass spectrum. Eight is better than one. *Phys. Lett. B* **162,** 160–164 (1985).
78. Blossier, B., Della Morte, M., von Hippel, G., Mendes, T. & Sommer, R. On the generalized eigenvalue method for energies and matrix elements in lattice field theory. *JHEP* **04,** 094. arXiv: 0902.1265 [hep-lat] (2009).
79. Aubin, C. & Orginos, K. A new approach for Delta form factors. *AIP Conf. Proc.* **1374,** 621–624. arXiv: 1010.0202 [hep-lat] (2011).
80. Yelton, J. *et al.* Observation of an Excited Ω^- Baryon. *Phys. Rev. Lett.* **121,** 052003. arXiv: 1805.09384 [hep-ex] (2018).
81. Bouchard, C. M., Lepage, G. P., Monahan, C., Na, H. & Shigemitsu, J. $B_s \to K\ell\nu$ form factors from lattice QCD. *Phys. Rev. D* **90,** 054506. arXiv: 1406.2279 [hep-lat] (2014).
82. Bruno, M. & Sommer, R. On fits to correlated and auto-correlated data. *Comput. Phys. Commun.* **285,** 108643. arXiv: 2209.14188 [hep-lat] (2023).
83. Bär, O. Nucleon-pion-state contribution to nucleon two-point correlation functions. *Phys. Rev. D* **92,** 074504. arXiv: 1503.03649 [hep-lat] (2015).
84. Tiburzi, B. C. Chiral Corrections to Nucleon Two- and Three-Point Correlation Functions. *Phys. Rev. D* **91,** 094510. arXiv: 1503.06329 [hep-lat] (2015).
85. Navas, S. *et al.* Review of particle physics. *Phys. Rev. D* **110,** 030001 (2024).
86. Michael, C. Fitting correlated data. *Phys. Rev. D* **49,** 2616–2619. arXiv: hep-lat/9310026 (1994).
87. Bijnens, J. & Rössler, T. Finite Volume at Two-loops in Chiral Perturbation Theory. *JHEP* **01,** 034. arXiv: 1411.6384 [hep-lat] (2015).
88. Bijnens, J. CHIRON: a package for ChPT numerical results at two loops. *Eur. Phys. J. C* **75,** 27. arXiv: 1412.0887 [hep-ph] (2015).
89. Aubin, C. & Bernard, C. Pion and kaon masses in staggered chiral perturbation theory. *Phys. Rev.* **D68,** 034014. arXiv: hep-lat/0304014 [hep-lat] (2003).
90. Aubin, C. & Bernard, C. Pseudoscalar decay constants in staggered chiral perturbation theory. *Phys. Rev. D* **68,** 074011. arXiv: hep-lat/0306026 (2003).
91. Bailey, J. A., Kim, H.-J. & Lee, W. Taste non-Goldstone, flavor-charged pseudo-Goldstone boson masses in staggered chiral perturbation theory. *Phys. Rev. D* **85,** 094503. arXiv: 1112.2108 [hep-lat] (2012).
92. Bailey, J. A., Lee, W. & Yoon, B. Taste non-Goldstone, flavor-charged pseudo-Goldstone boson decay constants in staggered chiral perturbation theory. *Phys. Rev. D* **87,** 054508. arXiv: 1212.5369 [hep-lat] (2013).
93. Desiderio, A. *et al.* First lattice calculation of radiative leptonic decay rates of pseudoscalar mesons. *Phys. Rev. D* **103,** 014502. arXiv: 2006.05358 [hep-lat] (2021).
94. Frezzotti, R. *et al.* Comparison of lattice QCD+QED predictions for radiative leptonic decays of light mesons with experimental data. *Phys. Rev. D* **103,** 053005. arXiv: 2012.02120 [hep-ph] (2021).

95. Di Palma, R. *et al.* Kaon radiative leptonic decay rates from lattice QCD simulations at the physical point. *Phys. Rev. D* **111,** 114523. arXiv: 2504.08680 [hep-lat] (2025).
96. Di Carlo, M. *et al.* Light-meson leptonic decay rates in lattice QCD+QED. *Phys. Rev.* **D100,** 034514. arXiv: 1904.08731 [hep-lat] (2019).
97. Knecht, M., Neufeld, H., Rupertsberger, H. & Talavera, P. Chiral perturbation theory with virtual photons and leptons. *Eur. Phys. J. C* **12,** 469–478. arXiv: hep-ph/9909284 (2000).
98. Bijnens, J. & Danielsson, N. Electromagnetic Corrections in Partially Quenched Chiral Perturbation Theory. *Phys. Rev.* **D75,** 014505. arXiv: hep-lat/0610127 [hep-lat] (2007).
99. Lubicz, V. *et al.* Finite-Volume QED Corrections to Decay Amplitudes in Lattice QCD. *Phys. Rev. D* **95,** 034504. arXiv: 1611.08497 [hep-lat] (2017).
100. Aoki, Y. *et al.* FLAG review 2024. *Phys. Rev. D* **113,** 014508. arXiv: 2411.04268 [hep-lat] (2026).
101. Carrasco, N. *et al.* $K \to \pi$ semileptonic form factors with $N_f = 2+1+1$ twisted mass fermions. *Phys. Rev.* **D93,** 114512. arXiv: 1602.04113 [hep-lat] (2016).
102. Alexandrou, C. *et al.* Ratio of kaon and pion leptonic decay constants with $N_f = 2+1+1$ Wilson-clover twisted-mass fermions. *Phys. Rev. D* **104,** 074520. arXiv: 2104.06747 [hep-lat] (2021).
103. Miller, N. *et al.* F_K/F_π from Möbius Domain-Wall fermions solved on gradient-flowed HISQ ensembles. *Phys. Rev. D* **102,** 034507. arXiv: 2005.04795 [hep-lat] (2020).
104. Bazavov, A. *et al.* B- and D-meson leptonic decay constants from four-flavor lattice QCD. *Phys. Rev.* **D98,** 074512. arXiv: 1712.09262 [hep-lat] (2018).
105. Carrasco, N. *et al.* Leptonic decay constants $f_K, f_D,$ and f_{D_s} with $N_f = 2+1+1$ twisted-mass lattice QCD. *Phys. Rev.* **D91,** 054507. arXiv: 1411.7908 [hep-lat] (2015).
106. Dowdall, R., Davies, C., Lepage, G. & McNeile, C. V_{us} from π and K decay constants in full lattice QCD with physical u, d, s and c quarks. *Phys. Rev. D* **88,** 074504. arXiv: 1303.1670 [hep-lat] (2013).
107. Gasser, J., Rusetsky, A. & Scimemi, I. Electromagnetic corrections in hadronic processes. *Eur. Phys. J.* **C32,** 97–114. arXiv: hep-ph/0305260 [hep-ph] (2003).
108. Giusti, D. *et al.* Leading isospin-breaking corrections to pion, kaon and charmed-meson masses with Twisted-Mass fermions. *Phys. Rev. D* **95,** 114504. arXiv: 1704.06561 [hep-lat] (2017).
109. Husung, N., Marquard, P. & Sommer, R. Asymptotic behavior of cutoff effects in Yang–Mills theory and in Wilson's lattice QCD. *Eur. Phys. J. C* **80,** 200. arXiv: 1912.08498 [hep-lat] (2020).
110. Lüscher, M. Properties and uses of the Wilson flow in lattice QCD. *JHEP* **08.** [Erratum: JHEP03,092(2014)], 071. arXiv: 1006.4518 [hep-lat] (2010).
111. Ramos, A. & Sint, S. Symanzik improvement of the gradient flow in lattice gauge theories. *Eur. Phys. J. C* **76,** 15. arXiv: 1508.05552 [hep-lat] (2016).
112. Borsányi, S. *et al.* Isospin splittings in the light baryon octet from lattice QCD and QED. *Phys. Rev. Lett.* **111,** 252001. arXiv: 1306.2287 [hep-lat] (2013).
113. Miller, N. *et al.* Scale setting the Möbius domain wall fermion on gradient-flowed HISQ action using the omega baryon mass and the gradient-flow scales t_0 and w_0. *Phys. Rev. D* **103,** 054511. arXiv: 2011.12166 [hep-lat] (2021).
114. Bazavov, A. *et al.* Gradient flow and scale setting on MILC HISQ ensembles. *Phys. Rev. D* **93,** 094510. arXiv: 1503.02769 [hep-lat] (2016).
115. Bergner, G. *et al.* Quark masses and decay constants in $N_f = 2+1+1$ isoQCD with Wilson clover twisted mass fermions. *PoS* **LATTICE2019,** 181. arXiv: 2001.09116 [hep-lat] (2020).

116. Stamen, D., Hariharan, D., Hoferichter, M., Kubis, B. & Stoffer, P. Kaon electromagnetic form factors in dispersion theory. *Eur. Phys. J. C* **82,** 432. arXiv: 2202.11106 [hep-ph] (2022).
117. Horsley, R. *et al.* QED effects in the pseudoscalar meson sector. *JHEP* **04,** 093. arXiv: 1509.00799 [hep-lat] (2016).
118. Cottingham, W. N. The neutron proton mass difference and electron scattering experiments. *Annals Phys.* **25,** 424–432 (1963).
119. Husung, N. Logarithmic corrections to O(a^2) effects in lattice QCD with unrooted Staggered quarks. arXiv: 2501.17036 [hep-lat] (Jan. 2025).
120. Akaike, H. *Information theory and an extension of the maximum likelihood principle* in *2nd International Symposium on Information Theory* (eds Petrov, B. & Csaki, F.) (Akademiai Kiado, Budapest, 1973), 267–281.
121. Akaike, H. A new look at the statistical model identification. *IEEE Trans. Automatic Control* **19,** 716–723 (1974).
122. Akaike, H. On the likelihood of a time series model. *The Statistician* **27,** 217–235 (1978).
123. Jay, W. I. & Neil, E. T. Bayesian model averaging for analysis of lattice field theory results. *Phys. Rev. D* **103,** 114502. arXiv: 2008.01069 [stat.ME] (2021).
124. Kullback, S. & Leibler, R. A. On Information and Sufficiency. *The Annals of Mathematical Statistics* **22,** 79–86 (1951).
125. Boyle, P. *et al.* Physical-mass calculation of $\rho(770)$ and $K^*(892)$ resonance parameters via $\pi\pi$ and $K\pi$ scattering amplitudes from lattice QCD. *Phys. Rev. D* **111,** 054510. arXiv: 2406.19193 [hep-lat] (2025).
126. https://doi.org/10.5281/zenodo.17880027.
127. Cè, M., Harris, T., Meyer, H. B., Toniato, A. & Török, C. Vacuum correlators at short distances from lattice QCD. *JHEP* **12,** 215. arXiv: 2106.15293 [hep-lat] (2021).
128. Chetyrkin, K. G. & Maier, A. Massless correlators of vector, scalar and tensor currents in position space at orders α_s^3 and α_s^4: Explicit analytical results. *Nucl. Phys. B* **844,** 266–288. arXiv: 1010.1145 [hep-ph] (2011).
129. Jegerlehner, F. & Szafron, R. $\rho^0 - \gamma$ mixing in the neutral channel pion form factor F_π^e and its role in comparing e^+e^- with τ spectral functions. *Eur. Phys. J. C* **71,** 1632. arXiv: 1101.2872 [hep-ph] (2011).
130. Sakurai, J. Theory of strong interactions. *Annals Phys.* **11,** 1–48 (1960).
131. Chakraborty, B. *et al.* The hadronic vacuum polarization contribution to a_μ from full lattice QCD. *Phys. Rev.* **D96,** 034516. arXiv: 1601.03071 [hep-lat] (2017).
132. Lee, W.-J. & Sharpe, S. R. Partial flavor symmetry restoration for chiral staggered fermions. *Phys. Rev.* **D60,** 114503. arXiv: hep-lat/9905023 [hep-lat] (1999).
133. Sharpe, S. R. & Van de Water, R. S. Staggered chiral perturbation theory at next-to-leading order. *Phys. Rev.* **D71,** 114505. arXiv: hep-lat/0409018 [hep-lat] (2005).
134. Aubin, C. & Blum, T. Calculating the hadronic vacuum polarization and leading hadronic contribution to the muon anomalous magnetic moment with improved staggered quarks. *Phys. Rev.* **D75,** 114502. arXiv: hep-lat/0608011 [hep-lat] (2007).
135. Lehner, C. *RBRC Workshop on Lattice Gauge Theories (2016).*
136. Borsányi, S. *et al.* Slope and curvature of the hadronic vacuum polarization at vanishing virtuality from lattice QCD. *Phys. Rev.* **D96,** 074507. arXiv: 1612.02364 [hep-lat] (2017).

137. Bazavov, A. *et al.* Hadronic Vacuum Polarization for the Muon $g-2$ from Lattice QCD: Long-Distance and Full Light-Quark Connected Contribution. *Phys. Rev. Lett.* **135,** 011901. arXiv: 2412.18491 [hep-lat] (2025).

138. Giusti, D., Lubicz, V., Martinelli, G., Sanfilippo, F. & Simula, S. Electromagnetic and strong isospin-breaking corrections to the muon $g-2$ from Lattice QCD+QED. *Phys. Rev.* **D99,** 114502. arXiv: 1901.10462 [hep-lat] (2019).

139. Colquhoun, B., Dowdall, R. J., Davies, C. T. H., Hornbostel, K. & Lepage, G. P. Υ and Υ' Leptonic Widths, a_μ^b and m_b from full lattice QCD. *Phys. Rev.* **D91,** 074514. arXiv: 1408.5768 [hep-lat] (2015).

140. Gounaris, G. J. & Sakurai, J. J. Finite width corrections to the vector meson dominance prediction for $\rho \to e^+e^-$. *Phys. Rev. Lett.* **21,** 244–247 (1968).

141. De Troconiz, J. F. & Yndurain, F. J. Precision determination of the pion form-factor and calculation of the muon $g-2$. *Phys. Rev. D* **65,** 093001. arXiv: hep-ph/0106025 (2002).

142. De Troconiz, J. F. & Yndurain, F. J. The Hadronic contributions to the anomalous magnetic moment of the muon. *Phys. Rev. D* **71,** 073008. arXiv: hep-ph/0402285 (2005).

143. Omnes, R. On the Solution of certain singular integral equations of quantum field theory. *Nuovo Cim.* **8,** 316–326 (1958).

144. Francis, A., Jaeger, B., Meyer, H. B. & Wittig, H. A new representation of the Adler function for lattice QCD. *Phys. Rev. D* **88,** 054502. arXiv: 1306.2532 [hep-lat] (2013).

145. Colangelo, G., Hoferichter, M. & Stoffer, P. Two-pion contribution to hadronic vacuum polarization. *JHEP* **02,** 006. arXiv: 1810.00007 [hep-ph] (2019).

146. Niehus, M., Hoferichter, M., Kubis, B. & Ruiz de Elvira, J. Two-Loop Analysis of the Pion Mass Dependence of the ρ Meson. *Phys. Rev. Lett.* **126,** 102002. arXiv: 2009.04479 [hep-ph] (2021).

147. Colangelo, G., Hoferichter, M., Kubis, B., Niehus, M. & de Elvira, J. R. Chiral extrapolation of hadronic vacuum polarization. *Phys. Lett. B* **825,** 136852. arXiv: 2110.05493 [hep-ph] (2022).

148. Neff, H., Eicker, N., Lippert, T., Negele, J. W. & Schilling, K. On the low fermionic eigenmode dominance in QCD on the lattice. *Phys. Rev.* **D64,** 114509. arXiv: hep-lat/0106016 [hep-lat] (2001).

149. Giusti, L., Hernandez, P., Laine, M., Weisz, P. & Wittig, H. Low-energy couplings of QCD from current correlators near the chiral limit. *JHEP* **04,** 013. arXiv: hep-lat/0402002 [hep-lat] (2004).

150. DeGrand, T. A. & Schaefer, S. Improving meson two point functions in lattice QCD. *Comput. Phys. Commun.* **159,** 185–191. arXiv: hep-lat/0401011 [hep-lat] (2004).

151. Li, A. *et al.* Overlap Valence on 2+1 Flavor Domain Wall Fermion Configurations with Deflation and Low-mode Substitution. *Phys. Rev.* **D82,** 114501. arXiv: 1005.5424 [hep-lat] (2010).

152. Giusti, L., Harris, T., Nada, A. & Schaefer, S. Frequency-splitting estimators of single-propagator traces. *Eur. Phys. J. C* **79,** 586. arXiv: 1903.10447 [hep-lat] (2019).

153. Aguillard, D. P. *et al.* Measurement of the Positive Muon Anomalous Magnetic Moment to 0.20 ppm. *Phys. Rev. Lett.* **131,** 161802. arXiv: 2308.06230 [hep-ex] (2023).

154. Ignatov, F. V. *et al.* Measurement of the Pion Form Factor with CMD-3 Detector and its Implication to the Hadronic Contribution to Muon $(g-2)$. *Phys. Rev. Lett.* **132,** 231903. arXiv: 2309.12910 [hep-ex] (2024).

155. Leplumey, T. P. & Stoffer, P. Dispersive analysis of the pion vector form factor without zeros. arXiv: 2501.09643 [hep-ph] (Jan. 2025).

156. Ablikim, M. *et al.* Measurement of the $e^+e^- \to \pi^+\pi^-$ cross section between 600 and 900 MeV using initial state radiation. *Phys. Lett. B* **753.** [Erratum: Phys.Lett.B 812, 135982 (2021)], 629–638. arXiv: 1507.08188 [hep-ex] (2016).

157. Akhmetshin, R. R. *et al.* Reanalysis of hadronic cross-section measurements at CMD-2. *Phys. Lett. B* **578,** 285–289. arXiv: hep-ex/0308008 (2004).

158. Aul'chenko, V. M. *et al.* Measurement of the pion form-factor in the range 1.04-1.38 GeV with the CMD-2 detector. *JETP Lett.* **82,** 743–747. arXiv: hep-ex/0603021 (2005).

159. Aul'chenko, V. M. *et al.* Measurement of the $e^+e^- \to \pi^+\pi^-$ cross section with the CMD-2 detector in the 370-520 MeV c.m. energy range. *JETP Lett.* **84,** 413–417. arXiv: hep-ex/0610016 (2006).

160. Akhmetshin, R. R. *et al.* High-statistics measurement of the pion form factor in the rho-meson energy range with the CMD-2 detector. *Phys. Lett. B* **648,** 28–38. arXiv: hep-ex/0610021 (2007).

161. Achasov, M. N. *et al.* Update of the $e^+e^- \to \pi^+\pi^-$ cross-section measured by SND detector in the energy region $400 < \sqrt{s} < 1000$ MeV. *J. Exp. Theor. Phys.* **103,** 380–384. arXiv: hep-ex/0605013 (2006).

162. Achasov, M. N. *et al.* Measurement of the $e^+e^- \to \pi^+\pi^-$ process cross section with the SND detector at the VEPP-2000 collider in the energy region $0.525 < \sqrt{s} < 0.883$ GeV. *JHEP* **01,** 113. arXiv: 2004.00263 [hep-ex] (2021).

163. Xiao, T., Dobbs, S., Tomaradze, A., Seth, K. K. & Bonvicini, G. Precision Measurement of the Hadronic Contribution to the Muon Anomalous Magnetic Moment. *Phys. Rev. D* **97,** 032012. arXiv: 1712.04530 [hep-ex] (2018).
